# Precise periodic components estimation for chronobiological signals through Bayesian Inference with sparsity enforcing prior

**DOI:** 10.1186/s13637-015-0033-6

**Published:** 2016-01-20

**Authors:** Mircea Dumitru, Ali Mohammad-Djafari, Simona Baghai Sain

**Affiliations:** 1 Laboratoire des signaux et systèmes (L2S), UMR 8506 CNRS–CentraleSupélec–Univ. Paris-Sud, CentraleSupélec, Plateau de Moulon, Gif-sur-Yvette, 91192 France; 2 Rythmes Biologiques et Cancers (RBC), UMR 776 INSERM–Univ. Paris-Sud, Campus CNRS, Villejuif, 94801 France; 3 Department of Molecular Biotechnology and Health Sciences, University of Turin, Turin, 10126 Italy

**Keywords:** Periodic component (PC) vector estimation, Sparsity enforcing, Bayesian parameter estimation, Variational Bayesian approximation (VBA), Kullback-Leibler (KL) divergence, Infinite Gaussian scale mixture (IGSM), Normal-inverse gamma, Inverse problem, Joint maximum a posteriori (JMAP), Posterior mean (PM), Chronobiology, Circadian rhythm, Cancer treatment

## Abstract

The toxicity and efficacy of more than 30 anticancer agents present very high variations, depending on the dosing time. Therefore, the biologists studying the circadian rhythm require a very precise method for estimating the periodic component (PC) vector of chronobiological signals. Moreover, in recent developments, not only the dominant period or the PC vector present a crucial interest but also their stability or variability. In cancer treatment experiments, the recorded signals corresponding to different phases of treatment are short, from 7 days for the synchronization segment to 2 or 3 days for the after-treatment segment. When studying the stability of the dominant period, we have to consider very short length signals relative to the prior knowledge of the dominant period, placed in the circadian domain. The classical approaches, based on Fourier transform (FT) methods are inefficient (i.e., lack of precision) considering the particularities of the data (i.e., the short length). Another particularity of the signals considered in such experiments is the level of noise: such signals are very noisy and establishing the periodic components that are associated with the biological phenomena and distinguishing them from the ones associated with the noise are difficult tasks. In this paper, we propose a new method for the estimation of the PC vector of biomedical signals, using the biological prior informations and considering a model that accounts for the noise. The experiments developed in cancer treatment context are recording signals expressing a limited number of periods. This is a prior information that can be translated as the sparsity of the PC vector. The proposed method considers the PC vector estimation as an Inverse Problem (IP) using the general Bayesian inference in order to infer the unknown of our model, i.e. the PC vector but also the hyperparameters (i.e the variances). The sparsity prior information is modeled using a sparsity enforcing prior law. In this paper, we propose a Student’s *t* distribution, viewed as the marginal distribution of a bivariate normal-inverse gamma distribution. We build a general infinite Gaussian scale mixture (IGSM) hierarchical model where we assign prior distributions also for the hyperparameters. The expression of the joint posterior law of the unknown PC vector and hyperparameters is obtained via Bayes rule, and then, the unknowns are estimated via joint maximum a posteriori (JMAP) or posterior mean (PM). For the PM estimator, the expression of the posterior distribution is approximated by a separable one, via variational Bayesian approximation (VBA), using the Kullback-Leibler (KL) divergence. For the PM estimation, two possibilities are considered: an approximation with a partially separable distribution and an approximation with a fully separable one. Both resulting algorithms corresponding to the PM estimation and the one corresponding to the JMAP estimation are iterative algorithms. The algorithms are presented in detail and are compared with the ones corresponding to the Gaussian model. We examine the convergency of the algorithms and give simulation results to compare their performances. Finally, we show simulation results on synthetic and real data in cancer treatment applications. The real data considered in this paper examines the rest-activity patterns of KI/KI Per2::luc mouse, aged 10 weeks, singly housed in RealTime Biolumicorder (RT-BIO).

**Electronic supplementary material:**

The online version of this article (doi:10.1186/s13637-015-0033-6) contains supplementary material, which is available to authorized users.

## Introduction

Several biological processes in living organisms follow oscillations that repeat themselves about every 24 h—these oscillations are called circadian rhythms and together with other periodic phenomena, they are the object of study of chronobiology [1–3]. In mammals, circadian rhythms involve all organs, tissues, and cells and are supervised by the circadian timing system (CTS), a set of molecular clock genes that cross-regulate each other by positive and negative feedback loops [4–6]. More precisely, the CTS consists of a central pacemaker, the suprachiasmatic nuclei (SCN) in the hypothalamus, which is made sensitive to light by retinal afferents and which coordinates the molecular clocks in the peripheral organs by releasing diffusible and neurophysiological signals [3]. The period of the CTS, which is about 24 h, is therefore regularly calibrated by the succession of light and day and can be influenced by other environmental factors, such as socio-professional interactions and feeding times [5]. The resulting circadian physiologic fluctuations are observed in sleep-wakefulness and rest-activity alternations, body temperature, cortisol secretion by the adrenal gland, and melatonin secretion by the pineal gland, and they involve as well the sympathetic and the parasympathetic systems [6].

Former studies have already shown how taking chronobiology into account can improve anticancer treatment efficacy and reduce at the same time their toxicity (increasing therefore their tolerability), contrary to the previous “the worst the toxicity, the better the efficacy” paradigm [7–10]. The molecular clocks are involved in the regulation of important processes such as cell cycle and proliferation, DNA damage sensing and repair, apoptosis, angiogenesis, pharmacodynamics, and pharmacokinetics; therefore, they can greatly influence the metabolism, transportation, and detoxification of drugs [11].

Tolerability to anticancer treatments has been proven to depend significantly on their timing in respect to the circadian rhythms, measuring up to tenfold changes in the tolerability to drug administration at different circadian times for 40 anticancer drugs in rodents and up to fivefold in patients [11, 12]. Notably, chemotherapeutic agents proved to be at their best efficacy, both administered alone and combined, when they are also at their best tolerability level, i.e., when they are least toxic to the healthy tissues. Furthermore, relevant interpatient variability in circadian rhythms have been observed and can be due to factors such as gender, age, and genetic polymorphisms; therefore, anticancer drugs dosing and timing need to be personalized, at least for subtypes of patients with similar chronotoxicity key features. Modulating drugs administration according to the patient’s circadian rhythms is known as chronotherapy [13, 14]. On the other hand, administrating anticancer drugs at their most toxic time causes the disruption of molecular clocks synchronization, which has been shown to accelerate the cancer evolution [15–20].

In order to optimize cancer treatment, once proven that a certain drug effects are susceptible to circadian rhythms, we want to identify its best administration time. First, for each drug is proved the correlation with the circadian rhythms in a rodent model, which has been proved to well represent the human circadian physiology [11]. This is achieved by studying the chronotoxicity of the drug, inferred by body weight loss and histopathologic lesions, at different circadian times (CT or ZT, from Zeitgeber time). The mice circadian clock is synchronized by exposure to light for 12 h, followed by 12 h of dark, repeating this cycle and its rhythm is detected by tracking the expression of one or more of its core genes (normally Bmal1, Per2, Rev-erb *α*, or Clock are used). Mice with a disrupted clock (clock-defective mice, obtained via the functional knock out of one of its genes, normally Per2) are used to confirm the relevance of the molecular clock for the drug toxicity. At the same time, the main characteristics of the circadian expression of these observed genes are studied to observe whether the administration of the drug modifies them.

Once defined the CTs at which the drug best and worst tolerability is observed, we can look for the molecular mechanisms that influence it. Genes influencing the pharmacokinetics (absorption, distribution, metabolism, and excretion) of the drug are a good starting point, and we can follow how their expression correlates with the higher or lower drug chronotoxicity. For instance, the transporter abcc2, involved in the cellular efflux of several drugs, has been shown to influence irinotecan chronotolerance in ileum, according to the circadian changes in abcc2 local expression [21]. The circadian clocks of the mice used in the experiments whose data we analyse are first synchronized to the same day-night alternation where 12 h of light are followed by 12 h of dark (LD12:12). After synchronization, the mice are kept in constant darkness (DD), which implies the subtraction of the light. Throughout the experience, gene expression and rest-activity are measured to establish how the basic parameters of their circadian rhythms (period, acrophase, amplitude) vary in respect to the drug treatment. Both measures are allowed by an innovative monitoring device, the RealTime-Biolumicorder (RT-BIO) [22]. The locomotor activity is detected by an infrared sensor, whereas the gene expression is measured at the post-translational level in mice engineered to express the gene of interest together with luciferase (fLUC), so that the gene activity is marked by bioluminescence detected by a photomultiplier tube. Common mouse strains used are C57BL/6-based [7, 21] and 129S1/SvImJ [23]. The acrophase and amplitude depend on the periodic component (PC) vector, so a major interest is the study of the periodicity of such time series, i.e., the estimation of the PC vector and the stability or the variability of the dominant period, requiring a precise PC vector variation analysis. The periodical phenomena were studied with different approaches in different particular conditions [24–40] using in general fast Fourier transform (FFT)-based methods. The major limitation when studying such data is given by their reduced length, due to the duration of the experiments. The objective of an accurate description of the periodic components variation during the experiments can be formulated as the need of a method that can give a precise estimation of the PC vector from a limited number of data. Also, the method must be able to distinguish the peaks from the PC vector due to the biological phenomena and the peaks due to the measurements errors. The real data considered in this article is a chronobiological time series, measuring the locomotor activity. In order to observe the variation or the stability of the dominant periods, very short intervals of the recorded time series are considered. The prior knowledge is the presence of the circadian rhythm: the PC vector is sparse, having a limited number of non-zero elements, inside the circadian interval.

The article is positioned in the context of the need of a method capable to estimate the PC vector of a time series in the following conditions: (a) very limited number of data (4-day length) for circadian periodic components (24 ± 6 h) estimation and (b) precision that can be adjusted depending on the chronobiological context, 1-hour precision required in the particular experiment discussed in this article. The method proposed in this article formulates the estimation of the PC vector as an inverse problem, using the general Bayesian inference to infer the unknowns of the considered linear model. This approach is presented in Section 3. A hierarchical prior model is considered, using the Student’s *t* distribution (expressed as the marginal of a normal-inverse gamma bivariate distribution) as the sparsity enforcing prior law for the PC vector and assigning prior distributions for the hyperparameters involved in the model, namely the variances associated with the PC vector and the noise (Subsection 3.2 and Section 4). From the analytical expression of the joint posterior law of the unknown PC vector and hyperparameters, obtained via Bayes rule, the unknowns are estimated via joint maximum a posteriori (JMAP) (Subsection 4.1) or posterior mean (PM). For the PM estimator, the expression of the posterior law is approximated by a separable one, via the variational Bayesian approximation (VBA), using the Kullback-Leibler (KL) divergence. For the PM estimation, two possibilities are considered: an approximation with a partially separable law (Subsection 4.2) and one with a full separable one (Subsection 4.3). Simulation results on synthetic data (5 dB) and real data in cancer treatment applications are presented in Section 5. More simulations for the synthetic case (10 and 15 dB) are presented in the Additional file 1.

## Classical Fourier transform methods

**Fig. 1:**
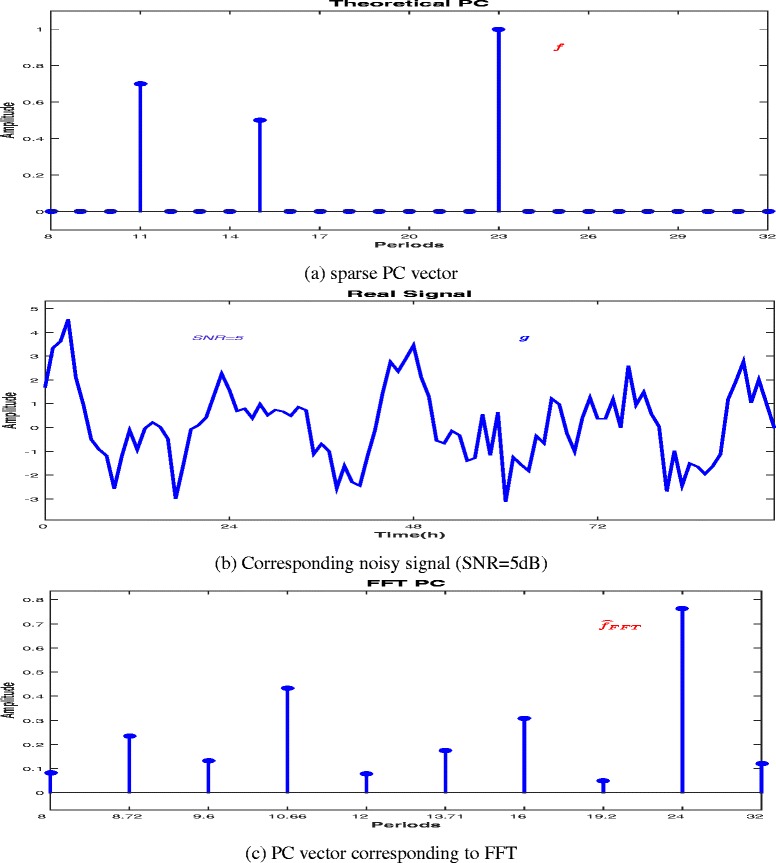
Synthetic data. Sparse theoretical PC vector with 3 non-zero peaks corresponding to 11, 15, and 23 h (**a**). The corresponding noisy signal (SNR = 05 dB) (**b**). PC vector corresponding to FFT (**c**)

The spectral analysis for time series is a well-known subject in literature for a very long time. The most used methods are the FFT-based methods, which are widely used for many applications in signal processing community, having obvious advantages: the FFT-based methods are well known, well understood, and fast. Nevertheless, the particularities of the biomedical signals considered in chronobiology experiments show that the classical methods present certain limitations. In particular, for short time series relative to the dominant period (in the experiment considered in this article, a 96 h recorded signal relative to an ∼24 h dominant period, linked with the circadian clock) the precision given by the FFT methods is by far insufficient to determine the exact PC vector, since via the FFT-based methods, the frequency axis is linear, but as a function of the periods, it is not. In particular, for a 4-day (96 h) recorded signal, beside the 24 h corresponding periodic component, the nearest amplitudes in the PC vector correspond to 32 and 19 h (Fig. 1).

More general, if the prior knowledge sets the dominant period around a value *P* in order to obtain a PC vector that contains the period *P* and also the periods *P* − 1 and *P* + 1, the signal must be observed for (*P*−1)(*P*+1) periods. In chronobiology applications, where the circadian period is around 24 h, a signal should be recorded for 575 days in order to obtain a periodic component vector that contains 23-, 24-, and 25-h periods.

**Fig. 2:**
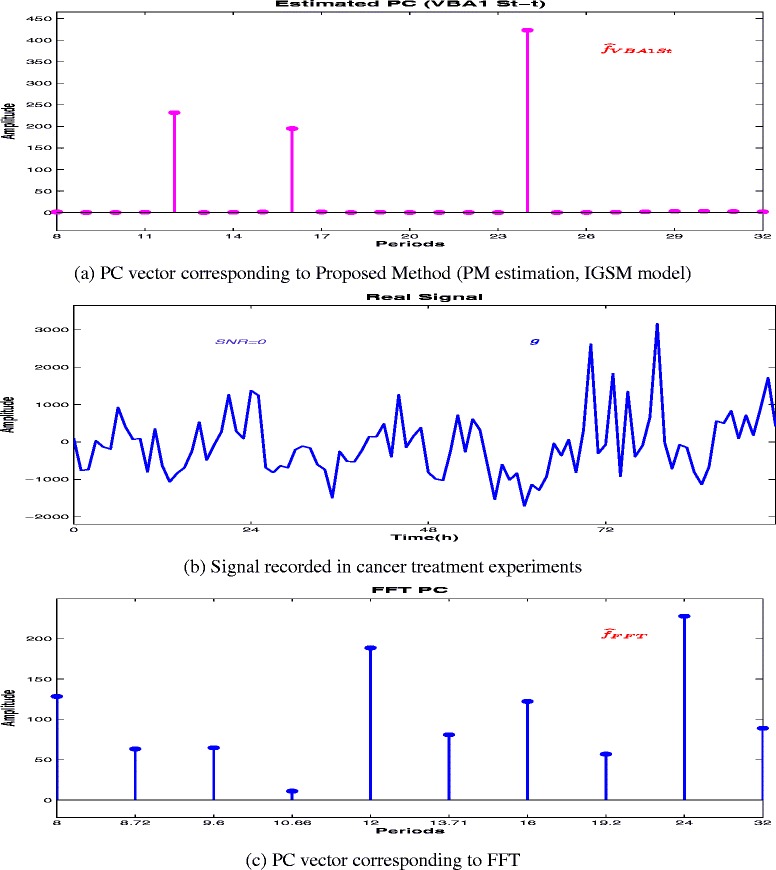
Real data. We show a 4-day-length signal recorded in cancer treatment experiments (**b**) and the PC vector corresponding to FFT (**c**) and corresponding to the proposed method (**a**)

As an example, Fig. 1 b presents a 4 day synthetic signal corresponding to a known PC vector (showed in Fig. 1 a) and the corresponding PC vector obtained via FFT (Fig. 1 c) (presented for the interval between 8 and 32 h, the circadian domain). In the synthetic PC vector, the non-zero periods are set for 11, 15, and 23 h (dominant peak). The FFT estimates the dominant peak at 24 h, due to the fact that the time series observation period is limited to 96 h. In such conditions, it offers no information for the real positions, 11, 15, 23 h. It also offers no informations for the peaks in the interval [ 20:31], except the estimation for 24 h. For similar signals corresponding to PC vectors having the principal peak around 24 h, the FFT will estimate the principal peak at 24 h. Another example is presented in Fig. 2 b, a 4-day-length signal recorded in an experiment in chronobiology. The FFT PC vector presents peaks corresponding only to 8, 8.72, 9.6, 10.66, 12, 13.71, 16, 19.2, 24, and 32 h inside the considered interval [ 8:32]. The periods corresponding to 24, 12, and 8 h can be associated with the presence of the circadian rhythm, expressed by the principal peak and the corresponding harmonics. But the presence of the other peaks can be more difficult to be interpreted by the biologists. Another drawback of FFT is the difficulty of selecting the peaks corresponding to the presence of a biological phenomena and peaks that are explained by error measures and uncertainties. This article is proposing a method that can estimate the PC vector, in the conditions (a) and (b) described in Section 1, taking into account the uncertainties and being able to distinguish between the peaks: the ones corresponding to the biological phenomena and the ones explained by other factors, producing a sparse PC vector (Fig. 2 a). We think that this result will be more comprehensible by the biologists.

## Inverse problem approach and general Bayesian inference

The first stepa in the proposed method for improving the precision consists in the inverse problem approach. We formulate the relation between the available data ***g*** and the unknown PC vector ***f*** as an inverse problem. The inverse Fourier transform provides a linear relation between the known biomedical signal ***g*** and the PC vector ***f***. Using the elements of the matrix corresponding to the inverse Fourier transform, the linear relation is described by the following equation:

(1) $${} {g}(t_{i}) \simeq \sum\limits_{j=1}^{M} {{f}(p_{j})}{e^{2 \pi j \frac{1}{p_{j}} t_{i}}},\quad i \in \left\{ 1,\ldots,N \right\},\quad\!\! j \in \left\{ 1,\ldots,M \right\}.   $$

Introducing the notations *g*(*t*_*i*_)=*g*_*i*_ and *f*(*t*_*j*_)=*f*_*j*_, Eq. (1) becomes

(2) $${} {\fontsize{8.8pt}{9.6pt}{\begin{aligned} {g}_{i} \simeq \sum\limits_{j=1}^{M} {{f}_{j}}{e^{2 \pi j \frac{1}{{p}_{j}} {t_{i}}}},\quad i \in \left\{ 1,\ldots,N\right\} \quad j \in \left\{ 1,\ldots,M \right\} \rightarrow{} \boldsymbol{g} \simeq \boldsymbol{H}\, \boldsymbol{f}. \end{aligned}}}   $$

Due to the potential modeling and measurement errors, we need to account for errors and uncertainties, so the linear model given by the inverse Fourier transform is completed by introducing the error vector ***ε***, obtaining the forward model, Eq. (3):

(3) $$ \boldsymbol{g}=\boldsymbol{H} \,\boldsymbol{f} + \boldsymbol{\epsilon},   $$

where we have used the following notations:

- ***g*** represents the observed data, i.e., the chronobiological time series: $\boldsymbol {g}\;=\; \left [{g}_{1}, {g}_{2} \ldots {g}_{{N}}\right ]^{T} \in \mathcal {M}_{N\times 1},$ an *N*-dimensional vector
- ***f*** represents the unknowns, i.e., the PC vector: $\boldsymbol {f}\;=\;\left [{f}_{1}, {f}_{2}, \ldots, {f}_{M}\right ]^{T} \in \mathcal {M}_{M \times 1},$ a *M*-dimensional vector
- ***ε*** represents the errors: $\boldsymbol {\epsilon }\;=\;\left [{\epsilon }_{1}, {\epsilon }_{2}, \ldots, {\epsilon }_{N}\right ]^{T} \in \mathcal {M}_{N \times 1},$ is an *N*-dimensional vector

The goal is to estimate the unknowns of the model, Eq. (3), i.e., the PC vector ***f*** and the error vector ***ε***. In this paper, we propose an inversion based on general Bayesian inference, building a hierarchical model and estimating the unknowns from the posterior probability density function, using the available data ***g***.

The estimated ${\widehat {\boldsymbol {f}}}$ and the corresponding estimated signal ${\widehat {\boldsymbol {g}}}=\boldsymbol {H}{\widehat {\boldsymbol {f}}}$ are compared with ***f*** (only in the synthetic case) and ***g***, using as a measure of performance Eq. (4).

(4) $$ \delta \boldsymbol{f} = \frac{\left\| \boldsymbol{f} - {\widehat{\boldsymbol{f}}} \right\|_{2}^{2}}{\left\|\boldsymbol{f}\right\|_{2}^{2}} \;\; ; \;\; \delta \boldsymbol{g} = \frac{\left\|\boldsymbol{g}-\widehat{\boldsymbol{g}}\right\|_{2}^{2}}{\left\|\boldsymbol{g}\right\|_{2}^{2}}.   $$

For the application considered in this paper, the matrix ***H*** used in the model presented in Eq. (3) has very high conditioning numbers, so the problem is ill-conditioned. As mentioned above, in this paper, we focus on an inversion based on general Bayesian inference. Nevertheless, in literature, many other approaches are possible. One particular case of the considered linear model is the case where the error vector is neglected (***ε***=**0**), and the matrix ***H*** is invertible and orthogonal, i.e., ***H*** ^*T*^ ***H***=***I***. This is the case of the FT matrix with *M*=*N*. Then, the solution is given by ${\widehat {\boldsymbol {f}}}=\boldsymbol {H}^{T}\boldsymbol {g}$, which corresponds to IFT. However, in general as in our case *M*≠*N*. When *M*<*N*, a minimum norm solution ${\widehat {\boldsymbol {f}}}_{\text {MN}} = \left (\boldsymbol {H} \boldsymbol {H}^{T} \right)^{-1} \boldsymbol {H}^{T} \boldsymbol {g}$ can be obtained, and when *M*>*N*, the classical solution is the least square solution ${\widehat {\boldsymbol {f}}}_{\text {LS}} = \boldsymbol {H}^{T} \left (\boldsymbol {H} \boldsymbol {H}^{T} \right)^{-1} \boldsymbol {g}$. Since in the case of chronobiological times series the matrix ***H*** is proved to have a very high conditioning number, those generalized inverse solutions are, in general, too sensitive to the errors due to the ill-conditioning of the matrix ***H***. The regularization methods can partially solve this difficulty. For example, the regularization methods such as truncated single value decomposition (TSVD) or Tikhonov regularization methods (TRM) can be used, but the solutions depend on the threshold in the first case (TSVD) and on the regularization parameter in the second case. When *M*≠*N* and when the error vector is not neglected (***ε***≠**0**), the regularization methods can still be applied and an estimation can be obtained for ***f*** and ***ε***, but with the following drawbacks: in general, determining the regularization parameters is difficult and there is not a good way to handle other a priori knowledge we may have on the noise statistics and on the unknowns.

### Bayesian inference

A fundamental particularity of the proposed method is the use of the prior knowledge. In this article, we adopt a Bayesian approach. The Bayesian approach for times series was considered in [41–53]. However, the lack of data makes the proposed methods inefficient for our case. In Bayesian inference, the fundamental relation is given by the Bayes rule:

(5) $$ p\!\left(\boldsymbol{f}|\boldsymbol{g},\boldsymbol{\theta}_{1},\boldsymbol{\theta}_{2}\right) = \frac{p\!\left(\boldsymbol{g}|\boldsymbol{f},\boldsymbol{\theta}_{1}\right)\, p\!\left(\boldsymbol{f}|\boldsymbol{\theta}_{2}\right)}{p\!\left(\boldsymbol{g}|\boldsymbol{\theta}_{1},\boldsymbol{\theta}_{2}\right)}, \boldsymbol{\theta} = \left(\boldsymbol{\theta}_{1},\boldsymbol{\theta}_{2}\right),   $$

where ***θ*** represents the hyperparameters that appear in the model.

In general, we may not know the hyperparameters ***θ***, and this is also our case. The hyperparameters represents the variances associated with the noise ***ε*** and with ***f***, which are unknown. We need to estimate them, too. This can be done via the joint posterior law:

(6) $$ p\!\left(\boldsymbol{f},{\theta}_{1}, {\theta}_{2}|\boldsymbol{g}\right) \propto p\!\left(\boldsymbol{g}|\boldsymbol{f},{\theta}_{1}\right)\, p\!\left(\boldsymbol{f}|{\theta}_{2}\right)\, p({\theta}_{1})\, p\!\left({\theta}_{2}\right).   $$

Such an extension presents two particular advantages: one advantage is evidently the possibility of estimating the hyperparameters and obtaining numerical values for variances and the second one is that such an approach can be developed into a non-supervised algorithm.

### Hierarchical prior models

The hierarchical model represents the set of probability density functions assigned for the probabilities involved in (6), namely the assignment of the prior *p* (***g***|***f***,***θ***_1_), the likelihood *p* (***f***|***θ***_2_), and the hyperparameters priors *p*(***θ***_1_),*p*(***θ***_2_).

The prior biological knowledge leads to the search of good sparsity enforcing priors. In literature [54], certain classes of distribution (heavy-tailed, mixture models) are well known as good sparsity enforcing priors. In this paper, we consider a general infinite Gaussian scale mixture (IGSM) hierarchical model [55]. The prior distribution for the PC vector is a Student’s *t* distribution expressed via a normal-inverse gamma distribution. The error vector is also modeled using the IGSM, considering non-stationary variances for the noise, generalizing the results from [56]. In Section 5, during the simulations results, we include comparisons with the Gaussian hierarchical model for the synthetic data.

## Hierarchical model infinite Gaussian scale mixture

In the first step, we model the error vector ***ε***. We propose to use a non-stationary Gaussian model:

(7) $$ p\!\left({\epsilon}_{i}|{v}_{{\epsilon}_{i}}\right) = \mathcal{N}\! \left({\epsilon}_{i}|0,{v}_{{\epsilon}_{i}}\right), \; i \in \left\{ 1, 2, \ldots, N \right\},  $$

where $\phantom {\dot {i}\!}{{v}_{{{\epsilon }_{i}}}}$ are considered to be unknowns. For having the possibility to estimate them, we model them as inverse gamma distributions:

(8) $$ p\!\left({v}_{{\epsilon}_{i}}|\alpha_{\epsilon 0},\beta_{\epsilon 0}\right) =\mathcal{I}\mathcal{G}\! \left(v_{{\epsilon}_{i}}|\alpha_{\epsilon 0},\beta_{\epsilon 0}\right),\; i \in \left\{ 1, 2, \ldots, N \right\}.  $$

Doing this, we model the error vector as an infinite Gaussian scale mixture:

(9) $$ \left\{ \begin{array}{l} p\!\left(\boldsymbol{\epsilon}|\boldsymbol{v}_{\boldsymbol{\epsilon}}\right) = \mathcal{N}\!\left(\boldsymbol{\epsilon} | \boldsymbol{0}, \boldsymbol{V}_{\boldsymbol{\epsilon}}\right) \\ p\!\left(\boldsymbol{v}_{\boldsymbol{\epsilon}}|\alpha_{\epsilon 0},\beta_{\epsilon 0}\right) = \prod_{i=1}^{N} \mathcal{I}\mathcal{G}\! \left(v_{{\epsilon}_{i}}|\alpha_{\epsilon 0},\beta_{\epsilon 0}\right),\\ \end{array}\right.   $$

where we introduced the vector ***v***_***ε***_ and the corresponding diagonal matrix ***V***_***ε***_:

(10) $$ \boldsymbol{v}_{\boldsymbol{\epsilon}}\;=\; \left[{v}_{{\epsilon}_{1}} \ldots {v}_{{\epsilon}_{i}} \ldots {v}_{{\epsilon}_{N}} \right]^{T} \;\; ; \;\; \boldsymbol{V}_{\boldsymbol{\epsilon}} = \text{diag} \left[\boldsymbol{v}_{\boldsymbol{\epsilon}}\right].\;   $$

The likelihood *p* (***g***|***f***,***v***_***ε***_) is obtained using the considered linear model, Eq. (3), and the assigned distribution for the error vector ***ε*** conditioned by the variance ***v***_***ε***_, Eq. (9). The distribution modeling the likelihood is also a multivariate normal distribution, having the same covariance matrix ***V***_***ε***_ and the mean ***H*** ***f***:

(11) $$ p\!\left(\boldsymbol{g}|\boldsymbol{f},\boldsymbol{v}_{\boldsymbol{\epsilon}}\right) = \mathcal{N}\!\left(\boldsymbol{g}|\boldsymbol{H}\boldsymbol{f},\boldsymbol{V}_{\boldsymbol{\epsilon}}\right).   $$

The proposed prior distribution is a Student’s *t* distribution, in order to enforce the sparsity and use the prior knowledge of reduced number of periods in the PC vector. While a direct assignment of a Student’s *t* distribution for the prior law *p*(***f***) leads to a non-quadratic criterion when estimating ***f***, the Student’s *t* distribution corresponding to the prior law can be expressed as an infinite Gaussian scale mixture, modeling the inverse variance as a gamma distribution or the variance as an inverse gamma distribution. For the variance of ***f***, we assume a general model:

(12) $$ \boldsymbol{v}_{\boldsymbol{f}}\;=\; \left[{v}_{{f}_{1}} \ldots {v}_{{f}_{{j}}} \ldots {v}_{{f}_{{M}}}\right]^{T} \;\; ; \;\; \boldsymbol{V}_{\boldsymbol{f}} = \text{diag} \left[{\boldsymbol{v}_{\boldsymbol{f}}}\right].\;   $$

The prior law is then defined as an infinite Gaussian scale mixture, via ***v***_***f***_:

(13) $$ \left\{ \begin{array}{l} p\!\left(\boldsymbol{f}|\boldsymbol{v}_{\boldsymbol{f}}\right) = \mathcal{N}\!\left(\boldsymbol{f}|\boldsymbol{0},\boldsymbol{V}_{\boldsymbol{f}}\right) \\ p\!\left(\boldsymbol{v}_{\boldsymbol{f}}|\alpha_{f 0},\beta_{f 0}\right)=\prod_{j=1}^{M} \mathcal{I}\mathcal{G}\! \left(v_{f_{j}}|\alpha_{f 0},\beta_{f 0}\right). \end{array}\right.   $$

The error variance priors, Eq. (9), the likelihood, Eq. (11), and the prior, Eq. (13), represents the IGSM hierarchical model. The analytical form is presented in Eq. (14):

(14) $$ \left\{\begin{array}{ll} p\! \left(\boldsymbol{g}|\boldsymbol{f},\boldsymbol{v}_{\boldsymbol{\epsilon}}\right) = \mathcal{N}\! \left(\boldsymbol{g}|\boldsymbol{H}\, \boldsymbol{f},\boldsymbol{V}_{\boldsymbol{\epsilon}}\right) \propto {\det^{-\frac{1}{2}}} {(\boldsymbol{V}_{\boldsymbol{\epsilon}})} \; \exp \left\{ -\frac{1}{2} \| \boldsymbol{V}_{\boldsymbol{\epsilon}}^{-\frac{1}{2}} \left(\boldsymbol{g} - \boldsymbol{H}\, \boldsymbol{f}\right) \|^{2} \right\} \\ p\! \left(\boldsymbol{f}|\boldsymbol{v}_{\boldsymbol{f}}\right) = \mathcal{N}\!\left(\boldsymbol{f}| \boldsymbol{0},\boldsymbol{V}_{\boldsymbol{f}}\right) \propto \det^{-\frac{1}{2}} {(\boldsymbol{V}_{\boldsymbol{f}})} \; \exp \left\{ -\frac{1}{2} \| (\boldsymbol{V}_{\boldsymbol{f}})^{-\frac{1}{2}} \boldsymbol{f} \|^{2} \right\} \\ p\! \left(\boldsymbol{v}_{\boldsymbol{\epsilon}}|\alpha_{\epsilon 0},\beta_{\epsilon 0}\right) = {\prod_{i=1}^{N}}\, \mathcal{I}\mathcal{G}\! \left({v_{{\epsilon}_{i}}}|{\alpha_{\epsilon 0}},{\beta_{\epsilon 0}}\right) \propto {\prod_{i=1}^{N}}\, {v_{{\epsilon}_{i}}^{-(\alpha_{\epsilon 0}+1)}} \; \exp \left\{ {-\sum_{i=1}^{N}}\, {\beta_{\epsilon 0}} {v_{{\epsilon}_{i}}^{-1}} \right\} \\ p\! \left(\boldsymbol{v}_{\boldsymbol{f}}|\alpha_{f 0},\beta_{f 0} \right) = {\prod_{j=1}^{M}}\, \mathcal{I}\mathcal{G}\! \left({{v}_{{f}_{j}}}|\alpha_{f 0},\beta_{f 0}\right) \propto {\prod_{j=1}^{M}}\, {v_{{f}_{j}}^{-(\alpha_{f 0}+1)}} \; \exp \left\{ -{\sum_{j=1}^{M}}\, \beta_{f 0} {v_{{f}_{j}}^{-1}} \right\}. \\ \end{array}\right.   $$

From the hierarchical model, the posterior distribution can be obtained via the proportionality relation considered in Eq. (6):

(15) $${} \begin{aligned} p\!\left(\boldsymbol{f},\boldsymbol{v}_{\boldsymbol{\epsilon}},\boldsymbol{v}_{\boldsymbol{f}}|\boldsymbol{g}\right) \; \propto \; & p\!\left(\boldsymbol{g}|\boldsymbol{f},\boldsymbol{v}_{\boldsymbol{\epsilon}}\right) \; p\!\left(\boldsymbol{f}|\boldsymbol{v}_{\boldsymbol{f}}\right) \; p\!\left(\boldsymbol{v}_{\boldsymbol{\epsilon}}|\alpha_{\epsilon 0},\beta_{\epsilon 0}\right)\\& p\!\left(\boldsymbol{v}_{\boldsymbol{f}}|\alpha_{f 0},\beta_{f 0}\right). \end{aligned}  $$

### Joint MAP estimation

**Fig. 3:**
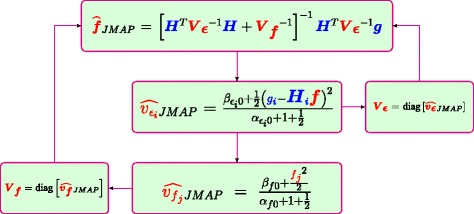
IGSM hierarchical model—JMAP estimation: iterative algorithm

The joint maximum a posteriori, a point estimator of the unobserved quantities ***f***, ***v***_***ε***_, ***v***_***f***_ on the basis of the available data ***g*** is defined as:

(16) $${} {\fontsize{9.2pt}{9.6pt}{\begin{aligned} \left({\widehat{\boldsymbol{f}}},\;{\widehat{\boldsymbol{v}_{\boldsymbol{\epsilon}}}},\;{\widehat{\boldsymbol{v}_{\boldsymbol{f}}}}\right) = {\underset{\left(\boldsymbol{f}, \; {\boldsymbol{v}_{\boldsymbol{\epsilon}}}, \; {\boldsymbol{v}_{\boldsymbol{f}}}\right)}{\arg\max}}\;\, p\!\left(\boldsymbol{f},\boldsymbol{v}_{\boldsymbol{\epsilon}}, \boldsymbol{v}_{\boldsymbol{f}}|\boldsymbol{g}\right) = {\underset{\left(\boldsymbol{f},\;{\boldsymbol{v}_{\boldsymbol{\epsilon}}}, \; {\boldsymbol{v}_{\boldsymbol{f}}} \right)}{\arg\min}}\; \mathcal{L}\!\left(\boldsymbol{f},\boldsymbol{v}_{\boldsymbol{\epsilon}},\boldsymbol{v}_{\boldsymbol{f}}\right), \end{aligned}}}  $$

where for the second equality, we have defined the criterion $\mathcal {L}\!\left (\boldsymbol {f},\boldsymbol {v}_{\boldsymbol {\epsilon }},\boldsymbol {v}_{\boldsymbol {f}}\right) = -\ln p\! \left (\boldsymbol {f},\boldsymbol {v}_{\boldsymbol {\epsilon }}, \boldsymbol {v}_{\boldsymbol {f}}|\boldsymbol {g}\right)$. The MAP estimator is the solution minimizing the criterion $\mathcal {L}\!\left (\boldsymbol {f},\boldsymbol {v}_{\boldsymbol {\epsilon }},\boldsymbol {v}_{\boldsymbol {f}}\right)$. This can be done via alternate optimization with respect to each of the unknowns. The computation details are presented in Appendix 1. Here, we present the final results in Eqs. (17a), (17b), and (17c).

(17a) $$\begin{array}{@{}rcl@{}} {\widehat{\boldsymbol{f}}_{\text{JMAP}}} & = & \left[\boldsymbol{H}^{T}{\boldsymbol{V}_{\boldsymbol{\epsilon}}^{-1}} \boldsymbol{H} + {\boldsymbol{V}_{\boldsymbol{f}}^{-1}} \right]^{-1} \boldsymbol{H}^{T}{\boldsymbol{V}_{\boldsymbol{\epsilon}}^{-1}} \boldsymbol{g}  \end{array} $$

(17b) $$\begin{array}{@{}rcl@{}} {\widehat{{v}_{{\epsilon}_{i}}}}_{\text{JMAP}} & = & \frac{\beta_{\epsilon_{i} 0} + \frac{1}{2} \left({g}_{i} - \boldsymbol{H}_{i}\ \boldsymbol{f} \right)^{2}}{\alpha_{\epsilon_{i} 0} + 1 + \frac{1}{2}}  \end{array} $$

(17c) $$\begin{array}{@{}rcl@{}} {\widehat{{v}_{{f}_{j}}}}_{\text{JMAP}} & = & \frac{\beta_{f 0} + \frac{{f}_{j}^{2}}{2}}{\alpha_{f 0} + 1 + \frac{1}{2}},  \end{array} $$

where ***H***_*i*_ represents the line *i* from the matrix ***H***. The iterative algorithm obtained via JMAP estimation is presented in Fig. 3. The algorithm is compared with the one corresponding to the posterior mean estimation in Section 5.

### Posterior mean (via VBA) IGSM (partial separability)

The posterior mean estimates the mean of the posterior distribution. One of the advantages of this estimator is the fact that it minimizes the mean square error (MSE). In particular, the posterior distribution obtained from the considered hierarchical model is not a separable distribution, making the analytical computation of PM very difficult. One way to compute the PM in this case is to first approximate the posterior law *p* (***f***,***v***_***ε***_,***v***_***f***_|***g***) with a separable law *q* (***f***,***v***_***ε***_,***v***_***f***_|***g***) [54]:

(18) $$ p\!\left(\boldsymbol{f},\boldsymbol{v}_{\boldsymbol{\epsilon}},\boldsymbol{v}_{\boldsymbol{f}}|\boldsymbol{g}\right) \approx q\!\left(\boldsymbol{f},\boldsymbol{v}_{\boldsymbol{\epsilon}},\boldsymbol{v}_{\boldsymbol{f}}|\boldsymbol{g}\right) = q_{1}(\boldsymbol{f}) \; q_{2}\left(\boldsymbol{v}_{\boldsymbol{\epsilon}}\right) \; q_{3}\left(\boldsymbol{v}_{\boldsymbol{f}}\right),   $$

where we have used the notations

(19) $$ q_{2}(\boldsymbol{v}_{\boldsymbol{\epsilon}}) = \prod\limits_{i=1}^{N} q_{2i}\!\left({v}_{{\epsilon}_{i}}\right) \;\; ; \;\; q_{3}(\boldsymbol{v}_{\boldsymbol{f}}) = \prod\limits_{j=1}^{M} q_{3j}\!\left({v}_{{f}_{{j}}}\right),   $$

The approximate *q* (***f***,***v***_***ε***_,***v***_***f***_|***g***) is obtained by minimizing the Kullback-Leibler divergence, defined as:

(20) $${} \begin{aligned} &\text{KL}\left(q\!\left(\boldsymbol{f},\boldsymbol{v}_{\boldsymbol{\epsilon}},\boldsymbol{v}_{\boldsymbol{f}}|\boldsymbol{g}\right) :p\!\left(\boldsymbol{f},\boldsymbol{v}_{\boldsymbol{\epsilon}},\boldsymbol{v}_{\boldsymbol{f}}|\boldsymbol{g}\right)\right) \\&= \iint \ldots \int q\!\left(\boldsymbol{f},\boldsymbol{v}_{\boldsymbol{\epsilon}},\boldsymbol{v}_{\boldsymbol{f}}|\boldsymbol{g}\right)\; \ln \frac{q\!\left(\boldsymbol{f},\boldsymbol{v}_{\boldsymbol{\epsilon}},\boldsymbol{v}_{\boldsymbol{f}}|\boldsymbol{g}\right)} {p\!\left(\boldsymbol{f},\boldsymbol{v}_{\boldsymbol{\epsilon}},\boldsymbol{v}_{\boldsymbol{f}}|\boldsymbol{g}\right)} \, \text{d} \boldsymbol{f}\, \text{d} \boldsymbol{v}_{\boldsymbol{\epsilon}}\, \text{d} \boldsymbol{v}_{\boldsymbol{f}}, \end{aligned}   $$

where we also used the notations:

(21) $$ \text{d} \boldsymbol{v}_{\boldsymbol{\epsilon}} = \prod\limits_{i=1}^{N}\, \text{d} {v}_{{\epsilon}_{i}} \;\; ; \;\; \text{d} \boldsymbol{v}_{\boldsymbol{f}} = \prod\limits_{j=1}^{M} \text{d} {v}_{{f}_{j}}.  $$

Like in the MAP case, the minimization can be done via alternate optimization resulting in the following proportionalities:

(22) $${} {\fontsize{8.1pt}{11.8pt}{\begin{aligned} \left\{ \begin{array}{l} {} q_{1}\left(\,\boldsymbol{f}\right) \propto \exp \left\{ \left\langle \ln p\!\left(\,\boldsymbol{f},\boldsymbol{v}_{\boldsymbol{\epsilon}},\boldsymbol{v}_{\boldsymbol{f}}|\boldsymbol{g}\right) \right\rangle_{q_{2}(\boldsymbol{v}_{\boldsymbol{\epsilon}}) \; q_{3}\left(\boldsymbol{v}_{\boldsymbol{f}}\right)} \right\} \\ {} q_{2i}\!\left({v}_{{\epsilon}_{i}}\right) \propto \exp \left\{ \left\langle \ln p\!\left(\,\boldsymbol{f},\boldsymbol{v}_{\boldsymbol{\epsilon}},\boldsymbol{v}_{\boldsymbol{f}}|\boldsymbol{g}\right) \right\rangle_{q_{1}(\boldsymbol{f}) \; q_{2-i}\left(v_{{\epsilon}_{i}}\right) \; q_{3}(\boldsymbol{v}_{\boldsymbol{f}})} \right\}, \; i \in \left\{ 1,2 \ldots, N \right\}\\ {} q_{3j}\!\left({v}_{{f}_{j}}\right) \propto \exp \left\{ \left\langle \ln p\!\left(\,\boldsymbol{f},\boldsymbol{v}_{\boldsymbol{\epsilon}},\boldsymbol{v}_{\boldsymbol{f}}|\boldsymbol{g}\right) \right\rangle_{q_{1}(\boldsymbol{f}) \; q_{2}\left(\boldsymbol{v}_{\boldsymbol{\epsilon}}\right)\; q_{3-j}\!\left(v_{{f}_{j}}\right)} \right\}\!, \; j \in \left\{ 1,2 \ldots, M \right\} \end{array}\right. \end{aligned}}}   $$

where we used the notations:

(23) $${} {\fontsize{9.2pt}{9.6pt}{\begin{aligned} q_{2-i}\!\left({v}_{{\epsilon}_{i}}\right) &= \prod\limits_{k=1,k \neq i}^{N} q_{2k}\!\left({v}_{{\epsilon}_{k}}\right) \quad ; \quad q_{3-j}\!\left({v}_{{f}_{j}}\right) \\&= \prod\limits_{k=1,k \neq j}^{M} q_{3k}\!\left({v}_{{f}_{k}}\right) \quad ; \quad \left\langle u(x) \right\rangle_{v(y)} = \int u(x) v(y)\; \text{d} y. \end{aligned}}}  $$

From the proportionalities showed in Eq. (22), we derive the probability distributions corresponding to *q*_1_(***f***), $\phantom {\dot {i}\!}q_{2i}(v_{{\epsilon }_{i}})$, $\phantom {\dot {i}\!}q_{3j}(v_{{f}_{j}})$ and the corresponding parameters. The detailed computations are presented in Appendix 2. Here, we only present the general strategy: in the first step, developing the proportionality corresponding to *q*_1_(***f***), we obtain an expression of an exponential, having as argument a quadratic criterion, leading to the conclusion that *q*_1_(***f***) is a multivariate normal distribution. By minimizing the criterion, we obtain the analytical expression of the corresponding mean. The variance is obtained by identification. However, in this stage, both the analytical expressions of the mean and variance depend on expectancies corresponding to the two variances involved in the model, i.e., $\phantom {\dot {i}\!}v_{{\epsilon }_{i}}$ and $\phantom {\dot {i}\!}v_{{f}_{j}}$. In the second step, developing the proportionalities corresponding to $\phantom {\dot {i}\!}q_{2i}({v}_{{\epsilon }_{i}})$ and $\phantom {\dot {i}\!}q_{3j}({v}_{{f}_{j}}),$ we establish that they are both inverse gamma distributions. This is done using that the expectancies containing **f** can be handled because *q*_1_(**f**) was proved to be a multivariate Normal distribution in the previous step. Then, using the fact that $\phantom {\dot {i}\!}q_{2i}(v_{{\epsilon }_{i}})$ and $\phantom {\dot {i}\!}q_{3j}(v_{{f}_{j}})$ are inverse gamma distributions, the expectancies that appear in the expressions of the mean and variance corresponding to the multivariate normal distribution *q*_1_(***f***) can be computed. We establish analytical expressions for all the parameters of the distributions. The analytical expressions of the parameters are presented in the Eqs. (24a), (24b), and (24c).

(24a) $${} {\fontsize{8.4pt}{9.6pt}{\begin{aligned} q_{1}\left(\,\boldsymbol{f}\right) = \mathcal{N}\left(\boldsymbol{f} | \,{\widehat{\boldsymbol{f}}}_{\text{PM}}, {\widehat{\boldsymbol{\Sigma}}} \right), \left\{ \begin{array}{l} {\widehat{\boldsymbol{f}}}_{\text{PM}} = \left(\boldsymbol{H}^{T} {\widehat{\boldsymbol{V}_{\boldsymbol{\epsilon}}^{-1}}} \boldsymbol{H} + {\widehat{\boldsymbol{V}_{\boldsymbol{f}}^{-1}}}\right)^{-1} \boldsymbol{H}^{T} {\widehat{\boldsymbol{V}_{\boldsymbol{\epsilon}}^{-1}}} \boldsymbol{g} \\ {\widehat{\boldsymbol{\Sigma}}} = \left(\boldsymbol{H}^{T} {\widehat{\boldsymbol{V}_{\boldsymbol{\epsilon}}^{-1}}} \boldsymbol{H} + {\widehat{\boldsymbol{V}_{\boldsymbol{f}}^{-1}}} \right)^{-1} \end{array}\right. \end{aligned}}}   $$

(24b) $${} {\fontsize{7.6pt}{9.6pt}{\begin{aligned} q_{2i}\!\left(v_{{\epsilon}_{i}}\right) = \mathcal{I}\mathcal{G} \left(v_{{\epsilon}_{i}}|\alpha_{\epsilon_{i}},\beta_{\epsilon_{i}}\right), \left\{ \begin{array}{l} {\alpha_{\epsilon_{i}}} = \alpha_{\epsilon 0} + \frac{1}{2} \\ {\beta_{\epsilon_{i}}} = \beta_{\epsilon 0} + \frac{1}{2} \left[\boldsymbol{H}_{i} {\widehat{\boldsymbol{\Sigma}}} {\boldsymbol{H}_{i}^{T}} + \left({g}_{i} - \boldsymbol{H}_{i}\, {\widehat{\boldsymbol{f}}}_{\text{PM}}\right)^{2}\right] \end{array}\right. \end{aligned}}}   $$

(24c) $${} {\fontsize{9.8pt}{9.6pt}{\begin{aligned} q_{3j}\!\left({v}_{{f}_{j}}\right) = \mathcal{I}\mathcal{G} \left({v}_{{f}_{j}}|\alpha_{f_{j}},\beta_{f_{j}}\right), \left\{ \begin{array}{ll} \alpha_{f_{j}} = \alpha_{f 0} + \frac{1}{2} \\ \beta_{f_{j}} = \beta_{f 0} + \frac{1}{2} \left({\widehat{{f}_{j}}}_{\text{PM}}^{2} + {\widehat{\boldsymbol{\Sigma}}}_{jj} \right) \end{array}\right. \end{aligned}}}   $$

Equation 24a provides the dependency of the parameters corresponding to the multivariate normal distribution *q*_1_(***f***) and the others hyperparameters involved in the hierarchical model: the mean ${\widehat {\boldsymbol {f}}}_{\text {PM}}$ and the covariance matrix ${\widehat {\boldsymbol {\Sigma }}}$ depend on ${\widehat {\boldsymbol {V}_{\boldsymbol {\epsilon }}^{-1}}}$ and ${\widehat {\boldsymbol {V}_{\boldsymbol {f}}^{-1}}}$. Eq. (70) (in Appendix 2) defines ${\widehat {\boldsymbol {V}_{\boldsymbol {\epsilon }}^{-1}}}$ and ${\widehat {\boldsymbol {V}_{\boldsymbol {f}}^{-1}}}$ via $\left \{ \alpha _{\epsilon _{i}},\beta _{\epsilon _{i}}\right \}, i \in \left \{ 1, 2, \ldots, N \right \}$ and $\left \{ \alpha _{f_{j}},\beta _{f_{j}}\right \}, j \in \left \{ 1, 2, \ldots, M \right \}$. For the mean and the variance, we obtain the following dependency:


(25)


Equation 24b leads to the following dependency scheme: 
(26)


Equation 24c leads to the following dependency scheme: 
(27)


The dependencies presented in (25), (26), and (27) leads to an iterative algorithm with a parameter update for every step. The algorithm is as follows:

- Initialization
- Use Eqs. (24a) and (70) to compute ${\widehat {\boldsymbol {f}}}_{\text {PM}}, {\widehat {\boldsymbol {\Sigma }}}$
- Use Eq. (24b) to compute $\left \{\alpha _{\epsilon _{j}},\beta _{\epsilon _{j}}\right \}$ and ${\widehat {\boldsymbol {V}_{\boldsymbol {\epsilon }}^{-1}}}$
- Use Eq. (24c) to compute $\left \{\alpha _{f_{j}},\beta _{f_{j}}\right \}$ and ${\widehat {\boldsymbol {V}_{\boldsymbol {f}}^{-1}}}$

**Fig. 4:**
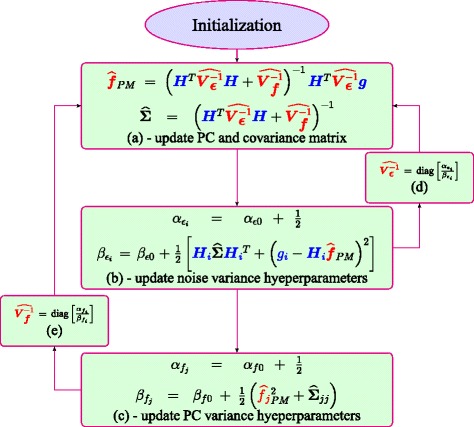
IGSM hierarchical model—PM via VBA estimation (partial separability): iterative algorithm

The iterative algorithm obtained using the PM estimation, via VBA partial separability, is presented in Fig. 4.

In order to initialize the algorithm, we define the matrices ${\widehat {\boldsymbol {V}_{\boldsymbol {\epsilon }}^{-1}}^{(0)}}$ and ${\widehat {\boldsymbol {V}_{\boldsymbol {f}}^{-1}}^{(0)}}$, corresponding to the iteration zero of the algorithm. For the first iteration, using those values of matrices, the algorithm updates the estimations corresponding to the PC vector and the corresponding covariance matrix (a). Except the two matrices used, the other terms involved in the equations are known: the recorded signal ***g*** and the matrix ***H***. After the PC vector and the covariance matrix are updated, they are used as terms in the equations updating the hyperparameters involved in the model. For updating the hyperparameters corresponding to the noise variances (b) and PC variances (c), the algorithm is using the estimation of the PC vector and the covariance matrix corresponding to the first iteration, obtained in (a). Then, the estimation corresponding to the noise variance (b) are used as input in (a), corresponding to the second iteration, via (d) and (e).

For initializing the algorithm, one of the possible choices is assigning values for the following parameters: $\left \{{\alpha _{f_{j}}^{(0)}}, {\beta _{f_{j}}^{(0)}}\right \}$, *j*∈{1,2,…,*M*} representing ${\widehat {\boldsymbol {V}_{\boldsymbol {f}}^{-1}}}^{(0)}$ and $\left \{\alpha _{\epsilon _{i}}^{(0)},\beta _{\epsilon _{i}}^{(0)}\right \}$, *i*∈{1,2,…,*N*} representing ${\widehat {\boldsymbol {V}_{\boldsymbol {\epsilon }}^{-1}}}^{(0)}$, corresponding to the step zero of the algorithm. This choice for the initialization procedure is sufficient, in the sense that the considered parameters from above represent all the necessary informations for starting the first iteration of the algorithm and computing all other parameters of the algorithm corresponding to step zero, i.e., ${\widehat {\boldsymbol {f}}}_{\text {PM}}^{(0)}$ and ${\widehat {\boldsymbol {\Sigma }}}^{(0)}$. For the parameters $\alpha _{\epsilon _{i}}^{(0)}$, $\beta _{\epsilon _{i}}^{(0)}$ and $\alpha _{f_{j}}^{(0)}$, $\beta _{f_{j}}^{(0)}$, we consider the following initialization:

(28) $$ \alpha_{\epsilon_{j}}^{(0)} = \alpha_{\epsilon 0} \;\;, \;\; \beta_{\epsilon_{j}}^{(0)} = \beta_{\epsilon 0} \;\;, \;\; \alpha_{f_{j}}^{(0)} = \alpha_{f 0} \;\;, \;\; \beta_{f_{j}}^{(0)} = \beta_{f 0}  $$

**Fig. 5:**
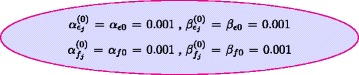
IGSM hierarchical model—PM via VBA estimation (partial separability): initialization

A natural choice in this case is Non-Informative Prior Law (NIPL). The inverse gamma distribution is weak for parameters *α*→0 and *β*→0, so one possible choice is $\alpha _{\epsilon _{j} 0} = \beta _{\epsilon _{j} 0} = 0.001$ and $\alpha _{f_{j} 0} = \beta _{f_{j} 0} = 0.001$. In particular, such an approach is consistent with a non-supervised algorithm. The considered initialization is presented in Fig. 5.

### Posterior mean (via VBA) IGSM (full separability)

In Subsection 4.2, the Student’s *t* model is considered and the PM estimator is used. The posterior law is approximated by a separable one, Eq. (18), where the notations for *q*_2_(***v***_***ε***_) and *q*_3_(***v***_***f***_), introduced in Eq. (19), represent a full separability relative to ***v***_***ε***_ and ***v***_***f***_. But the prior law *p* (***f***,***v***_***ε***_,***v***_***f***_|***g***) is not approximated by a fully separable one since for *q*_1_(***f***), we consider a multivariate law modeling the vector ***f***. In this subsection, we investigate the development of the proposed model and the same PM estimator, but the posterior law is approximated by a fully separable law relative to all the unknowns, i.e., also for ***f***. The interest of such development concerns the applications where the precision required is high, making the numerical computations very costly. In this case, the posterior law from the hierarchical model *p* (***f***,***v***_***ε***_,***v***_***f***_|***g***) is approximated by a fully separable probability density function:

(29) $${} {\fontsize{8.8pt}{9.6pt}{\begin{aligned} p\!\left(\boldsymbol{f},\boldsymbol{v}_{\boldsymbol{\epsilon}},\boldsymbol{v}_{\boldsymbol{f}}|\boldsymbol{g}\right) \approx q\!\left(\boldsymbol{f},\boldsymbol{v}_{\boldsymbol{\epsilon}},\boldsymbol{v}_{\boldsymbol{f}}|\boldsymbol{g}\right) &= \prod\limits_{j=1}^{M} q_{1j}\!\left({f}_{j}\right) \prod\limits_{i=1}^{N} q_{2i}\!\left({v}_{{\epsilon}_{i}}\right) \prod\limits_{j=1}^{M} q_{3j}\!\left(v_{f_{j}}\right)\\ &= q_{1}(\boldsymbol{f})\, q_{2}(\boldsymbol{v}_{\boldsymbol{\epsilon}})\, q_{3}(\boldsymbol{v}_{\boldsymbol{f}}) \end{aligned}}}  $$

where we used the notation introduced in (19) and also the following notations, considered during this paragraph:

(30) $${} {\fontsize{8.6pt}{9.6pt}{\begin{aligned} q_{1}\left(\,\boldsymbol{f}\right) = \prod\limits_{j=1}^{M} q_{1j}\!\left(\,{f}_{j}\right)\; ; \; \text{d}\, \boldsymbol{f} = \prod\limits_{j=1}^{M} q_{1j}\!\left(\,{f}_{j}\right)\; ; \; q_{1-j}\!\left(\,{f}_{j}\right) = \prod\limits_{k=1,k\neq j}^{M} q_{1k}\!\left(\,{f}_{k}\right) \end{aligned}}}   $$

Like in Subsection 4.2, the law *q* (***f***,***v***_***ε***_,***v***_***f***_|***g***) is obtained by minimizing the Kullback-Leibler divergence, Eq. (20), via alternate optimization, obtaining the proportionalities presented in Eq. (31):

(31) $${} {\fontsize{7.8pt}{10.6pt}{\begin{aligned} \left\{ \begin{array}{l} q_{1j}\!\left(\,{f}_{j}\right) \;\,\propto \; \exp \left\{\left\langle \ln p\!\left(\boldsymbol{f},\boldsymbol{v}_{\boldsymbol{\epsilon}},\boldsymbol{v}_{\boldsymbol{f}}|\boldsymbol{g}\right) \right\rangle_{q_{1-j}(f_{j})\; q_{2}\!\left(\boldsymbol{v}_{\boldsymbol{\epsilon}}\right) \; q_{3}\!\left(\boldsymbol{v}_{\boldsymbol{f}}\right)}\right\}\;\;\; j \in \left\{1,2 \ldots, M \right\} \\ q_{2i}\!\left({v}_{{\epsilon}_{i}}\right) \propto \exp \left\{\left\langle \ln p\!\left(\boldsymbol{f},\boldsymbol{v}_{\boldsymbol{\epsilon}},\boldsymbol{v}_{\boldsymbol{f}}|\boldsymbol{g}\right) \right\rangle_{q_{1}(\boldsymbol{f}) \; q_{2-i}\!\left(v_{{\epsilon}_{i}}\right) \; q_{3}\!\left(\boldsymbol{v}_{\boldsymbol{f}}\right)}\right\}, \;\; i \in \left\{1,2 \ldots, N \right\} \\ q_{3j}\!\left({v}_{{f}_{j}}\right) \propto \exp \left\{\left\langle \ln p\!\left(\boldsymbol{f},\boldsymbol{v}_{\boldsymbol{\epsilon}},\boldsymbol{v}_{\boldsymbol{f}}|\boldsymbol{g}\right) \right\rangle_{q_{1}(\boldsymbol{f}) \; q_{2}\!\left(\boldsymbol{v}_{\boldsymbol{\epsilon}}\right) \; q_{3-j}\!\left(v_{f_{j}}\right)}\right\}, \;\; j \in \left\{1,2 \ldots, M \right\} \end{array}\right. \end{aligned}}}  $$

**Fig. 6:**
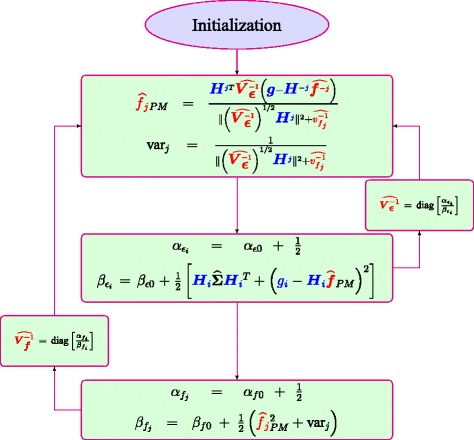
IGSM hierarchical model—PM via VBA estimation (full separability): iterative algorithm

The detailed computations are presented in Appendix 3. The analytical expressions of the parameters are presented in Eqs. (32a), (32b), and (32c).

(32a) $${} q_{1}\!\left({f}_{j}\right) = \mathcal{N} \left({f}_{j} | \,{\widehat{{f}_{j}}}_{\text{PM}}, \text{var}_{j} \right), \left\{ \begin{array}{l} {\widehat{{f}_{j}}}_{\text{PM}} = \frac{\boldsymbol{H}^{{j} T} {\widehat{\boldsymbol{V}_{\boldsymbol{\epsilon}}^{-1}}} \left(\boldsymbol{g} - \boldsymbol{H}^{-{j}} {\widehat{\boldsymbol{f}^{-j}}}\right)}{\|\left({\widehat{\boldsymbol{V}_{\boldsymbol{\epsilon}}^{-1}}}\right)^{1/2} \boldsymbol{H}^{j}\|^{2} + \,{\widehat{v_{f_{j}}^{-1}}}} \\ \text{var}_{j} = \frac{1}{\| \left({\widehat{\boldsymbol{V}_{\boldsymbol{\epsilon}}^{-1}}}\right)^{1/2} \boldsymbol{H}^{j}\|^{2} + \,{\widehat{v_{f_{j}}^{-1}}}} \end{array}\right.   $$

(32b) $${} q_{2i}\!\left(v_{{\epsilon}_{i}} \right) = \mathcal{I}\mathcal{G} \left(v_{{\epsilon}_{i}}|\alpha_{\epsilon_{i}},\beta_{\epsilon_{i}}\right), \left\{ \begin{array}{l} \alpha_{\epsilon_{i}} = \alpha_{\epsilon 0} + \frac{1}{2} \\ \beta_{\epsilon_{i}} = \beta_{\epsilon 0} + \frac{1}{2} \left({g}_{i} - \boldsymbol{H}_{i}\,{\widehat{\boldsymbol{f}}}_{\text{PM}}\right) \end{array}\right.   $$

(32c) $${} q_{3j}\!\left({v}_{{f}_{j}}\right) = \mathcal{I}\mathcal{G} \left({v}_{{f}_{j}}|\alpha_{f_{j}},\beta_{f_{j}}\right), \left\{ \begin{array}{l} \alpha_{f_{j}} = \alpha_{f 0} + \frac{1}{2} \\ \beta_{f_{j}} = \beta_{f 0} + \frac{1}{2} \left({\widehat{{f}_{j}}}_{\text{PM}}^{2} + \text{var}_{j} \right), \end{array}\right.   $$

where ***H*** ^*j*^ represents the column *j* of the matrix ***H***, ***H*** ^−*j*^ represents the matrix ***H*** except the column *j*, and ***f*** ^−*j*^ represents the vector ***f*** except the element *f*_*j*_. The iterative algorithm is presented in Fig. 6. The initialization is done in the same conditions as in the partial separability case (Fig. 5).

## Simulations

This section presents the simulations corresponding to synthetic and real data. For synthetic data, we compare five algorithms: joint MAP with Gaussian prior, posterior mean with Gaussian prior, joint MAP with IGSM prior, posterior mean (via VBA) with IGSM prior (partial separability), and posterior mean (via VBA) with IGSM prior (full separability). For each iterative algorithm, we present a comparison between the algorithm’s estimation and the synthetic data, i.e., a comparison between $\widehat {\boldsymbol {f}}_{\text {Method}}$ and ***f***, between $\widehat {\boldsymbol {g}}_{\text {Method}}$ and ***g*** and between $\widehat {\boldsymbol {g}}_{\text {Method}}$ and ***g***_0_ theoretical signal (***g*** without noise). For every algorithm considered, we present the convergency analysis of the parameters and hyperparameters involved. Then, we present a comparison between the estimations of proposed algorithms and the classical *FFT* method. Finally, the proposed algorithms are tested 10 times over the same data, but different noise realization, in order to obtain the *L*_2_ error vector (the normalized difference between data and estimated data, considered for ***f***, ***g***, and theoretical signal ***g***_0_) and compare the performances of each algorithm. These comparisons between error vectors corresponding to each algorithm are presented at the end of the subsection. For the synthetic data, we consider the following protocol: we consider a theoretical PC vector ***f*** and the corresponding theoretical signal ***H*** ***f*** and we consider the corresponding signal ***g***=***H*** ***f***+***ε***, by adding noise over the theoretical signal. In this article, we consider for the synthetic case three different levels of noise: 15, 10, and 5 dB. In this section, we include only the detailed simulations for the 5-dB case. The other two cases are presented in the Additional file 1. The considered signal represents a 4-day signal, sampled every hour. The matrix ***H*** considered in this set of simulations is a cosine plus sine matrix.

### Synthetic data 05 dB

For testing, we have considered a 4-day signal, corresponding to a sparse PC vector, having non-zero values for 11, 15, and 23 h. We consider this particular structure for the following reason: we want to verify if the proposed method can precisely distinguish the peaks inside the circadian domain. As we have mentioned, for such signals, via the FFT, we obtain a high peak corresponding to 24 h and the corresponding harmonics, but this method offers no information for certain values in the circadian domain. We have showed in Section 2 (Fig. 1) that a dominant period, corresponding to 23 h, is wrongly estimated at 24 h via FFT method and offers no other informations in the interval [20–31].

#### Data 05 dB

**Fig. 7:**
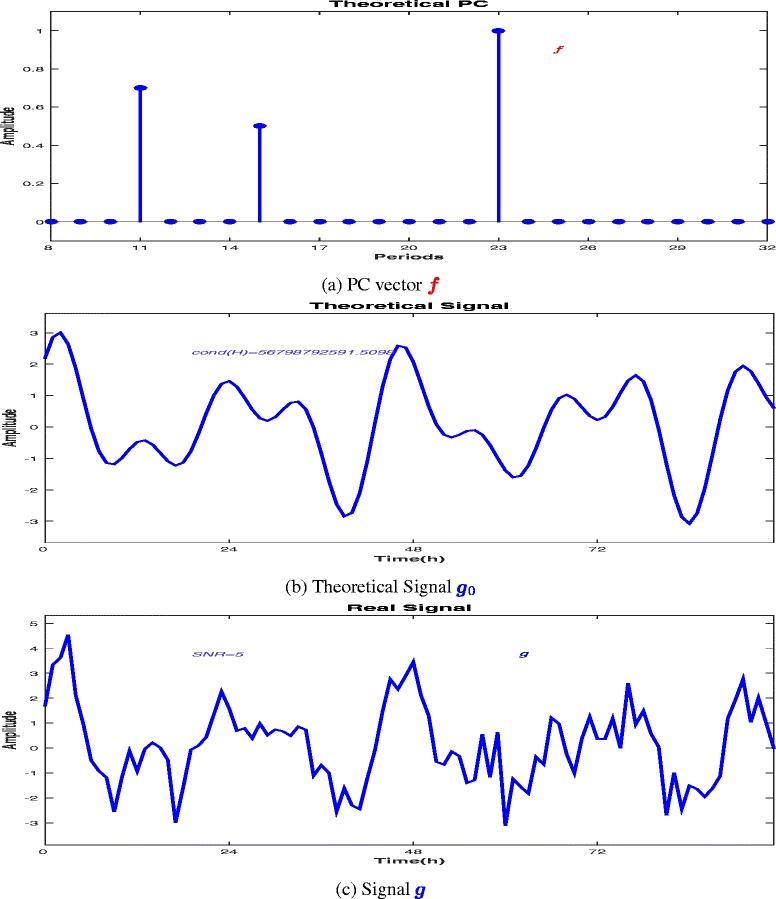
***f*** PC vector, theoretical signal ***g***_0_, and input signal ***g***=***g***_0_+***ε*** of the model (5 dB). **a** PC vector ***f***. **b** Theoretical signal ***g***_0_. **c** Signal ***g***

The PC vector ***f***, theoretical signal ***g***_0_, and the signal ***g*** are presented in Fig. 7.

Figure 7 a shows the theoretical PC, having the non-zero periods corresponding to 11, 15, and 23 h. All the other values in the PC vector are zero. Figure 7 b presents the signal corresponding to the linear model considered in Eq. (3), neglecting the errors, ***g***_0_=***H*** ***f***. We note that the conditioning number of the matrix ***H*** is cond(***H***)=56,798,792,591. All the simulations are done using the input as the noisy signal ***g*** corresponding to the linear model, Eq. (3), presented in Fig. 7 c. We compare the estimated PC vector with the theoretical one (Fig. 7 a) and the corresponding reconstructed signal with ***g***_0_ and ***g***. The comparison with the theoretical signal ***g***_0_ is important in order to verify if the propose algorithm can distinguish the peaks corresponding to the biological phenomena from the ones corresponding to the noise.

#### JMAP IGSM 05 dB

**Fig. 8:**
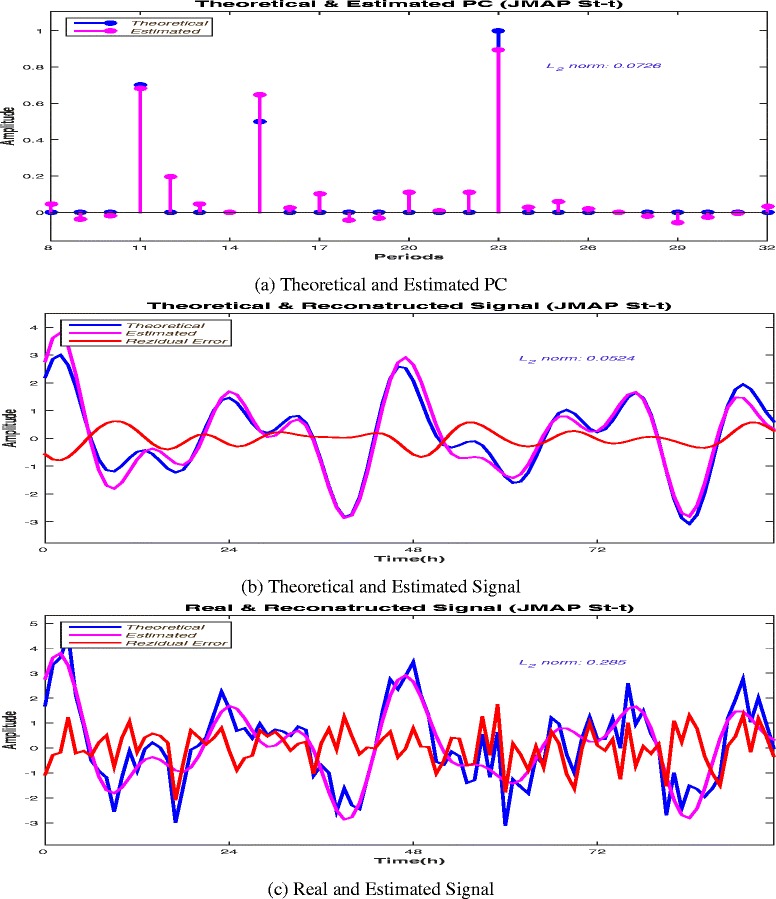
JMAP IGSM estimation (5 dB). **a** Theoretical and estimated PC. **b** Theoretical and estimated signal. **c** Real and estimated signal

A comparison between the synthetic data and the JMAP estimation, corresponding to the IGSM prior hierarchical model is presented in Fig. 8. We compare the theoretical PC vector ***f*** and the JMAP estimation $\widehat {\boldsymbol {f}}_{\text {JMAP}}$. We also present the comparison between the estimated $\widehat {\boldsymbol {g}}_{\text {JMAP}}$ and ***g*** and the comparison between the estimated $\widehat {\boldsymbol {g}}_{\text {JMAP}}$ and the theoretical signal (without noise) ***g***_0_.

The proposed method is searching for a sparse solution corresponding to the linear model, Eq. (3). The comparison between the theoretical signal ***g***_0_ and ${\widehat {\boldsymbol {g}}}_{\text {JMAP}}$ (Fig. 8 b) shows that the proposed algorithm is converging to a solution that leads to a fairly accurate reconstruction, having the *L*_2_ norm error $\delta \boldsymbol {g}_{0} = \frac {\|\boldsymbol {g}_{0}-{\widehat {\boldsymbol {g}}}_{\textit {JMAP}}\|_{2}^{2}}{\|\boldsymbol {g}_{0}\|_{2}^{2}}=0.0524$. For the PC vector, the reconstruction error is $\delta \boldsymbol {f} = \frac {\|\boldsymbol {f}-\widehat {\boldsymbol {f}}_{\textit {JMAP}}\|_{2}^{2}}{\|\boldsymbol {f}\|_{2}^{2}} = 0.0726$. For the JMAP estimation, the condition imposed for the searched solution, i.e., the sparsity is not respected (Fig. 8 a). In fact, the alternate optimization algorithm considered for searching the JMAP solution is converging to a local minimum and the estimation errors corresponding to the JMAP estimation might be far from the example presented.

**Fig. 9:**
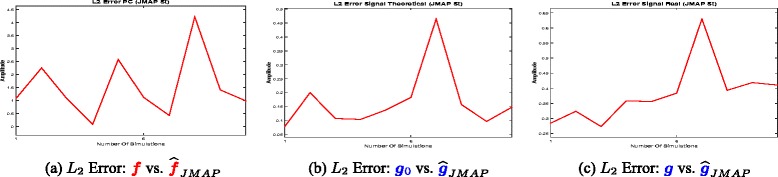
JMAP IGSM *L*_2_ error measured for 10 different noise realizations (5 dB). **a** *L*_2_ error: ***f*** vs. $\widehat {\boldsymbol {f}}_{\text {JMAP}}$. **b** *L*_2_ error: ***g***_0_ vs. $\widehat {\boldsymbol {g}}_{\text {JMAP}}$. **c** *L*_2_ error: ***g*** vs. $\widehat {\boldsymbol {g}}_{\text {JMAP}}$

Figure 9 a presents the variation of *L*_2_ PC vector error reconstruction for 10 different noise realization. As mentioned, the JMAP solution given by the alternate optimization algorithm is converging to a local minimum and the estimation may be very inaccurate. We note that the figure presents a variation of *L*_2_ PC vector error reconstruction from 0.0524 to 4.2841. Important variations corresponding to the *L*_2_ error reconstruction for the theoretical signal ***g***_0_ and signal ***g*** are presented in Fig. 9 b, c.

#### PM (via VBA, partial separability) IGSM 05 dB

**Fig. 10:**
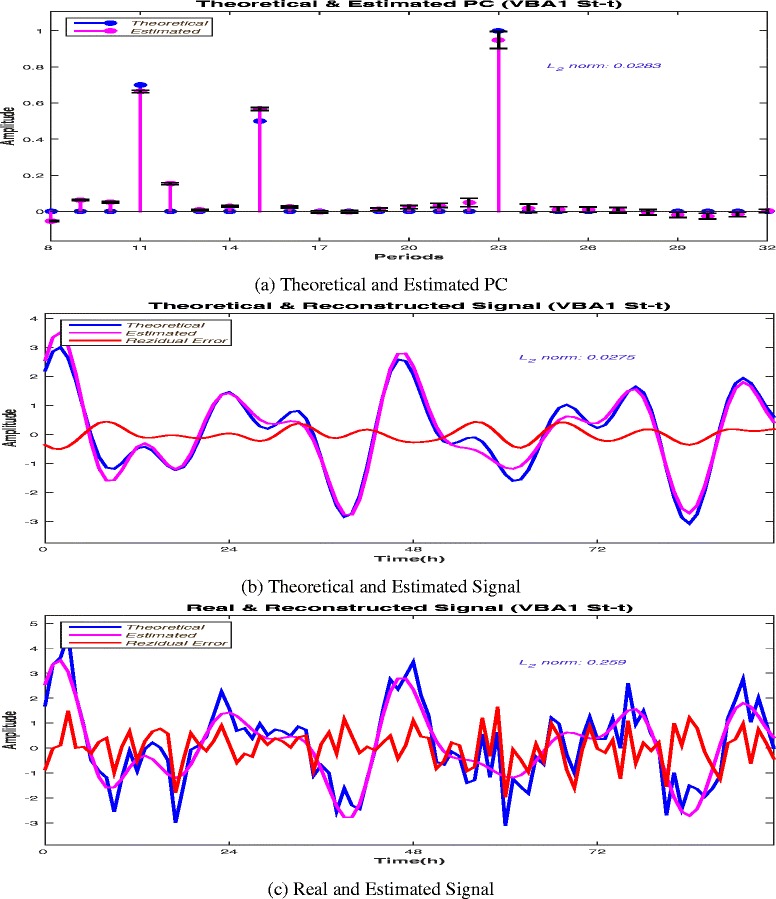
PM (via VBA, partial separability) IGSM estimation (5 dB). **a** Theoretical and estimated PC. **b** Theoretical and estimated signal. **c** Real and estimated signal

A comparison between the synthetic data and the PM (via VBA, partial separability) IGSM estimation is presented in Fig. 10. We compare the theoretical PC vector ***f*** with the PM (via VBA, partial separability) IGSM estimation $\widehat {\boldsymbol {f}}_{\text {PM}}$ (Fig. 10 a) and the corresponding reconstructed signal $\widehat {\boldsymbol {g}}_{\text {PM}}$ both with the theoretical signal ***g***_0_ (Fig. 10 b) and the input signal ***g*** (Fig. 10 c).

In the case of the posterior mean estimation via VBA, both the PC estimation and theoretical signal ***g***_0_ reconstruction are very accurate (Fig. 10 a, b). For the reconstruction of the theoretical signal ***g***_0_, the *L*_2_ error norm is $\delta \boldsymbol {g}_{0} = \frac {\|\boldsymbol {g}_{0}-\widehat {\boldsymbol {g}}_{\textit {PM}}\|_{2}^{2}}{\|\boldsymbol {g}_{0}\|_{2}^{2}}=0.0275$. For the PC vector, the reconstruction error is $\delta \boldsymbol {f} = \frac {\|\boldsymbol {f}-\widehat {\boldsymbol {f}}_{\textit {PM}}\|_{2}^{2}}{\|\boldsymbol {f}\|_{2}^{2}} = 0.0283$. The algorithm is converging to a sparse solution where all the non-zero peaks are detected. The residual error computed between ***g*** and the reconstructed signal is consistent with the error considered in the model, 5 dB (Fig. 10 c). During the algorithm, both inverse gamma shape parameters are constant (Eqs. (24b) and (24c)).

**Fig. 11:**
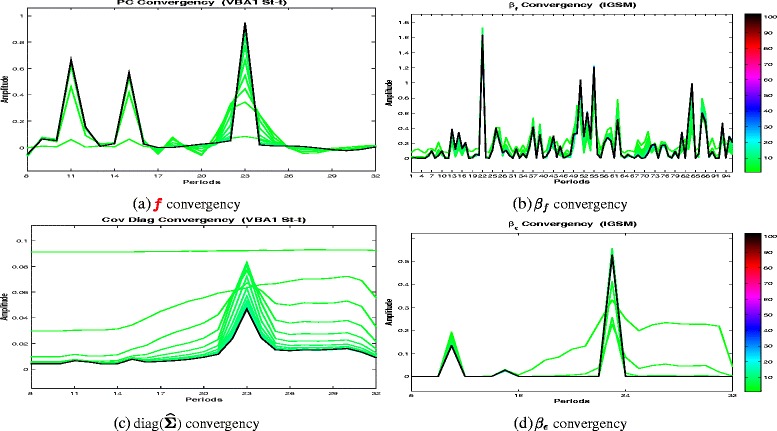
PM (via VBA, partial separability) IGSM hyperparameters and ***f*** convergency. **a** ***f*** convergency. **b** *β*_*f*_ convergency. **c** diag(${\widehat {\boldsymbol {\Sigma }}}$) convergency. **d** *β*_*ε*_ convergency

We present the convergence of the scale parameters *β*_*ε*_ and *β*_*f*_, (Fig. 11 b, d), the convergence of ***Σ*** covariance matrix diagonal (Fig. 11 c), and the convergence of the algorithm’s solution ***f***. For a better visualization of the PC convergence, ***f*** is plotted as a vector (Fig. 11 a). The color scale corresponding to each figure represents the iterations, showing a very fast convergence both for the parameters and hyperparameters involved in the model. All the estimations of the parameters and hyperparameters are superposed after the first ten iterations. In the previous paragraph, we have showed that the JMAP estimation for the proposed model is presenting high variations in terms of the error estimation and reconstruction. We show that for the PM estimation, the error variation is very small.

**Fig. 12:**
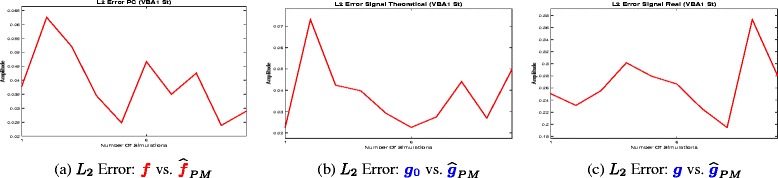
PM (via VBA, partial separability) IGSM *L*_2_ error measured for 10 different noise realizations (5 dB). **a** *L*_2_ error: ***f*** vs. $\widehat {\boldsymbol {f}}_{\text {PM}}$. **b** *L*_2_ error: ***g***_0_ vs. $\widehat {\boldsymbol {g}}_{\text {PM}}$. **c** *L*_2_ error: ***g*** vs. $\widehat {\boldsymbol {g}}_{\text {PM}}$

Figure 12 a presents the variation of *L*_2_ PC vector error reconstruction for 10 different noise realization. The figure presents a very small variation of *L*_2_ PC vector error reconstruction, between 0.02215 and 0.0621. Very small variations corresponding to the *L*_2_ error reconstruction for the theoretical signal ***g***_0_ and signal ***g*** are presented in Fig. 10 b, c.

#### PM (via VBA, full separability) IGSM 05 dB

**Fig. 13:**
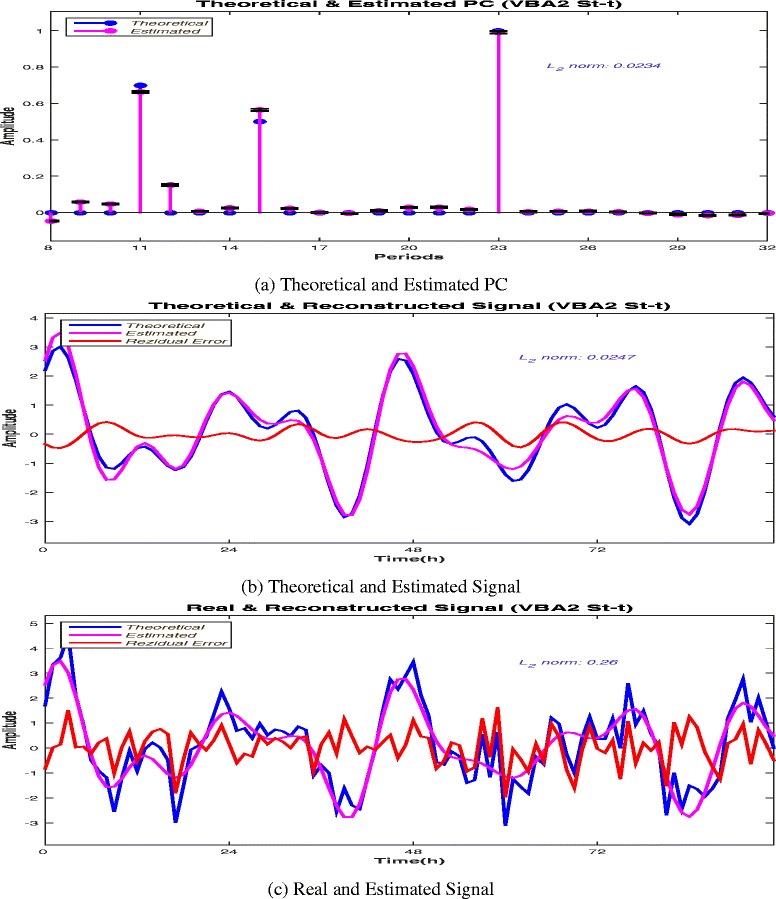
PM (via VBA, full separability) IGSM estimation (5 dB). **a** Theoretical and estimated PC. **b** Theoretical and estimated signal. **c** Real and estimated signal

The estimations for the full separability case are also accurate (Fig. 13).

Numerically, for the reconstruction of the theoretical signal ***g***_0_, the *L*_2_ error norm is $\delta \boldsymbol {g}_{0} = \frac {\|\boldsymbol {g}_{0}-\widehat {\boldsymbol {g}}_{\text {PM}}\|_{2}^{2}}{\|\boldsymbol {g}_{0}\|_{2}^{2}} = 0.0247$. For the PC vector, the reconstruction error is $\delta \boldsymbol {f} = \frac {\|\boldsymbol {f}-\widehat {\boldsymbol {f}}_{\text {PM}}\|_{2}^{2}}{\|\boldsymbol {f}\|_{2}^{2}} = 0.0234$.

**Fig. 14:**
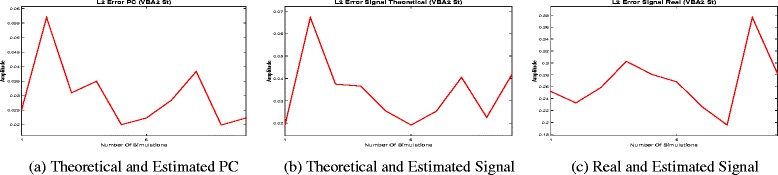
PM (via VBA, full separability) IGSM *L*_2_ error measured for 10 different noise realizations (5 dB). **a** Theoretical and estimated PC. **b** Theoretical and estimated signal. **c** Real and estimated signal

Figure 14 a presents the variation of *L*_2_ PC vector error reconstruction for 10 different noise realization. The figure presents a very small variation of *L*_2_ PC vector error reconstruction, between 0.02 and 0.067. Very small variations corresponding to the *L*_2_ error reconstruction for the theoretical signal ***g***_0_ and signal ***g*** are presented in Fig. 14 b, c.

#### Methods comparison 05 dB

**Fig. 15:**
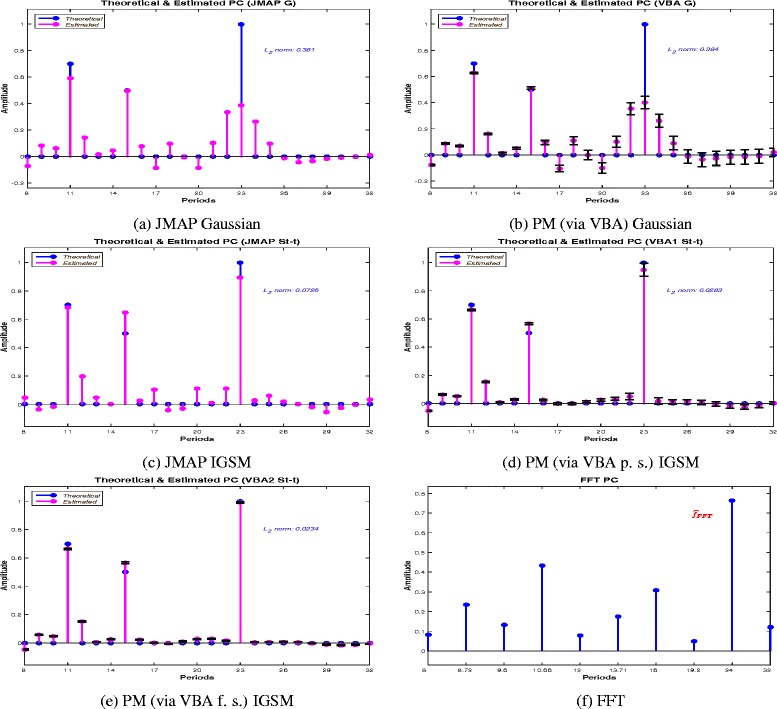
Method comparison (5 dB). **a** JMAP Gaussian. **b** PM (via VBA) Gaussian. **c** JMAP IGSM. **d** PM (via VBA p. s.) IGSM. **e** PM (via VBA f. s.) IGSM. **f** FFT

A comparison between the estimations corresponding to the IGSM proposed model is presented in Fig. 15 c (JMAP estimator), d (PM via VBA, partial separability estimator), and e (PM via VBA, full separability estimator). As mentioned in Section 3, during this article, we adopted a Bayesian approach. However, other approaches are possible, via regularization. For this reason, we include a comparison with the Gaussian case (i.e., Gaussian prior), via the two estimators discussed, Fig. 15 a (Gaussian model, JMAP estimator) and Fig. 15 b (PM via VBA estimator). A comparison with the FFT is presented in Fig. 15 f.

The *L*_2_ estimation error for the PC vector is very high for the two Gaussian models. Also, the estimations are not sparse. For the IGSM models, the JMAP estimator is providing a good estimation, but it is unstable. PM via VBA estimation, both partial and fully separable, provides very accurate stable estimations.

#### Error comparison 05 dB

**Fig. 16:**
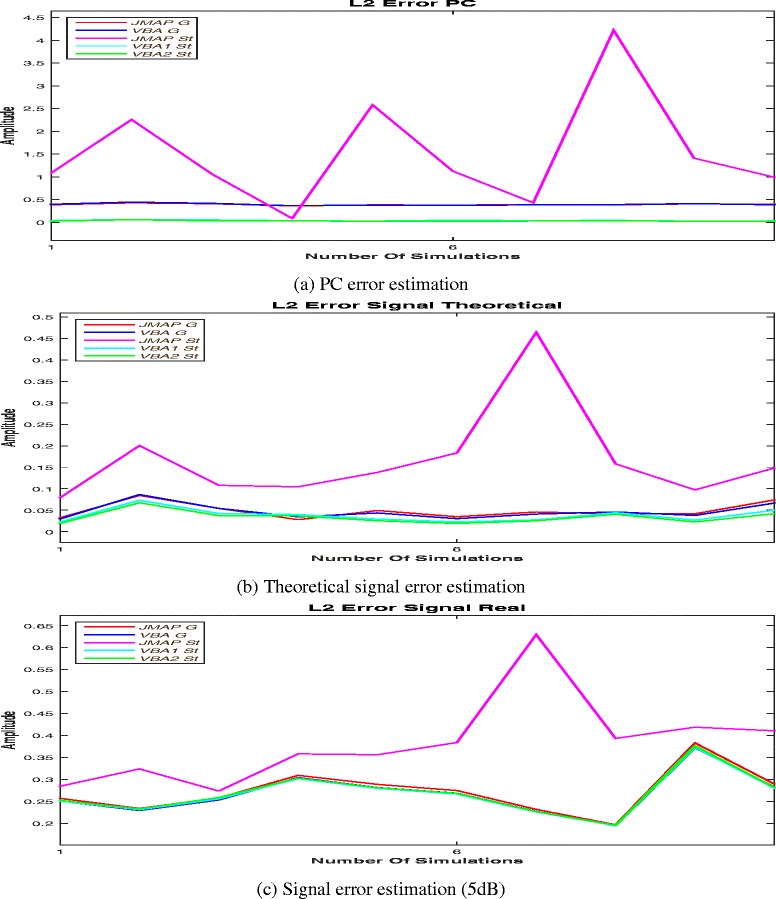
*L*_2_ errors estimation (5 dB). **a** PC error estimation. **b** Theoretical signal error estimation. **c** Signal error estimation (5 dB)

The *L*_2_ error measurement corresponding to the PC estimation, theoretical signal estimation, and signal estimation, for 10 different noise realization, is presented in the Fig. 16.

The *L*2 error corresponds to the PM via the VBA IGSM model corresponding to the PC vector estimation; Fig. 16 a shows the performances of the proposed algorithm compared to the Gaussian model and the JMAP estimation for IGSM model.

### Real data

**Fig. 17:**
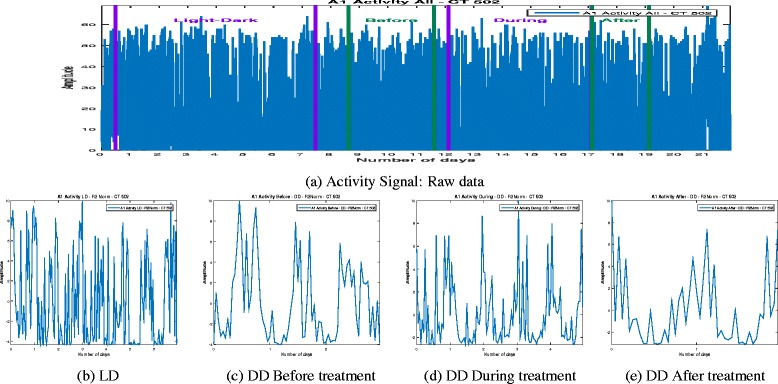
Activity raw data **a** and the corresponding parts **b**–**e** normalized and 1-hour sampled

This subsection is dedicated to the results corresponding to the real data, obtained in the experiments in chronobiology for cancer treatment. The particular experiment presented is realized on mice, investigating the tumor clock gene expression and the locomotor activity (rest-activity patterns) of KI/KI Per2::luc mouse, aged 10 weeks, singly housed in RT-BIO and synchronized with LD 12:12 (i.e., 12 h of light, followed by 12 h of darkness). The signal considered in this section is representing the locomotor activity of the mouse, which is known to be rhythmic. After the LD part of the signal, the mouse is kept in total darkness (DD) for 3 days, corresponding to the before-treatment part of the signal and then D-luciferin is loaded in subcutaneous implanted Alzet pump [90 mg/ml], recording for 5 days the signal corresponding to the during-treatment part of the signal. The last 2 days represent the after-treatment part of the signal. During the DD segment, the locomotor activity might be perturbed, due to the absence of the light-day regime and due to the treatment effects. Fig. 17 a presents the raw data corresponding to the locomotor activity signal. The four segments of interest are indicated in the figure. The raw data signal was sampled every minute. The stability of the period during all four segments is verified using the classical FFT method and the proposed algorithm, PM via VBA, partial separability. For the segments corresponding to the LD and during treatment, we have considered the moving window strategy, i.e., we have considered 4-day-length signals shifted every day in order to verify the stability or the variability of the dominant period. For the four segments studied, we consider the mean-zero signals, normalized between [ −10 : 10] and sampled every hour: the LD segment (Fig. 17 b), the DD-before segment (Fig. 17 c), the DD-during segment (Fig. 17 d), and the DD-after segment (Fig. 17 e).

For the LD segment, 7 days are available. We compute the PC corresponding to the signal using the proposed method and also using the FFT.

**Fig. 18:**
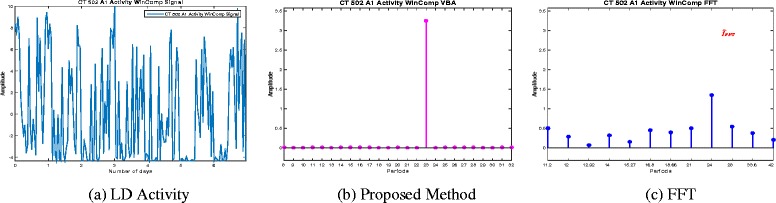
Considered signal (**a**) and the corresponding PC via VBA (**b**) and FFT (**c**) PC vector estimation

Via the FFT method, the dominant period is estimated at 24 h (Fig. 18 c). Evidently, beside the incertitude associated with the FFT-estimated PC vector, the existence of other rhythms cannot be established, being difficult to interpret all the peaks that appear PC vector. Via the proposed method, the estimated PC vector is a sparse one and the dominant period is estimated at 23 h (Fig. 18 b). We note that via the proposed method, there is no uncertainty concerning the biological phenomena. We consider 4-day-length signals (windows) from the available signal, with a shift of 1 day and compute the PC via FFT and the proposed method.

**Fig. 19:**
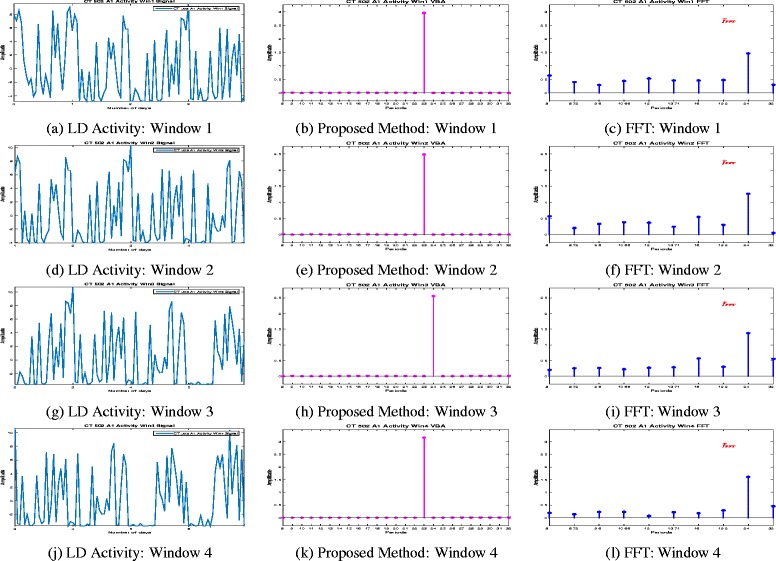
PC stability: PC estimation via FFT and VBA for 4-day-length signals. **a** LD activity: window 1. **b** Proposed method: window 1. **c** FFT: window 1. **d** LD activity: window 2. **e** Proposed method: window 2. **f** FFT: window 2. **g** LD activity: window 3. **h** Proposed method: window 3. **i** FFT: window 3. **j** LD activity: window 4. **k** Proposed method: window 4. **l** FFT: window 4

**Fig. 20:**
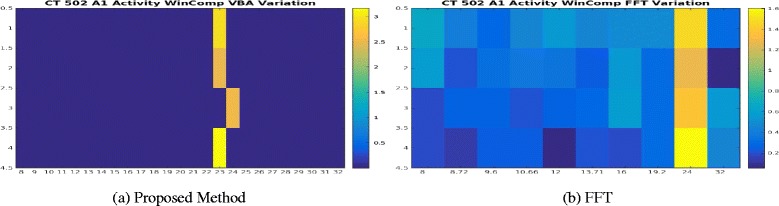
PC stability: proposed method (**a**) vs. FFT (**b**)

The four windows considered are presented in Fig. 19 a, d, g, j, and all four windows present a 24-h dominant period, via the FFT estimation (Fig. 19 c, f, i, l). Via the proposed method, we obtain sparse PC vectors, showing a variability of the dominant period, between 23 and 24 h. A comparison between the proposed method and the FFT method is presented in Fig. 20, showing the stability of the dominant period established by the FFT method (Fig. 20 b) and the variability established by the proposed method (Fig. 20 a) (the *x*-axis represents the periods inside the circadian domain and the *y*-axis represents the windows).

**Fig. 21:**
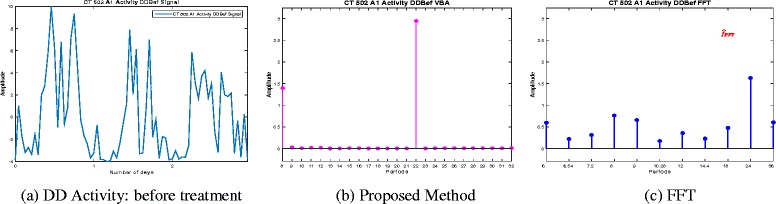
DD before treatment signal (**a**) and the corresponding PC via VBA (**b**) and FFT (**c**)

For the DD period, we consider first the before-treatment segment. A 3-day-length signal is available. The estimate PC vector, corresponding to the proposed method and FFT method is presented in Fig. 21.

Via the FFT, the highest peak is set at 24 h and the next highest peak is set at 8 h. Given the short length of the signal, 3 days, and the limitations of the FFT method, all the values inside the interval (18, 36) except 24 are not present in the estimated vector, so the values are uncertain. Via the proposed method, the dominant period is set at 22 h. For the during-treatment part of the data, a 5-day-length signal is available.

**Fig. 22:**
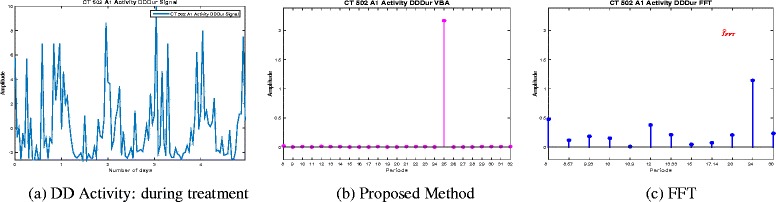
DD during treatment signal (**a**) and the corresponding PC via VBA (**b**) and FFT (**c**)

**Fig. 23:**
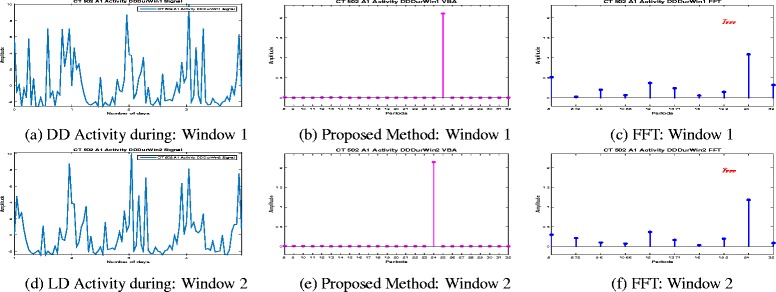
PC stability: PC estimation via FFT and VBA for 4-day-length signals, activity DD, during. **a** DD activity during: window 1. **b** Proposed method: window 1. **c** FFT: window 1. **d** LD activity during: window 2. **e** Proposed method: window 2. **f** FFT: window 2

**Fig. 24:**
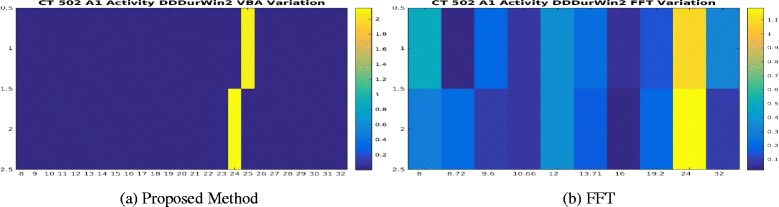
PC stability: proposed method (**a**) vs. FFT (**b**)

Via the proposed method, the estimated PC vector is a sparse vector, in accordance with the model, and the dominant period is estimated at 25 h (Fig. 22 b). For the FFT-estimated PC vector, the dominant period is set at 24 h (Fig. 22 c). Considering 4-day-length signals, we analyse the stability of the dominant period. Figures 23 and 24 show a stability of the dominant period established by the FFT method and a variability of the dominant period established by the proposed method.

**Fig. 25:**
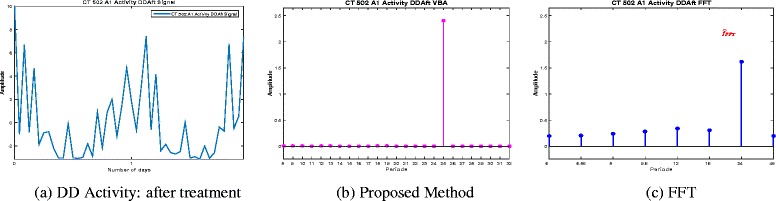
DD after treatment signal (**a**) and the corresponding PC via VBA (**b**) and FFT (**c**)

For the after treatment part, only a 2-day-length signal is available. The FFT method is establishing a 24-h dominant period (Fig. 25 c) while via the proposed method, the PC vector contains only one period, at 25 h (Fig. 25 b).

## Conclusions

In this article, we have proposed a new method for a precise estimation of the PC vector for biomedical signals, based on the general Bayesian inference and using a hierarchical model with sparsity enforcing prior. The prior considered was a Student’s *t* distribution expressed as the marginal of an infinite Gaussian scale mixture. The context of our work were the short signals relative to the prior knowledge for the dominant period (4-day signals and 24-h period). In Subsection 5.2, we applied the proposed method also for 2- and 3-day-length signals. The objective was to develop a method that can improve the precision given by the FFT method and also to account for the possible effects of the measurement errors and the uncertainties. The method was tested first on synthetic data, in order to be validated. The algorithms corresponding to the Gaussian model (JMAP and PM estimators) fail to accurately reconstruct the sparse theoretical PC vector. When using the JMAP estimator for the IGSM hierarchical model, the estimation is unstable. The error vectors corresponding to the JMAP-IGSM estimation (Fig. 16 a, b) are showing the drawbacks of the method. Both PM-IGSM models accurately estimate the theoretical PC vector, (Fig. 10 a, SNR =05 dB). The comparison between the reconstructed signal and the theoretical input (Fig. 10 b, SNR =05 dB) and the comparison between the reconstructed signal and the noised input (Fig. 10 c, SNR =05 dB) show a good reconstruction and a good residual error, consistent with the considered added noise for the noised signal ***g***. These algorithms allow the estimation of the covariance matrix. The convergence of ***f*** and hyperparameters is showing a fast convergence of the PM algorithms. The proposed method, PM via VBA, IGSM model was validated for a different set of data, at different ratios of noise, and the estimate was accurate in all the cases. For the real data, a comparison between the outputs is impossible. We have presented a comparison between the PC estimate corresponding to the PM-IGSM algorithm and the FFT estimate. The proposed method offers more precision compared to the FFT and is able to select the peaks corresponding to the biological phenomena. Via the proposed method, the conclusion imposed by the FFT method that the considered experiment presents a stability of the dominant period at 24 h is invalidated, showing a variation of the dominant period between 22 and 25 h.

## Appendices

### Appendix 1

#### Computations for JMAP estimation

This section presents the computation for the joint MAP estimation (Subsection 4.1). The estimation is done via alternate optimization. The criterion is $\mathcal {L}\!\left (\boldsymbol {f},\boldsymbol {v}_{\boldsymbol {\epsilon }},\boldsymbol {v}_{\boldsymbol {f}}\right) = -\ln p\!\left (\boldsymbol {f},\boldsymbol {v}_{\boldsymbol {\epsilon }},\boldsymbol {v}_{\boldsymbol {f}}|\boldsymbol {g}\right)$, and *p* (***f***,***v***_***ε***_,***v***_***f***_|***g***) is defined in Eq. (15). ∙ With respect to ***f***:

$${} {\fontsize{8.4pt}{9.6pt}{\begin{aligned} \frac{\partial \mathcal{L}\left(\boldsymbol{f}, \; {\widehat{\boldsymbol{v}_{\boldsymbol{\epsilon}}}}, \; {\widehat{\boldsymbol{v}_{\boldsymbol{f}}}} \right)}{\partial \boldsymbol{f}} = 0 & \Leftrightarrow \frac{\partial}{\partial \boldsymbol{f}} \left(\!\| \boldsymbol{V}_{\boldsymbol{\epsilon}}^{-\frac{1}{2}}\! \left(\boldsymbol{g} \,-\, \boldsymbol{H}\,\boldsymbol{f} \right) \|^{2} + \| \left(\boldsymbol{V}_{\boldsymbol{f}}\right)^{-\frac{1}{2}} \boldsymbol{f}\|^{2}\! \right) \!= 0 \\ & \Leftrightarrow - \boldsymbol{H}^{T} \boldsymbol{V}_{\boldsymbol{\epsilon}}^{-1} \left(\boldsymbol{g} - \boldsymbol{H}\,\boldsymbol{f} \right) + \boldsymbol{V}_{\boldsymbol{f}}^{-1} \boldsymbol{f} = 0 \\ & \Leftrightarrow \left[ \boldsymbol{H}^{T} \boldsymbol{V}_{\boldsymbol{\epsilon}}^{-1} \boldsymbol{H} + \boldsymbol{V}_{\boldsymbol{f}}^{-1} \right] \boldsymbol{f} = \boldsymbol{H}^{T} \boldsymbol{V}_{\boldsymbol{\epsilon}}^{-1} \boldsymbol{g} \\ & \Rightarrow {\widehat{\boldsymbol{f}}}_{\text{JMAP}} = \left[ \boldsymbol{H}^{T} \boldsymbol{V}_{\boldsymbol{\epsilon}}^{-1} \boldsymbol{H} + \boldsymbol{V}_{\boldsymbol{f}}^{-1} \right]^{-1} \boldsymbol{H}^{T} \boldsymbol{V}_{\boldsymbol{\epsilon}}^{-1} \boldsymbol{g} \end{aligned}}} $$

∙ With respect to $\phantom {\dot {i}\!}{v}_{{\epsilon }_{i}}$, *i*∈{1,2,…,*N*}:

$$\begin{aligned} \frac{\partial \mathcal{L}\!\left({\widehat{\boldsymbol{f}}}, {\boldsymbol{v}_{\boldsymbol{\epsilon}}}, {\widehat{\boldsymbol{v}_{\boldsymbol{f}}}}\right)}{\partial {v}_{{\epsilon}_{i}}} = 0 & \Leftrightarrow \frac{\partial}{\partial {v}_{{\epsilon}_{i}}} \left(\frac{1}{2} \ln \det \left(\boldsymbol{V}_{\boldsymbol{\epsilon}}\right) + \frac{1}{2} \|\boldsymbol{V}_{\boldsymbol{\epsilon}}^{-\frac{1}{2}} \left(\boldsymbol{g} - \boldsymbol{H}\,\boldsymbol{f}\right)\|^{2} + \left(\alpha_{\epsilon 0} + 1 \right) \ln {v}_{{\epsilon}_{i}} + \beta_{\epsilon 0} {v}_{{\epsilon}_{i}}^{-1} \right) = 0 \\[-2pt] & \Leftrightarrow \frac{\partial}{\partial {v}_{{\epsilon}_{i}}} \left(\left(\alpha_{\epsilon 0} + 1 + \frac{1}{2} \right) \ln {v}_{{\epsilon}_{i}} + \left[\beta_{\epsilon 0} + \frac{1}{2} \left({g}_{i} - \boldsymbol{H}_{i}\, \boldsymbol{f} \right)^{2} \right] {v}_{{\epsilon}_{i}}^{-1} \right) = 0 \\[-2pt] & \Leftrightarrow \left(\alpha_{\epsilon 0} + 1 + \frac{1}{2} \right) {v}_{{\epsilon}_{i}} - \left(\beta_{\epsilon 0} + \frac{1}{2} \left({g}_{i} - \boldsymbol{H}_{i}\, \boldsymbol{f}\right)^{2} \right) = 0 \\[-2pt] & \Rightarrow {\widehat{{v}_{{\epsilon}_{i}}}}_{JMAP} = \frac{\beta_{\epsilon 0} + \frac{1}{2} \left({g}_{i} - \boldsymbol{H}_{i}\, \boldsymbol{f} \right)^{2}}{\alpha_{\epsilon 0} + 1 + \frac{1}{2}} \end{aligned} $$

∙ With respect to *v*_*f*_, *j*∈{1,2,…,*M*}:

$$\begin{aligned} \frac{\partial \mathcal{L}\!\left({\widehat{\boldsymbol{f}}}, {\widehat{\boldsymbol{v}_{\boldsymbol{\epsilon}}}},{\boldsymbol{v}_{\boldsymbol{f}}}\right)}{\partial{v}_{{f}_{j}}} = 0 & \Leftrightarrow \frac{\partial}{\partial {v}_{{f}_{j}}} \left(\frac{1}{2} \ln \det \left(\boldsymbol{V}_{\boldsymbol{f}}\right) + \frac{1}{2} \|\left(\boldsymbol{V}_{\boldsymbol{f}} \right)^{-\frac{1}{2}} \boldsymbol{f} \|^{2} + \left(\alpha_{f 0} + 1 \right) \ln {v}_{{f}_{j}} + \beta_{f 0} {v}_{{f}_{j}}^{-1} \right) = 0 \\[-3pt] & \Leftrightarrow \frac{\partial}{\partial {v}_{{f}_{j}}} \left(\left[ \alpha_{f 0} + 1 + \frac{1}{2}\right] \ln {v}_{{f}_{j}} + \left[ \beta_{f 0} + \frac{{f}_{{j}^{2}}}{2} \right] {v}_{{f}_{j}}^{-1} \right) = 0 \\[-2pt] & \Leftrightarrow \left(\alpha_{f 0} + 1 + \frac{1}{2} \right) {v}_{{f}_{j}} - \left(\beta_{f 0} + \frac{{f}_{{j}^{2}}}{2} \right) = 0 \\[-3pt] & \Rightarrow {\widehat{{v}_{{f}_{j}}}}_{JMAP} = \frac{\beta_{f 0} + \frac{{f}_{{j}^{2}}}{2}}{\alpha_{f 0} + 1 + \frac{1}{2}} \end{aligned} $$

### Appendix 2

#### Computations for PM estimation via VBA, partial separability

This section presents the computation for the PM estimation, via VBA, partial separability (Subsection 4.2). The analytical expression of the logarithm is as follows:

(33) $$ \begin{aligned} \ln p\!\left(\boldsymbol{f}, \boldsymbol{v}_{\boldsymbol{\epsilon}}, \boldsymbol{v}_{\boldsymbol{f}}|\boldsymbol{g}\right) = & -\frac{1}{2} \ln \det \left(\boldsymbol{V}_{\boldsymbol{\epsilon}} \right) -\frac{1}{2}\| \boldsymbol{V}_{\boldsymbol{\epsilon}}^{-\frac{1}{2}} \left(\boldsymbol{g} - \boldsymbol{H}\,\boldsymbol{f} \right) \|^{2} -\frac{1}{2} \ln \det \left(\boldsymbol{V}_{\boldsymbol{f}} \right) -\frac{1}{2} \|\boldsymbol{V}_{\boldsymbol{f}}^{-\frac{1}{2}} \boldsymbol{f}\|^{2} \\ & -\sum\limits_{i=1}^{N} \left(\alpha_{\epsilon 0} + 1 \right) \ln {v}_{{\epsilon}_{i}} -\sum\limits_{i=1}^{N} \beta_{\epsilon 0} {v}_{{\epsilon}_{i}}^{-1} -\sum\limits_{j=1}^{M} \left(\alpha_{f 0} + 1 \right) \ln {v}_{{f}_{j}} -\sum\limits_{j=1}^{M} \beta_{f 0} {{v}_{{f}_{j}}}^{-1} + C \end{aligned}  $$

**∙ Expression of** ***q***_**1**_ **(** ***f*** **):**The proportionality relation concerning *q*_1_(***f***) established in Eq. (22) refers to ***f***, so in the expression of ln*p* (***f***,***v***_***ε***_,***v***_***f***_|***g***), all the terms free of ***f*** can be regarded as constants:

$$\left\langle \ln p\!\left(\boldsymbol{f}, \boldsymbol{v}_{\boldsymbol{\epsilon}}, \boldsymbol{v}_{\boldsymbol{f}}|\boldsymbol{g}\right)\right\rangle_{q_{2} \left(\boldsymbol{v}_{\boldsymbol{\epsilon}}\right) \; q_{3} \left(\boldsymbol{v}_{\boldsymbol{f}}\right)} = \left\langle C -\frac{1}{2} \| \boldsymbol{V}_{\boldsymbol{\epsilon}}^{-\frac{1}{2}} \left(\boldsymbol{g} - \boldsymbol{H}\, \boldsymbol{f} \right) \|^{2} -\frac{1}{2} \| \boldsymbol{V}_{\boldsymbol{f}}^{-\frac{1}{2}} \boldsymbol{f} \|^{2} \right\rangle_{\;q_{2} \left(\boldsymbol{v}_{\boldsymbol{\epsilon}}\right) \; q_{3} \left(\boldsymbol{v}_{\boldsymbol{f}}\right)} $$

leading to:

(34) $$ \begin{aligned} \left\langle \ln p\!\left(\boldsymbol{f},\boldsymbol{v}_{\boldsymbol{\epsilon}},{v}_{{f}}|\boldsymbol{g}\right) \right\rangle_{q_{2}\left(\boldsymbol{v}_{\boldsymbol{\epsilon}}\right) \; q_{3}\left(\boldsymbol{v}_{\boldsymbol{f}}\right)} = C -\frac{1}{2} \left\langle \|\boldsymbol{V}_{\boldsymbol{\epsilon}}^{-\frac{1}{2}} \left(\boldsymbol{g} - \boldsymbol{H}\,\boldsymbol{f} \right)\|^{2} \right\rangle_{q_{2} \left(\boldsymbol{v}_{\boldsymbol{\epsilon}}\right)} -\frac{1}{2} \left\langle \|\boldsymbol{V}_{\boldsymbol{f}}^{-\frac{1}{2}} \boldsymbol{f}\|^{2} \right\rangle_{q_{3}\left(\boldsymbol{v}_{\boldsymbol{f}}\right)} \end{aligned}  $$

Considering the notation introduced in (10) corresponding to ***V***_***ε***_ and denoting the *i*th line of the matrix ***H*** with ***H***_*i*_, *i*∈{1,2,…,*N*}, we write:

(35) $$ \boldsymbol{V}_{\boldsymbol{\epsilon}}^{-\frac{1}{2}} \left(\boldsymbol{g} - \boldsymbol{H}\,\boldsymbol{f}\right) = \left[ {v}_{{\epsilon}_{1}}^{-1/2} \left({g}_{1} - \boldsymbol{H}_{1} \,\boldsymbol{f} \right) \ldots {v}_{{\epsilon}_{i}}^{-1/2} \left({g}_{i} - \boldsymbol{H}_{i} \,\boldsymbol{f} \right) \ldots {v}_{{\epsilon}_{N}}^{-1/2} \left({g}_{N} - \boldsymbol{H}_{N} \,\boldsymbol{f} \right) \right]^{T}  $$

so the norm is written as:

(36) $$ \| \boldsymbol{V}_{\boldsymbol{\epsilon}}^{-\frac{1}{2}} \left(\boldsymbol{g} - \boldsymbol{H} \,\boldsymbol{f} \right)\|^{2} = \sum\limits_{i=1}^{N} {v}_{{\epsilon}_{i}}^{-1} \left({g}_{i} - \boldsymbol{H}_{i} \,\boldsymbol{f} \right)^{2}  $$

Introducing the notations:

(37) $${} \begin{aligned} {\widetilde{{v}_{{\epsilon}_{i}}^{-1}}} &= \!\int\! {v}_{{\epsilon}_{i}}^{-1} q_{2i}\!\left({v}_{{\epsilon}_{i}}\right) \text{d} {v}_{{\epsilon}_{i}} \;\; ; \; {\widetilde{\boldsymbol{v}_{\boldsymbol{\epsilon}}^{-1}}} = \left[ {\widetilde{{v}_{{\epsilon}_{1}}^{-1}}} \ldots {\widetilde{{v}_{{\epsilon}_{i}}^{-1}}} \ldots {\widetilde{{v}_{{\epsilon}_{N}}^{-1}}} \right]^{T} \; ; \\ {\widetilde{\boldsymbol{V}_{\boldsymbol{\epsilon}}^{-1}}} &= \text{diag} \left({\widetilde{\boldsymbol{v}_{\boldsymbol{\epsilon}}^{-1}}} \right) \end{aligned}   $$

we can write:

(38) $$ \begin{aligned} \left\langle \|\boldsymbol{V}_{\boldsymbol{\epsilon}}^{-\frac{1}{2}} \left(\boldsymbol{g} - \boldsymbol{H}\,\boldsymbol{f} \right) \|^{2}\right\rangle_{q_{2}(\boldsymbol{v}_{\boldsymbol{\epsilon}})} &= \sum\limits_{i=1}^{N} {\widetilde{{v}_{{\epsilon}_{i}}^{-1}}} \left({g}_{i} - \boldsymbol{H}_{i} \,\boldsymbol{f} \right)^{2}\\ &= \| \left({\widetilde{\boldsymbol{V}_{\boldsymbol{\epsilon}}^{-1}}}\right)^{1/2} \left(\boldsymbol{g} - \boldsymbol{H}\,\boldsymbol{f} \right)\|^{2} \end{aligned}   $$

Introducing the notation

(39) $${}\begin{aligned} {\widetilde{{v}_{{f}_{j}}^{-1}}} = \!\int\! {v}_{{f}_{j}}^{-1} q_{3j}\!\left({v}_{{f}_{j}}\right) \text{d} {v}_{{f}_{j}} \;\; ; \;\; {\widetilde{\boldsymbol{v}_{\boldsymbol{f}}^{-1}}} &= \left[ {\widetilde{{v}_{{f}_{1}}^{-1}}} \ldots {\widetilde{{v}_{{f}_{j}}^{-1}}} \ldots {\widetilde{v_{{f}_{M}}^{-1}}} \right]^{T} \; ; \\ {\widetilde{\boldsymbol{V}_{\boldsymbol{f}}^{-1}}} &= \text{diag} \left({\widetilde{{v}_{{f}_{j}}^{-1}}} \right) \end{aligned}   $$

we can write:

(40) $$ \left\langle \|\boldsymbol{V}_{\boldsymbol{f}}^{-\frac{1}{2}} \boldsymbol{f}\|^{2} \right\rangle_{q_{3}(\boldsymbol{v}_{\boldsymbol{f}})} = \| \left({\widetilde{\boldsymbol{V}_{\boldsymbol{\epsilon}}^{-1}}}\right)^{\frac{1}{2}} \boldsymbol{f} \|^{2}   $$

Finally from (34), (38), and (40), for the expression of $\left \langle \ln p\!\left (\boldsymbol {f},\boldsymbol {v}_{\boldsymbol {\epsilon }},v_{f}|\boldsymbol {g}\right) \right \rangle _{q_{2}(\boldsymbol {v}_{\boldsymbol {\epsilon }}) \; q_{3}\left (\boldsymbol {v}_{\boldsymbol {f}}\right)}$, we have:

(41) $${} {\fontsize{9.2pt}{9.6pt}{\begin{aligned} \left\langle \ln p\!\left(\boldsymbol{f},\boldsymbol{v}_{\boldsymbol{\epsilon}},\boldsymbol{v}_{\boldsymbol{f}}|\boldsymbol{g}\right) \right\rangle_{q_{2}(\boldsymbol{v}_{\boldsymbol{\epsilon}}) \; q_{3}\left(\boldsymbol{v}_{\boldsymbol{f}}\right)} = C& -\frac{1}{2} \| \left({\widetilde{\boldsymbol{V}_{\boldsymbol{\epsilon}}^{-1}}} \right)^{1/2} \left(\boldsymbol{g} - \boldsymbol{H}\,\boldsymbol{f} \right) \|^{2} \\&-\frac{1}{2} \| \left({\widetilde{\boldsymbol{V}_{\boldsymbol{f}}^{-1}}} \right)^{\frac{1}{2}} \boldsymbol{f} \|^{2} \end{aligned}}}   $$

and via the first proportionality from (22) and the notation:

(42) $$ J(\boldsymbol{f}) = \| \left({\widetilde{\boldsymbol{V}_{\boldsymbol{\epsilon}}^{-1}}}\right)^{1/2} \left(\boldsymbol{g} - \boldsymbol{H}\,\boldsymbol{f} \right) \|^{2} + \| \left({\widetilde{\boldsymbol{V}_{\boldsymbol{f}}^{-1}}} \right)^{\frac{1}{2}} \boldsymbol{f} \|^{2}   $$

the probability *q*_1_(***f***) can be expressed by the following proportionality:

(43) $$ q_{1}(\boldsymbol{f}) \propto \left\{ -\frac{1}{2} J(\boldsymbol{f}) \right\}   $$

The criterion *J*(***f***) introduced in Eq. (42) is quadratic in ***f***. Equation 43 establishes a proportionality relation between *q*_1_(***f***) and an exponential function having as argument a quadratic criterion. This leads to the following:

##### Intermediate conclusion 1.

The probability distribution function *q*_1_(***f***) is a multivariate normal distribution.

Of course, the mean is given by the solution that minimizes the criterion *J*(***f***), i.e., the solution of the equation $\frac {\partial J(\boldsymbol {f})}{\partial \boldsymbol {f}}=0$ (and in particular, this is the same criterion that arrived in the MAP estimation technique for ***f***, with some formal differences):

(44) $$ \begin{aligned} \frac{\partial J(\boldsymbol{f})}{\partial \boldsymbol{f}} = 0 & \Rightarrow {\widehat{\boldsymbol{f}}}_{\text{PM}} = \left(\boldsymbol{H}^{T} {\widetilde{\boldsymbol{V}_{\boldsymbol{\epsilon}}^{-1}}} \boldsymbol{H} + {\widetilde{\boldsymbol{V}_{\boldsymbol{f}}^{-1}}} \right)^{-1} \boldsymbol{H}^{T} {\widetilde{\boldsymbol{V}_{\boldsymbol{\epsilon}}^{-1}}} \boldsymbol{g} \end{aligned}  $$

The corresponding covariance matrix is computed by identification. On the one hand, we have the following relation:

(45) $${} {\fontsize{8.4pt}{9.6pt}{\begin{aligned} \mathcal{N}\left(\boldsymbol{f}|\,{\widehat{\boldsymbol{f}}}_{\text{PM}}, {\widehat{\boldsymbol{\Sigma}}}\right) \propto \left(\det ({\widehat{\boldsymbol{\Sigma}}}) \right)^{\frac{1}{2}} \!\exp\! \left\{ -\frac{1}{2} \left(\boldsymbol{f}-{\widehat{\boldsymbol{f}}}_{\text{PM}}\right)^{T} {\widehat{\boldsymbol{\Sigma}}}^{-1} \left(\boldsymbol{f}-{\widehat{\boldsymbol{f}}}_{\text{PM}}\right) \right\} \end{aligned}}}   $$

One the other hand, we have the following proportionality, given by Eq. (43):

(46) $$ \mathcal{N}\left(\boldsymbol{f} | \,{\widehat{\boldsymbol{f}}}_{\text{PM}}, {\widehat{\boldsymbol{\Sigma}}} \right) \propto q_{1}(\boldsymbol{f}) \propto \exp \left\{ -\frac{1}{2} J(\boldsymbol{f}) \right\}   $$

So, the covariance matrix ${\widehat {\boldsymbol {\Sigma }}}$ must respect the following relation:

(47) $$ \left(\boldsymbol{f} - {\widehat{\boldsymbol{f}}}_{\text{PM}}\right)^{T} {\widehat{\boldsymbol{\Sigma}}}^{-1} \left(\boldsymbol{f} - {\widehat{\boldsymbol{f}}}_{\text{PM}}\right) \equiv J(\boldsymbol{f}),   $$

where the sign ≡ represents an equality between the two terms until a free ***f*** term. If we consider the covariance matrix

(48) $$ {\widehat{\boldsymbol{\Sigma}}} = \left(\boldsymbol{H}^{T} {\widetilde{\boldsymbol{V}_{\boldsymbol{\epsilon}}^{-1}}} \boldsymbol{H} + {\widetilde{\boldsymbol{V}_{\boldsymbol{f}}^{-1}}} \right)^{-1}   $$

we have the following equalities:

(49) $${} {\fontsize{8.8pt}{9.6pt}{\begin{aligned} \left(\boldsymbol{f}-{\widehat{\boldsymbol{f}}}_{\text{PM}}\right)^{T} {\widehat{\boldsymbol{\Sigma}}}^{-1} \left(\boldsymbol{f} - {\widehat{\boldsymbol{f}}}_{\text{PM}}\right) & = \left(\boldsymbol{f} - {\widehat{\boldsymbol{\Sigma}}}\boldsymbol{H}^{T} {\widetilde{\boldsymbol{V}_{\boldsymbol{\epsilon}}^{-1}}} \boldsymbol{g}\right)^{T} {\widehat{\boldsymbol{\Sigma}}}^{-1}\\&\quad\, \left(\boldsymbol{f} - {\widehat{\boldsymbol{\Sigma}}}\boldsymbol{H}^{T} {\widetilde{\boldsymbol{V}_{\boldsymbol{\epsilon}}^{-1}}} \boldsymbol{g}\right) \\ & = \left(\boldsymbol{f}^{T} - \boldsymbol{g}^{T} {\widetilde{\boldsymbol{V}_{\boldsymbol{\epsilon}}^{-1}}} \boldsymbol{H} {\widehat{\boldsymbol{\Sigma}}}\right)\\&\quad\, \left({\widehat{\boldsymbol{\Sigma}}}^{-1} \boldsymbol{f} - \boldsymbol{H}^{T} {\widetilde{\boldsymbol{V}_{\boldsymbol{\epsilon}}^{-1}}} \boldsymbol{g}\right) \\ & = \boldsymbol{f}^{T} \left(\boldsymbol{H}^{T} {\widetilde{\boldsymbol{V}_{\boldsymbol{\epsilon}}^{-1}}} \boldsymbol{H} + {\widetilde{\boldsymbol{V}_{\boldsymbol{f}}^{-1}}} \right)\,\\&\quad\; \boldsymbol{f} - 2 \, \boldsymbol{f}^{T}\boldsymbol{H}^{T} {\widetilde{\boldsymbol{V}_{\boldsymbol{\epsilon}}^{-1}}}\boldsymbol{g} + C, \end{aligned}}}  $$

where we have used the equality $\boldsymbol {f}^{T} \boldsymbol {H}^{T} {\widetilde {\boldsymbol {V}_{\boldsymbol {\epsilon }}^{-1}}} \boldsymbol {g} = \boldsymbol {g}^{T} {\widetilde {\boldsymbol {V}_{\boldsymbol {\epsilon }}^{-1}}} \boldsymbol {H}\, \boldsymbol {f}$, as a consequence of the fact that one term is the transpose of the other and the term is a scalar. We also used the fact that ${\widehat {\boldsymbol {\Sigma }}} = {\widehat {\boldsymbol {\Sigma }}}^{T}$ and $\boldsymbol {g}^{T} {\widetilde {\boldsymbol {V}_{\boldsymbol {\epsilon }}^{-1}}} \boldsymbol {H}\left (\boldsymbol {H}^{T} {\widetilde {\boldsymbol {V}_{\boldsymbol {\epsilon }}^{-1}}} \boldsymbol {H} + {\widetilde {\boldsymbol {V}_{\boldsymbol {f}}^{-1}}} \right)^{-1} \boldsymbol {H}^{T}{\widetilde {\boldsymbol {V}_{\boldsymbol {\epsilon }}^{-1}}} \boldsymbol {g}$ was viewed as a constant *C*. We also have the following equalities:

(50) $${} \begin{aligned} J\left(\,\boldsymbol{f}\right) &= \| \left({\widetilde{\boldsymbol{V}_{\boldsymbol{\epsilon}}^{-1}}}\right)^{1/2} \left(\boldsymbol{g} - \boldsymbol{H}\,\boldsymbol{f}\right) \|^{2} + \| \left({\widetilde{\boldsymbol{V}_{\boldsymbol{f}}^{-1}}}\right)^{\frac{1}{2}} \boldsymbol{f} \|^{2}\\ &= \left(\boldsymbol{g}^{T} - \boldsymbol{f}^{T}\boldsymbol{H}^{T}\right) {\widetilde{\boldsymbol{V}_{\boldsymbol{\epsilon}}^{-1}}} \left(\boldsymbol{g} - \boldsymbol{H}\, \boldsymbol{f}\right) + \boldsymbol{f}^{T} {\widetilde{\boldsymbol{V}_{\boldsymbol{f}}^{-1}}}\, \boldsymbol{f} \\ &= \boldsymbol{f}^{T} \left(\boldsymbol{H}^{T} {\widetilde{\boldsymbol{V}_{\boldsymbol{\epsilon}}^{-1}}} \boldsymbol{H} + {\widetilde{\boldsymbol{V}_{\boldsymbol{f}}^{-1}}}\right)\, \boldsymbol{f} - 2 \, \boldsymbol{f}^{T} \boldsymbol{H}^{T} {\widetilde{\boldsymbol{V}_{\boldsymbol{\epsilon}}^{-1}}} \boldsymbol{g} + C. \end{aligned}  $$

Equations 49 and (50) show that equality imposed in (47) is verified with the covariance matrix defined as in (48). So, for the normal distribution $\mathcal {N}\left (\boldsymbol {f} | \,{\widehat {\boldsymbol {f}}}, {\widehat {\boldsymbol {\Sigma }}} \right)$ proportional to *q*_1_(***f***), we have the following parameters:

(51) $${} {\fontsize{8.4pt}{9.6pt}{\begin{aligned} q_{1}(\boldsymbol{f}) = \mathcal{N}\left(\boldsymbol{f} | \,{\widehat{\boldsymbol{f}}}_{\text{PM}}, {\widehat{\boldsymbol{\Sigma}}}\right), \left\{ \begin{array}{l} {\widehat{\boldsymbol{f}}}_{\text{PM}} = \left(\boldsymbol{H}^{T} {\widetilde{\boldsymbol{V}_{\boldsymbol{\epsilon}}^{-1}}} \boldsymbol{H} + {\widetilde{\boldsymbol{V}_{\boldsymbol{f}}^{-1}}}\right)^{-1} \boldsymbol{H}^{T} {\widetilde{\boldsymbol{V}_{\boldsymbol{\epsilon}}^{-1}}} \boldsymbol{g} \\{\widehat{\boldsymbol{\Sigma}}} = \left(\boldsymbol{H}^{T} {\widetilde{\boldsymbol{V}_{\boldsymbol{\epsilon}}^{-1}}} \boldsymbol{H} + {\widetilde{\boldsymbol{V}_{\boldsymbol{f}}^{-1}}} \right)^{-1} \end{array}\right. \end{aligned}}}   $$

**∙ Expression of** $\boldsymbol {q_{2i}\!\left ({v}_{{\epsilon }_{i}}\right):}$

The proportionality relation concerning $\phantom {\dot {i}\!}q_{2i}\!\left ({v}_{{\epsilon }_{i}}\right)$ established in Eq. (22) refers to $\phantom {\dot {i}\!}v_{{\epsilon }_{i}}$, so in the expression of ln*p* (***f***,***v***_***ε***_,***v***_***f***_|***g***), all the terms free of $\phantom {\dot {i}\!}v_{{\epsilon }_{i}}$ can be regarded as constants:

(52) $${} \begin{aligned} &\left\langle \ln p\!\left(\boldsymbol{f},\boldsymbol{v}_{\boldsymbol{\epsilon}},\boldsymbol{v}_{\boldsymbol{f}}|\boldsymbol{g}\right) \right\rangle_{q_{1}(\boldsymbol{f}) \; q_{2-i}\left(v_{{\epsilon}_{i}}\right) \; q_{3}\left(\boldsymbol{v}_{\boldsymbol{f}}\right)}\\ & = C -\frac{1}{2} \left\langle \ln \det \left(\boldsymbol{V}_{\boldsymbol{\epsilon}}\right) \right\rangle_{q_{2-i}\left({v}_{{\epsilon}_{i}}\right)} \\&\qquad\;\! - \left(\alpha_{\epsilon 0} + 1\right) \ln {v}_{{\epsilon}_{i}} \\ & \qquad -\frac{1}{2} \left\langle \| \boldsymbol{V}_{\boldsymbol{\epsilon}}^{-\frac{1}{2}} \left(\boldsymbol{g} - \boldsymbol{H}\,\boldsymbol{f} \right)\|^{2} \right\rangle_{q_{1}(\boldsymbol{f}) \; q_{2-i}\left({v}_{{\epsilon}_{i}}\right)} - \beta_{\epsilon 0} {v}_{{\epsilon}_{i}}^{-1} \end{aligned}  $$

For the first integral, it is trivial to verify:

(53) $$ \left\langle \ln \det \left(\boldsymbol{V}_{\boldsymbol{\epsilon}} \right)\right\rangle_{q_{2-i}\left({v}_{{\epsilon}_{i}}\right)} = C + \ln {v}_{{\epsilon}_{i}}   $$

For the second integral, we have the following development:

(54) $${} {\fontsize{8.8pt}{9.6pt}{\begin{aligned} \left\langle \|\boldsymbol{V}_{\boldsymbol{\epsilon}}^{-\frac{1}{2}} \left(\boldsymbol{g} - \boldsymbol{H}\,\boldsymbol{f} \right)\|^{2}\right\rangle_{q_{1}(\boldsymbol{f}) \; q_{2-i}\left({v}_{{\epsilon}_{i}}\right)} = \left\langle \|{\widetilde{\boldsymbol{V}_{\boldsymbol{\epsilon} -{i}}^{-1}}}^{\frac{1}{2}} \left(\boldsymbol{g} - \boldsymbol{H}\,\boldsymbol{f} \right)\|^{2} \right\rangle_{q_{1}(\boldsymbol{f})} \end{aligned}}}  $$

where we have introduced the following notations:

(55) $$ \begin{aligned} {\widetilde{\boldsymbol{v}_{\boldsymbol{\epsilon} -{i}}^{-1}}} &= \left[ {\widetilde{{v}_{{\epsilon}_{1}}^{-1}}} \; \ldots \; {\widetilde{{v}_{{\epsilon}_{i-1}}^{-1}}} \; {{v}_{{\epsilon}_{i}}^{-1}} \; {\widetilde{{v}_{{\epsilon}_{i+1}}^{-1}}} \; \ldots \; {\widetilde{{v}_{{\epsilon}_{N}}^{-1}}} \right]^{T} \;\; ; \\ {\widetilde{\boldsymbol{V}_{\boldsymbol{\epsilon} -{i}}^{-1}}} &= \text{diag} \left({\widetilde{\boldsymbol{v}_{\boldsymbol{\epsilon}-{i}}^{-1}}}\right) \end{aligned}   $$

Again, using the fact that *q*_1_(***f***) is a multivariate normal distribution, we have:

(56) $${} \begin{aligned} \left\langle \|{\widetilde{\boldsymbol{V}_{\boldsymbol{\epsilon}-{i}}^{-1}}}^{\frac{1}{2}} \left(\boldsymbol{g} - \boldsymbol{H}\,\boldsymbol{f} \right)\|^{2} \right\rangle_{q_{1}(\boldsymbol{f})} &= \| {\widetilde{\boldsymbol{V}_{\boldsymbol{\epsilon}-{i}}^{-1}}}^{\frac{1}{2}} \left(\boldsymbol{g} - \boldsymbol{H}\,{\widehat{\boldsymbol{f}}}_{\text{PM}}\right) \|^{2} \\&\quad + \text{Tr} \left(\boldsymbol{H}^{T} {\widetilde{\boldsymbol{V}_{\boldsymbol{\epsilon}-{i}}^{-1}}} \boldsymbol{H} {\widehat{\boldsymbol{\Sigma}}}\right) \end{aligned}  $$

and considering as constants all terms free of $\phantom {\dot {i}\!}{v}_{{\epsilon }_{{i}}}$, we have:

(57) $$\begin{array}{@{}rcl@{}} \| {\widetilde{\boldsymbol{V}_{\boldsymbol{\epsilon}-{i}}^{-1}}}^{\frac{1}{2}} \left(\boldsymbol{g} - \boldsymbol{H} \,{\widehat{\boldsymbol{f}}}_{\text{PM}} \right) \|^{2} &=& C + {v}_{{\epsilon}_{i}}^{-1} \left({g}_{i} - \boldsymbol{H}_{i} \,{\widehat{\boldsymbol{f}}}_{\text{PM}} \right)^{2} \;\; ;\notag\\ \;\; \text{Tr} \left(\boldsymbol{H}^{T} {\widetilde{\boldsymbol{V}_{\boldsymbol{\epsilon}-{i}}^{-1}}} \boldsymbol{H} {\widehat{\boldsymbol{\Sigma}}} \right) &=& C + {v}_{{\epsilon}_{i}}^{-1}\boldsymbol{H}_{i} {\widehat{\boldsymbol{\Sigma}}} \boldsymbol{H}_{i}^{T} \end{array} $$

where ***H***_*i*_ is the line i of the matrix ***H***, so we can conclude:

(58) $$ \begin{aligned} &\left\langle \| \boldsymbol{V}_{\boldsymbol{\epsilon}}^{-\frac{1}{2}} \left(\boldsymbol{g} - \boldsymbol{H}\,\boldsymbol{f}\right) \|^{2} \right\rangle_{q_{1}(\boldsymbol{f}) \; q_{2-i}\left({v}_{{\epsilon}_{i}}\right)} \\&= C + \left[ \boldsymbol{H}_{i} {\widehat{\boldsymbol{\Sigma}}} \boldsymbol{H}_{i}^{T} + \left({g}_{i} - \boldsymbol{H}_{i} \,{\widehat{\boldsymbol{f}}}_{\text{PM}} \right)^{2} \right] {v}_{{\epsilon}_{i}}^{-1} \end{aligned}   $$

From (52) via (53) and (58), we get:

$$\begin{aligned} &\left\langle \ln p\!\left(\boldsymbol{f},\boldsymbol{z}, \boldsymbol{v}_{\boldsymbol{\epsilon}}, \boldsymbol{v}_{\boldsymbol{f}}|\boldsymbol{g}\right) \right\rangle_{q_{1}(\boldsymbol{f}) \; q_{2-i}\left(v_{{\epsilon}_{i}}\right) \; q_{3}\left(\boldsymbol{v}_{\boldsymbol{f}}\right)} \\& = C - \left(\alpha_{\epsilon 0} + 1 + \frac{1}{2}\right) \ln {v}_{{\epsilon}_{i}} \\ & \quad \left(\beta_{\epsilon 0} + \frac{1}{2} \left[\boldsymbol{H}_{i} {\widehat{\boldsymbol{\Sigma}}} \boldsymbol{H}_{i}^{T} + \left({g}_{i} - \boldsymbol{H}_{i} \,{\widehat{\boldsymbol{f}}}_{\text{PM}} \right)^{2} \right] \right) {v}_{{\epsilon}_{i}}^{-1}\end{aligned} $$

from which we can establish the proportionality corresponding to $\phantom {\dot {i}\!}q_{2i}({v}_{{\epsilon }_{{i}}})$:

(59) $${} \begin{aligned} q_{2i}\left({v}_{{\epsilon}_{i}}\right) \propto {v}_{{\epsilon}_{i}}^{-\left(\alpha_{\epsilon 0} + 1 + \frac{1}{2}\right)}& \exp \left\{-\left(\beta_{\epsilon 0} + \frac{1}{2} \left[\boldsymbol{H}_{i} {\widehat{\boldsymbol{\Sigma}}} \boldsymbol{H}_{i}^{T} \right.\right.\right.\\ &\qquad +\left.\left.\left. \left({g}_{i} - \boldsymbol{H}_{i} \,{\widehat{\boldsymbol{f}}}_{\text{PM}} \right)^{2} \right] \right) {v}_{{\epsilon}_{i}}^{-1} \right\} \end{aligned}   $$

Equation (59) leads to the following.

##### Intermediate conclusion 2.

The probability distribution function $q_{3i}\left ({v}_{{\epsilon }_{i}}\right)$ is an inverse gamma distribution, with the parameters $\alpha _{\epsilon _{i}}$ and $\beta _{\epsilon _{i}}$:

We can write:

(60) $${} {\fontsize{7.6pt}{9.6pt}{\begin{aligned} q_{2i}\left(v_{{\epsilon}_{i}}\right) = \mathcal{I}\mathcal{G} \left(v_{{\epsilon}_{i}}|\alpha_{\epsilon_{i}},\beta_{\epsilon_{i}}\right), \left\{ \begin{array}{l} \!\!\alpha_{\epsilon_{i}} = \alpha_{\epsilon 0} + \frac{1}{2} \\ \!\!\beta_{\epsilon_{i}} = \beta_{\epsilon 0} +\frac{1}{2} \left[\boldsymbol{H}_{i} {\widehat{\boldsymbol{\Sigma}}} \boldsymbol{H}_{i}^{T} + \left({g}_{i} - \boldsymbol{H}_{i} \,{\widehat{\boldsymbol{f}}}_{\text{PM}} \right)^{2} \right] \end{array}\right. \end{aligned}}}   $$

***∙*** **Expression of** $\phantom {\dot {i}\!}\boldsymbol {q_{3j}({v}_{{f}_{j}}):}$

The proportionality relation concerning $\phantom {\dot {i}\!}q_{3j}\left (v_{f_{j}}\right)$ established in Eq. (22) refers to $\phantom {\dot {i}\!}v_{{f}_{j}}$, so in the expression of ln*p* (***f***,***z***,***v***_***ε***_,***v***_***f***_|***g***), all the terms free of $v_{f_{j}}$ can be regarded as constants:

(61) $$ \begin{aligned} &\left\langle \ln p\!\left(\boldsymbol{f},\boldsymbol{v}_{\boldsymbol{\epsilon}},\boldsymbol{v}_{\boldsymbol{f}}|\boldsymbol{g}\right) \right\rangle_{q_{1}(\boldsymbol{f}) \; q_{2}(\boldsymbol{v}_{\boldsymbol{\epsilon}}) \; q_{3-j}\left(v_{f_{j}}\right)}\\ &= -\frac{1}{2} \left\langle \ln \det \left(\boldsymbol{V}_{\boldsymbol{f}}\right) \right\rangle_{q_{3-j}\left({v}_{{f}_{j}}\right)} -\left(\alpha_{f 0} + 1 \right) \ln {v}_{{f}_{j}} \\ & \quad-\frac{1}{2} \left\langle \| \left(\boldsymbol{V}_{\boldsymbol{f}}\right)^{-\frac{1}{2}} \boldsymbol{f} \|^{2} \right\rangle_{q_{1}(\boldsymbol{f}) \; q_{3-j}(v_{f_{j}})} -\beta_{f 0} {v}_{{f}_{j}}^{-1} \end{aligned}  $$

Considering all $\phantom {\dot {i}\!}{v}_{{f}_{j}}$ free terms as constants, it is easy to verify:

(62) $$ \left\langle \ln \det \left(\boldsymbol{V}_{\boldsymbol{f}}\right) \right\rangle_{q_{3-j}\left({v}_{{f}_{j}}\right)} = C + \ln {v}_{{f}_{j}}   $$

For the second integral:

(63) $$ \left\langle \| \left(\boldsymbol{V}_{\boldsymbol{f}}\right)^{-\frac{1}{2}} \boldsymbol{f} \|^{2} \right\rangle_{q_{1}(\boldsymbol{f}) \; q_{3-j}\left(v_{f_{j}}\right)} = \left\langle \| \left({\widetilde{\boldsymbol{V}_{\boldsymbol{f}-{i}}^{-1}}}\right)^{\frac{1}{2}} \boldsymbol{f} \|^{2} \right\rangle_{q_{1}(\boldsymbol{f})}  $$

where we have introduced the notations:

(64) $$ \begin{aligned} {\widetilde{\boldsymbol{v}_{\boldsymbol{f}-{i}}^{-1}}} &= \left[ {\widetilde{{v}_{{f}_{1}}^{-1}}} \; \ldots \; {\widetilde{{v}_{{f}_{i-1}}^{-1}}} \; {{v}_{{f}_{i}}^{-1}} \; {\widetilde{{v}_{{f}_{i+1}}^{-1}}} \; \ldots \; {\widetilde{{v}_{{f}_{N}}^{-1}}} \right]^{T} \;\; ;\\ {\widetilde{\boldsymbol{V}_{\boldsymbol{f}-{i}}^{-1}}} &= \text{diag} \left({\widetilde{\boldsymbol{v}_{\boldsymbol{f}-{i}}^{-1}}}\right) \end{aligned}   $$

Considering the fact that *q*_1_(***f***) was established as a multivariate normal distribution, we have:

(65) $${} {\fontsize{9.6pt}{9.6pt}{\begin{aligned} \left\langle \| \left({\widetilde{\boldsymbol{V}_{\boldsymbol{f}-{i}}^{-1}}}\right)^{\frac{1}{2}} \boldsymbol{f} \|^{2} \right\rangle_{q_{1}(\boldsymbol{f})} &= \| \left({\widetilde{\boldsymbol{V}_{\boldsymbol{f}-{i}}^{-1}}}\right)^{\frac{1}{2}} {\widehat{\boldsymbol{f}}}_{\text{PM}} \|^{2} + \text{Tr} \left({\widetilde{\boldsymbol{V}_{\boldsymbol{f}-i}^{-1}}} {\widehat{\boldsymbol{\Sigma}}}\right)\\ &= C + {{v}_{{f}_{i}}^{-1}} \left({\widehat{{f}_{j}}}_{\text{PM}}^{2} + {\widehat{\boldsymbol{\Sigma}}}_{jj}\right) \end{aligned}}}   $$

From (61) via (62) and (65), we get:

(66) $${} {\fontsize{8.4pt}{9.6pt}{\begin{aligned} \left\langle \ln p\!\left(\boldsymbol{f},\boldsymbol{v}_{\boldsymbol{\epsilon}},\boldsymbol{v}_{\boldsymbol{f}}|\boldsymbol{g}\right) \right\rangle_{q_{1}(\boldsymbol{f}) \; q_{2}(\boldsymbol{v}_{\boldsymbol{\epsilon}}) \; q_{3-j}\left(v_{f_{j}}\right)} = &-\left(\alpha_{f 0} + \frac{1}{2} + 1 \right) \ln {v}_{f}\\ &- \left(\!\beta_{f 0} + \frac{1}{2} \left({\widehat{{f}_{j}}}_{\text{PM}}^{2} + {\widehat{\boldsymbol{\Sigma}}}_{jj} \right)\!\! \right)\! {v}_{f}^{-1} \end{aligned}}}  $$

from which we can establish the proportionality corresponding to $q_{4}\left ({v}_{{f}_{j}}\right)$:

(67) $${} {\fontsize{8.8pt}{9.6pt}{\begin{aligned} q_{3j}\left({v}_{{f}_{j}}\right) \propto {v}_{{f}_{j}}^{-\left(\alpha_{f 0} + \frac{1}{2} + 1 \right)} \exp \left\{-\left[\beta_{f 0} + \frac{1}{2} \left({\widehat{{f}_{j}}}_{\text{PM}}^{2} + {\widehat{\boldsymbol{\Sigma}}}_{jj} \right) \right] {v}_{f}^{-1} \right\} \end{aligned}}}   $$

Equation (67) leads to the following.

##### Intermediate conclusion 3.

The probability distribution function *q*_4_(*v*_*f*_) is an inverse gamma distribution, with the parameters $\alpha _{f_{j}}$ and $\beta _{f_{j}}$:

(68) $${} q_{3j}\left({v}_{{f}_{j}}\right) = \mathcal{I}\mathcal{G} \left({v}_{{f}_{j}}|\alpha_{f_{j}},\beta_{f_{j}}\right), \left\{ \begin{array}{l} \alpha_{f_{j}} = \alpha_{f 0} + \frac{1}{2} \\ \beta_{f_{j}} = \beta_{f 0} + \frac{1}{2} \left({\widehat{{f}_{j}}}_{\text{PM}}^{2} + {\widehat{\boldsymbol{\Sigma}}}_{jj} \right) \end{array}\right.   $$

Expressions (51), (60), and (68) resume the distributions families and the corresponding parameters for *q*_1_(***f***), $q_{2i}\left (v_{{\epsilon }_{i}}\right)$, *i*∈{1,2,…,*N*} and $q_{3j}\left (v_{f_{j}}\right)$, *j*∈{1,2,…,*M*}. However, the parameters corresponding to the multivariate normal distribution are expressed via ${\widetilde {\boldsymbol {V}_{\boldsymbol {\epsilon }}^{-1}}}$ and ${\widetilde {\boldsymbol {V}_{\boldsymbol {f}}^{-1}}}$ (and by extension, all elements forming the three matrices ${\widetilde {v_{{\epsilon }_{i}}^{-1}}}$, *i*∈{1,2,…,*N*} and ${\widetilde {v_{f_{j}}^{-1}}}$, *j*∈{1,2,…,*M*}).

**∙ Computation of** ${\widetilde {\boldsymbol {V}_{\boldsymbol {\epsilon }}^{-1}}}$ **,** ${\widetilde {\boldsymbol {V}_{\boldsymbol {f}}^{-1}}}$ **:**For an inverse gamma distribution with parameters *α* and *β*, $\mathcal {I}\mathcal {G}\left (x|\alpha, \beta \right)$, the following relation holds:

$$\left\langle x^{-1} \right\rangle_{\mathcal{I}\mathcal{G}(x|\alpha,\beta)} = \frac{\alpha}{\beta} $$

The prove of the above relation is done by direct computation, using the analytical expression of the inverse gamma distribution:

$${} \begin{aligned} \left\langle x^{-1} \right\rangle_{\mathcal{I}\mathcal{G}(x|\alpha,\beta)} & = \int x^{-1} \frac{{\beta}^{\alpha}}{\Gamma(\alpha)} x^{-\alpha-1} \exp \left\{-\frac{\beta}{x}\right\} \text{d} x\\ &= \frac{{\beta}^{\alpha}}{\Gamma(\alpha)} \frac{\Gamma(\alpha + 1)}{{\beta}^{\alpha+1}} \int \frac{{\beta}^{\alpha+1}}{\Gamma(\alpha + 1)} x^{-(\alpha + 1)-1} \\&\quad \exp \left\{-\frac{\beta}{x}\right\} \text{d} x = \\ & = \frac{\alpha}{\beta} \underbrace{\int \mathcal{I}\mathcal{G}(x|\alpha + 1,\beta)}_{1} \text{d} x = \frac{\alpha}{\beta} \end{aligned} $$

Since $q_{2i}\left ({v}_{{\epsilon }_{i}}\right)$, *i*∈{1,2,…,*N*} and $q_{3j}\left (v_{f_{j}}\right)$, *j*∈{1,2,…,*M*} are inverse gamma distributions, with parameters $\alpha _{\epsilon _{i}}$ and $\beta _{\epsilon _{i}}$, *i*∈{1,2,…,*N*}, respectively, $\alpha _{f_{j}}$ and $\beta _{f_{j}}$, *j*∈{1,2,…,*M*}, we can express the expectancies ${\widetilde {v_{{\epsilon }_{i}}^{-1}}}$ and ${\widetilde {v_{f_{j}}^{-1}}}$ via the parameters of the two inverse gamma distributions using the result above:

(69) $$ {\widetilde{{v}_{{\epsilon}_{i}}^{-1}}} = \frac{\alpha_{\epsilon_{i}}}{\beta_{\epsilon_{i}}} \;\;\; ; \;\;\; {\widetilde{{v}_{f}^{-1}}} = \frac{\alpha_{f}}{\beta_{f}}   $$

Using the notation introduced in (37) and (39), we obtain:

(70) $$ \begin{aligned} {\widetilde{\boldsymbol{V}_{\boldsymbol{\epsilon}}^{-1}}} &= \left[ \begin{array}{ccccc} \frac{\alpha_{\epsilon_{1}}}{\beta_{\epsilon_{1}}} & \ldots & 0 & \ldots & 0 \\ \vdots & \ddots & \vdots & \ddots & \vdots \\ 0 & \ldots & \frac{\alpha_{\epsilon_{i}}}{\beta_{\epsilon_{i}}} & \ldots & 0 \\ \vdots & \ddots & \vdots & \ddots & \vdots \\ 0 & \ldots & 0 & \ldots & \frac{\alpha_{\epsilon_{N}}}{\beta_{\epsilon_{N}}} \\ \end{array}\right] = {\widehat{\boldsymbol{V}_{\boldsymbol{\epsilon}}^{-1}}} \;\; ;\\ \;\; {\widetilde{\boldsymbol{V}_{\boldsymbol{f}}^{-1}}} &= \left[ \begin{array}{ccccc} \frac{\alpha_{f_{1}}}{\beta_{f_{1}}} & \ldots & 0 & \ldots & 0 \\ \vdots & \ddots & \vdots & \ddots & \vdots \\ 0 & \ldots & \frac{\alpha_{f_{j}}}{\beta_{f_{j}}} & \ldots & 0 \\ \vdots & \ddots & \vdots & \ddots & \vdots \\ 0 & \ldots & 0 & \ldots & \frac{\alpha_{f_{M}}}{\beta_{f_{M}}} \\ \end{array}\right] = {\widehat{\boldsymbol{V}_{\boldsymbol{f}}^{-1}}} \end{aligned}   $$

##### Remark.

In Eq. (70), we have introduced other notations for ${\widetilde {\boldsymbol {V}_{\boldsymbol {f}}^{-1}}}$ and ${\widetilde {\boldsymbol {V}_{\boldsymbol {\epsilon }}^{-1}}}$. All three values were expressed during the model via unknown expectancies, but at this point, we arrive at expressions that do not contain any more integrals to be computed. Therefore, the new notations represent the final expressions for the density functions *q* that depend only on numerical hyperparameters, set in the prior modeling.

### Appendix 3

#### Computations for PM estimation via VBA, full separability

This section presents the computation for the PM estimation, via VBA, full separability (Subsection 4.3). The expression of the logarithm ln*p*(***f***,***v***_***ε***_,***v***_***f***_|***g***) was established in the preview section (Eq. (33)).

**∙ Expression of** ***q***_***1j***_ ***(f***_***j***_ ***)*** **:**Using Eq. (41):

(71) $$ \begin{aligned} &\left\langle \ln p\!\left(\boldsymbol{f},\boldsymbol{v}_{\boldsymbol{\epsilon}},\boldsymbol{v}_{\boldsymbol{f}}|\boldsymbol{g}\right) \right\rangle_{q_{1-j}({f}_{j}) \; q_{2}(\boldsymbol{v}_{\boldsymbol{\epsilon}}) \; q_{3}\left(\boldsymbol{v}_{\boldsymbol{f}}\right)}\\ &= C -\frac{1}{2} \left\langle \| \left({\widetilde{\boldsymbol{V}_{\boldsymbol{\epsilon}}^{-1}}}\right)^{1/2} \left(\boldsymbol{g} - \boldsymbol{H}\,\boldsymbol{f} \right)\|^{2} \right\rangle_{q_{1-j}({f}_{j})} \\ & -\frac{1}{2} \left\langle \| \left({\widetilde{\boldsymbol{V}_{\boldsymbol{f}}^{-1}}}\right)^{\frac{1}{2}} \boldsymbol{f} \|^{2} \right\rangle_{q_{1-j}({f}_{j})} \end{aligned}  $$

For the first norm, considering all the *f*_*j*_ free terms as constants, we have:

(72) $${} \begin{aligned} \|\left({\widetilde{\boldsymbol{V}_{\boldsymbol{\epsilon}}^{-1}}}\right)^{1/2} \left(\boldsymbol{g} - \boldsymbol{H}\,\boldsymbol{f} \right)\|^{2} &= C + \| \left({\widetilde{\boldsymbol{V}_{\boldsymbol{\epsilon}}^{-1}}}\right)^{1/2} \boldsymbol{H}^{j}\|^{2}{f}_{j}^{2} \\&\quad- 2 \boldsymbol{H}^{{j} T} {\widetilde{\boldsymbol{V}_{\boldsymbol{\epsilon}}^{-1}}} \left(\boldsymbol{g} - \boldsymbol{H}^{-{j}}\boldsymbol{f}^{-{j}} \right){f}_{j} \end{aligned}   $$

where ***H*** ^*j*^ represents the column *j* of the matrix ***H***, ***H*** ^−*j*^ represents the matrix ***H*** except the column *j*, and ***f*** ^−*j*^ represents the vector ***f*** except the element *f*_*j*_. Introducing the notation

(73) $${} {\widetilde{{f}_{k}}} = \int {f}_{k} q_{1k}({f}_{k})\, \text{d} {f}_{k} \;\; ; \;\;{\widetilde{\boldsymbol{f}^{-{j}}}} = \left[ {\widetilde{{f}_{1}}} \; \ldots \; {\widetilde{{f}_{j-1}}} \; {\widetilde{{f}_{j+1}}} \; \ldots \; {\widetilde{z_{M}}} \right]^{T}   $$

the expectancy of the first norm becomes:

(74) $${} {\fontsize{8.8pt}{9.6pt}{\begin{aligned} \left\langle \|\left({\widetilde{\boldsymbol{V}_{\boldsymbol{\epsilon}}^{-1}}}\right)^{1/2} \left(\boldsymbol{g} - \boldsymbol{H}\,\boldsymbol{f}\right)\|^{2} \right\rangle_{q_{1-j}({f}_{j})} &= C + \| \left({\widetilde{\boldsymbol{V}_{\boldsymbol{\epsilon}}^{-1}}}\right)^{1/2} \boldsymbol{H}^{j}\|^{2}{f}_{j}^{2}\\ &\quad - 2 \boldsymbol{H}^{{j} T} {\widetilde{\boldsymbol{V}_{\boldsymbol{\epsilon}}^{-1}}} \left(\boldsymbol{g} - \boldsymbol{H}^{-{j}}{\widetilde{\boldsymbol{f}^{-{j}}}}\right)f_{j} \end{aligned}}}   $$

The expectancy for the second norm, considering all the free *f*_*j*_ terms as constants:

(75) $$ \left\langle \| \left({\widetilde{\boldsymbol{V}_{\boldsymbol{f}}^{-1}}}\right)^{\frac{1}{2}} \boldsymbol{f} \|^{2} \right\rangle_{q_{1-j}({f}_{j})} = C + {\widetilde{{v}_{{f}_{j}}^{-1}}} {f}_{j}^{2}   $$

From Eqs. (31) and (71) and Eqs. (74) and (75), the proportionality for *q*_1*j*_(*f*_*j*_) becomes:

(76) $${} \begin{aligned} q_{1j}({f}_{j}) \propto &\exp \left\{\left(\|\left({\widetilde{\boldsymbol{V}_{\boldsymbol{\epsilon}}^{-1}}}\right)^{1/2} \boldsymbol{H}^{j}\|^{2} + {\widetilde{v_{f_{j}}^{-1}}} \right){f}_{j}^{2}\right. \\&\qquad \left.- 2 \boldsymbol{H}^{{j} T} {\widetilde{\boldsymbol{V}_{\boldsymbol{\epsilon}}^{-1}}} \left(\boldsymbol{g} - \boldsymbol{H}^{-{j}}\,{\widetilde{\boldsymbol{f}^{-{j}}}} \right)f_{j} \right\} \end{aligned}  $$

Defining the criterion $J\left ({f}_{j}\right) = \left (\| \left ({\widetilde {\boldsymbol {V}_{\boldsymbol {\epsilon }}^{-1}}}\right)^{1/2} \boldsymbol {H}^{j}\|^{2} + {\widetilde {v_{f_{j}}^{-1}}} \right) {f}_{j}^{2} - 2 \boldsymbol {H}^{{j} T} {\widetilde {\boldsymbol {V}_{\boldsymbol {\epsilon }}^{-1}}} \left (\boldsymbol {g} - \boldsymbol {H}^{-{j}}\,{\widetilde {\boldsymbol {f}^{-{j}}}} \right)f_{j}$, we arrive to the following.

##### Intermediate conclusion 4.

The probability distribution function *q*_1*j*_(*f*_*j*_) is a normal distribution.

In order to compute the mean of the normal distribution, it is sufficient to compute the solution that minimizes the criterion *J*(*f*_*j*_):

(77) $$ \frac{\partial J({f}_{j})}{\partial {f}_{j}} = 0 \Leftrightarrow {\widehat{{f}_{j}}}_{\text{PM}} = \frac{\boldsymbol{H}^{{j} T} {\widetilde{\boldsymbol{V}_{\boldsymbol{\epsilon}}^{-1}}} \left(\boldsymbol{g} - \boldsymbol{H}^{-{j}}\,{\widetilde{\boldsymbol{f}^{-j}}} \right)}{\|\left({\widetilde{\boldsymbol{V}_{\boldsymbol{\epsilon}}^{-1}}}\right)^{1/2} \boldsymbol{H}^{j}\|^{2} + {\widetilde{v_{f}^{-1}}}}   $$

For the variance, we apply the same identification strategy as in the previous case, obtaining:

(78) $$ q_{1}({f}_{j}) = \mathcal{N}\left({f}_{j} | {\widehat{{f}_{j}}}_{\text{PM}}, \text{var}_{j} \right), \left\{ \begin{array}{l} {\widehat{{f}_{j}}}_{\text{PM}} = \frac{\boldsymbol{H}^{{j} T} {\widetilde{\boldsymbol{V}_{\boldsymbol{\epsilon}}^{-1}}} \left(\boldsymbol{g} - \boldsymbol{H}^{-{j}}{\widetilde{\boldsymbol{f}^{-j}}}\right)}{\| \left({\widetilde{\boldsymbol{V}_{\boldsymbol{\epsilon}}^{-1}}}\right)^{1/2} \boldsymbol{H}^{j}\|^{2} + {\widetilde{v_{f_{j}}^{-1}}}} \\ \text{var}_{j} = \frac{1}{\| \left({\widetilde{\boldsymbol{V}_{\boldsymbol{\epsilon}}^{-1}}}\right)^{1/2} \boldsymbol{H}^{j}\|^{2} + {\widetilde{v_{{f}_{j}}^{-1}}}} \end{array}\right.   $$

**∙ Expression of** $\phantom {\dot {i}\!}\boldsymbol {q_{2i}({v}_{{\epsilon }_{i}})}$The proportionality relation corresponding to $\phantom {\dot {i}\!}q_{2i}\left (v_{{\epsilon }_{i}}\right)$ established in Eq. (31) refers to $\phantom {\dot {i}\!}v_{{\epsilon }_{i}}$, so in the expression of ln*p* (***f***,***v***_***ε***_,***v***_***f***_|***g***), all the terms free of $\phantom {\dot {i}\!}v_{{\epsilon }_{i}}$ can be regarded as constants:

(79) $$ \begin{aligned} \ln p\!\left(\boldsymbol{f},\boldsymbol{v}_{\boldsymbol{\epsilon}},\boldsymbol{v}_{\boldsymbol{f}}|\boldsymbol{g}\right) = C &-\left(\alpha_{\epsilon 0} + 1 + \frac{1}{2}\right) \ln {v}_{{\epsilon}_{i}} \\&-\left(\beta_{\epsilon 0} + \frac{1}{2} \left({g}_{i} - \boldsymbol{H}_{i}\,\boldsymbol{f}\right)\right) {v}_{{\epsilon}_{i}}^{-1} \end{aligned}  $$

With the notation:

(80) $$ \left\langle \boldsymbol{f} \right\rangle_{q_{1}(\boldsymbol{f})} = \left[ {\widehat{{f}_{1}}}_{\text{PM}} \ldots {\widehat{{f}_{j}}}_{\text{PM}} \ldots {\widehat{{f}_{M}}}_{\text{PM}} \right]^{T} \stackrel{Not}{=} {\widehat{\boldsymbol{f}}}_{\text{PM}}   $$

the expectancy of the logarithm becomes:

(81) $$ \begin{aligned} &\left\langle \ln p\!\left(\boldsymbol{f},\boldsymbol{v}_{\boldsymbol{\epsilon}},\boldsymbol{v}_{\boldsymbol{f}}|\boldsymbol{g}\right) \right\rangle_{q_{1}(\boldsymbol{f}) \; q_{2-i}\left({v}_{{\epsilon}_{i}}\right) \; q_{3}(\boldsymbol{v}_{\boldsymbol{f}})} \\&= C -\left(\alpha_{\epsilon 0} + 1 + \frac{1}{2}\right) \ln {v}_{{\epsilon}_{i}} \\ & \qquad -\left(\beta_{\epsilon 0} + \frac{1}{2} \left[\boldsymbol{H}_{i} {\widehat{\boldsymbol{\Sigma}}} \boldsymbol{H}_{i}^{T} + \left({g}_{i} - \boldsymbol{H}_{i} \,{\widehat{\boldsymbol{f}}}_{\text{PM}} \right)^{2}\right] \right) {v}_{{\epsilon}_{i}}^{-1} \end{aligned}  $$

and the proportionality relation for $q_{2i}\left ({v}_{{\epsilon }_{i}}\right)$ becomes:

(82) $${} \begin{aligned} q_{2i}\left({v}_{{\epsilon}_{i}}\right) \propto {v}_{{\epsilon}_{i}}^{-\left(\alpha_{\epsilon 0} + 1 + \frac{1}{2}\right)} &\exp \left\{ -\left(\beta_{\epsilon 0} + \frac{1}{2} \left[\boldsymbol{H}_{i} {\widehat{\boldsymbol{\Sigma}}} \boldsymbol{H}_{i}^{T}\right.\right.\right. \\&\qquad + \left.\left.\left. \left({g}_{i} - \boldsymbol{H}_{i} \,{\widehat{\boldsymbol{f}}}_{\text{PM}} \right)^{2}\right] \right) {v}_{{\epsilon}_{i}}^{-1} \right\} \end{aligned}   $$

Equation 82 leads to the following.

##### Intermediate conclusion 5.

The probability distribution function $q_{2i}\left ({v}_{{\epsilon }_{i}}\right)$ is an inverse gamma distribution, with the parameters $\alpha _{\epsilon _{i}}$ and $\beta _{\epsilon _{i}}$.

[]

(83) $${\kern15pt} q_{2i}\left(v_{{\epsilon}_{i}}\right) = \mathcal{I}\mathcal{G} \left(v_{{\epsilon}_{i}}|\alpha_{\epsilon_{i}},\beta_{\epsilon_{i}}\right), \left\{ \begin{array}{l} \alpha_{\epsilon_{i}} = \alpha_{\epsilon 0} + \frac{1}{2} \\ \beta_{\epsilon_{i}} = \beta_{\epsilon 0} + \frac{1}{2} \left[\boldsymbol{H}_{i} {\widehat{\boldsymbol{\Sigma}}} \boldsymbol{H}_{i}^{T} + \left({g}_{i} - \boldsymbol{H}_{i} \,{\widehat{\boldsymbol{f}}}_{\text{PM}} \right)^{2}\right] \end{array}\right.   $$

**∙ Expression of** $\phantom {\dot {i}\!}\boldsymbol {q_{3j}({v}_{{f}_{j}})}$The proportionality relation corresponding to $\phantom {\dot {i}\!}q_{3j}\left (v_{f_{j}}\right)$ established in Eq. (31) refers to $\phantom {\dot {i}\!}v_{{f}_{j}}$, so in the expression of ln*p* (***f***,***v***_***ε***_,***v***_***f***_|***g***), all the terms free of $v_{f_{j}}$ can be regarded as constants:

(84) $${} \begin{aligned} \ln p\!\left(\boldsymbol{f},\boldsymbol{v}_{\boldsymbol{\epsilon}},\boldsymbol{v}_{\boldsymbol{f}}|\boldsymbol{g}\right) = C &-\frac{1}{2} \ln {v}_{{f}_{j}} -\frac{1}{2} \left\langle {f}_{j}^{2} \right\rangle_{q_{1j}({f}_{j})} {v}_{{f}_{j}}^{-1}\\ &-\left(\alpha_{f_{j} 0} + 1 \right) \ln {v}_{{f}_{j}} - \beta_{f_{j} 0} {v}_{{f}_{j}}^{-1} \end{aligned}  $$

The integral of the logarithm:

(85) $${} {\fontsize{8.4pt}{9.6pt}{\begin{aligned} \left\langle \ln p\!\left(\boldsymbol{f},\boldsymbol{v}_{\boldsymbol{\epsilon}},\boldsymbol{v}_{\boldsymbol{f}}|\boldsymbol{g}\right) \right\rangle_{q_{1}(\boldsymbol{f}) \; q_{2}(\boldsymbol{v}_{\boldsymbol{\epsilon}}) \; q_{3-j}\left(v_{f_{j}}\right)} &= C -\left(\alpha_{f 0} + \frac{1}{2} + 1 \right)\ln {v}_{{f}_{j}} \\ &\quad-\left[\!\beta_{f 0} + \frac{1}{2} \left({\widehat{{f}_{j}}}_{\text{PM}}^{2} + \text{var}_{j}\right)\!\right]{v}_{{f}_{j}}^{-1} \end{aligned}}}   $$

Equation 85 leads to the following.

##### Intermediate conclusion 6.

The probability distribution function $q_{3j}\left ({v}_{{f}_{j}}\right)$ is an inverse gamma distribution, with the parameters $\alpha _{f_{j}}$ and $\beta _{f_{j}}$.

(86) $${} q_{3j}\left({v}_{{f}_{j}}\right) = \mathcal{I}\mathcal{G} \left({v}_{{f}_{j}}|\alpha_{f_{j}}, \beta_{f_{j}}\right), \left\{ \begin{array}{l} \alpha_{f_{j}} = \alpha_{f 0} + \frac{1}{2} \\ \beta_{f_{j}} = \beta_{f 0} + \frac{1}{2} \left({\widehat{{f}_{j}}}_{\text{PM}}^{2} + \text{var}_{j}\right) \end{array}\right.   $$

### Appendix 4

#### List of symbols and abbreviations

*List of symbols*

During the article, all the terms written in bold represent vectors or matrices.

1. ***H***—the matrix used in the linear model considered during all the article. $\boldsymbol {H} \in \mathcal {M}_{N\times M}$. The matrix corresponds to the IFT and can be derived from Eq. (2).
2. ***H***_*i*_ represents the i line of the matrix ***H***. $\boldsymbol {H}_{i} \in \mathcal {M}_{1\times M}$
3. ***g***_0_ represents the “theoretical” signal, i.e., the signal corresponding to the considered model (2) that does not account for the noise, ***g***_0_=***H*** ***f***. During the synthetic simulation section, the comparison between the estimated signal ${\widehat {\boldsymbol {g}_{0}}}$ and the theoretical signal ***g***_0_ is particular important, measuring if the propose algorithm selects the solution corresponding to the biological phenomena.
4. ***f*** represents the PC vector, $\boldsymbol {f} \in \mathcal {M}_{1\times M}$. This is the fundamental unknown of our model. All the estimates of the PC vector are denoted ${\widehat {\boldsymbol {f}}}$ and in specific cases the particular estimation used in the model is indicated: ${\widehat {\boldsymbol {f}}_{\textit {JMAP}}}$ or ${\widehat {\boldsymbol {f}}_{\text {PM}}}$. During the article, the subscript used for indicating an element of the PC vector is *i*: *f*_*i*_ and the element is not bold, being a scalar.
5. ***ε*** represents the errors: $\boldsymbol {\epsilon } = \left [{\epsilon }_{1}, {\epsilon }_{2}, \ldots, {\epsilon }_{N}\right ]^{T} \in \mathcal {M}_{N \times 1},$ is an *N*-dimensional vector

*List of abbreviations*

1. CT—circadian time
2. CTS—circadian timing system
3. FFT—fast Fourier transform
4. IGSM—infinite Gaussian scale mixture
5. IP—inverse problem
6. JMAP—joint maximum a posteriori
7. KL—Kullback-Leibler
8. PC vector—periodic component vector
9. PM—posterior mean
10. RT-BIO—RealTime Biolumicorder
11. TSVD—truncated single value decomposition
12. TRM—Tikhonov regularization methods
13. VBA—variational Bayesian approximation
14. ZT—Zeitgeber time

## Additional file

Additional file 1
**Synthetic case simulations: 10 dB and 15 dB** (PDF 309 kb)
