# Supplementary material for: Precise periodic components estimation for chronobiological signals through Bayesian Inference with sparsity enforcing prior
Source: EURASIP J Bioinform Syst Biol. 2016 Jan 20;2016:3. doi: 10.1186/s13637-015-0033-6 (PMC4720710; doi:10.1186/s13637-015-0033-6)
Supplement: Additional file 1 — Synthetic case simulations: 10 dB and 15 dB (PDF 309 kb) [file 13637_2015_33_MOESM1_ESM.pdf]

Precise periodic components estimation for chronobiological signals through Bayesian Inference with sparsity enforcing prior Supplementary Material

Mircea Dumitru[1,2], Ali Mohammad-Djafari[1], Simona Baghai Sain[1,3]

[1]*Laboratoire des signaux et systèmes (L2S), UMR 8506 CNRS–CentraleSupélec–Univ. Paris-Sud, CentraleSupélec, Plateau de Moulon, 91192 Gif-sur-Yvette, France*

[2]*Rythmes Biologiques et Cancers (RBC), UMR 776 INSERM–Univ. Paris-Sud, Campus CNRS, 94801 Villejuif, France*

[3]*Department of Molecular Biotechnology and Health Sciences, University of Turin, 10126, Turin, Italy*

1 Supplementary Material

In this supplementary material we present the simulations corresponding to the synthetic case, for two levels of noise: 10dB and 15dB. The protocol is the same as the one presented in the main article.

2 Synthetic data 10dB

In this subsection we present the synthetic data case corresponding to the 10 dB SNR.

2.1 Data 10dB

The periodic component vector $\boldsymbol{f}$, theoretical signal $\boldsymbol{g}_0$ and the signal $\boldsymbol{g}$ are presented in the following figure:

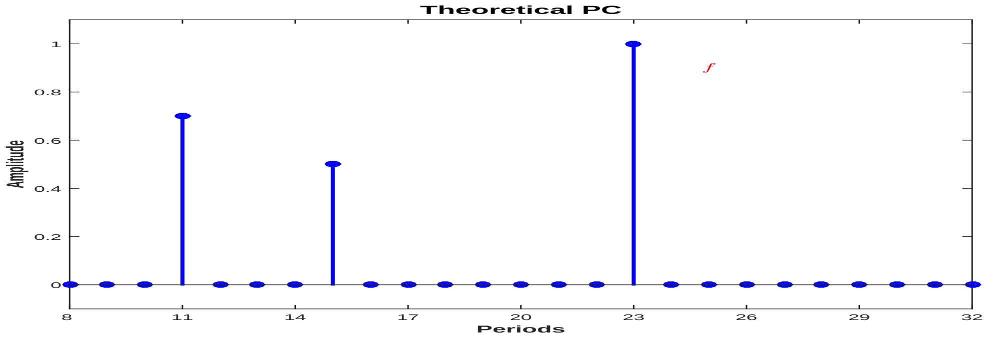

(a) PC vector $\boldsymbol{f}$

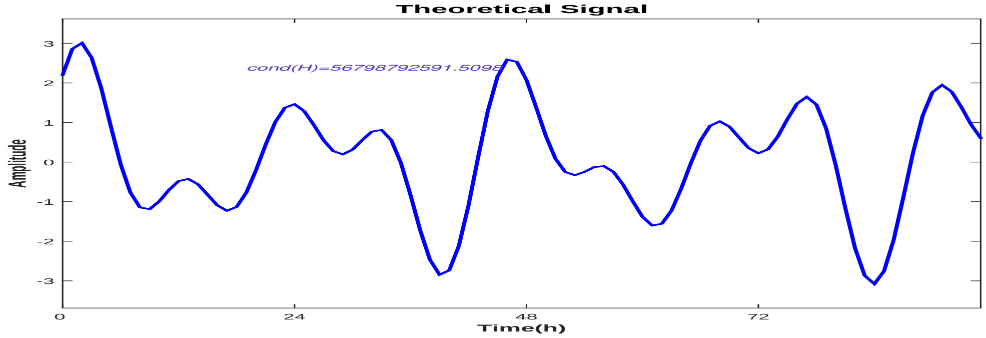

(b) Theoretical Signal $\boldsymbol{g}_0$

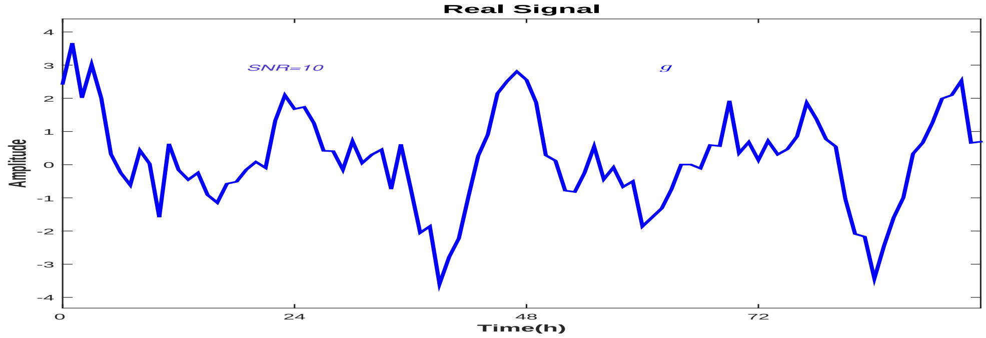

(c) Signal $\boldsymbol{g}$

Figure 1: $\boldsymbol{f}$ PC vector, theoretical signal $\boldsymbol{g}_0$ and input signal $\boldsymbol{g} = \boldsymbol{g}_0 + \boldsymbol{\epsilon}$ of the model (10dB)

Figure (1a) shows the theoretical PC, set for 11h, 15h and 23h. All the other values in the PC vector are zero. Figure (1b) presents the signal corresponding to the linear model considered in Equation (3), neglecting the errors, $\boldsymbol{g}_0 = \boldsymbol{H}\boldsymbol{f}$. All the simulations are done using as the input the noisy signal $\boldsymbol{g}$, (1c) corresponding to the linear model,Equation (3).

2.2 JMAP IGSM 10dB

A comparison between the the synthetic data and the JMAP IGSM estimation is presented in the Figure (2). The theoretical periodic component vector $\boldsymbol{f}$ and the JMAP IGSM estimation $\widehat{\boldsymbol{f}}_{JMAP}$ are compared in Figure (2a). The

reconstruction error is $\delta \boldsymbol{f} = \frac{\|\boldsymbol{f}-\widehat{\boldsymbol{f}}_{JMAP}\|_2^2}{\|\boldsymbol{f}\|_2^2} = 0.0259$. The comparison between the estimated $\widehat{\boldsymbol{g}}_{JMAP}$ and the theoretical signal (without noise) $\boldsymbol{g}_0$ is presented in in Figure (2b). The reconstruction error is $\delta \boldsymbol{g}_0 = \frac{\|\boldsymbol{g}_0-\widehat{\boldsymbol{g}}_{JMAP}\|_2^2}{\|\boldsymbol{g}_0\|_2^2} = 0.0225$. The comparison between the estimated $\widehat{\boldsymbol{g}}_{JMAP}$ and the signal $\boldsymbol{g}$ is presented in in Figure (2c).

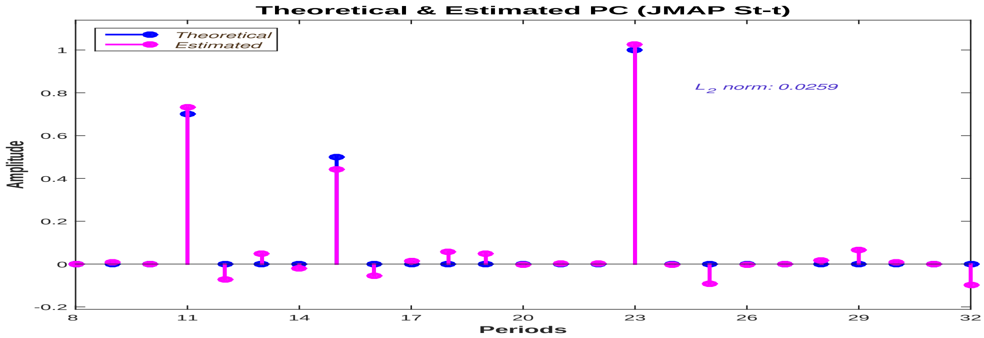

(a) Theoretical and Estimated PC

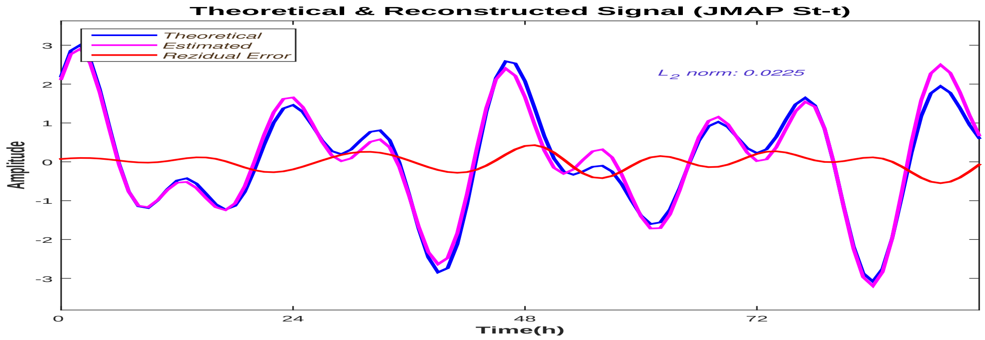

(b) Theoretical and Estimated Signal

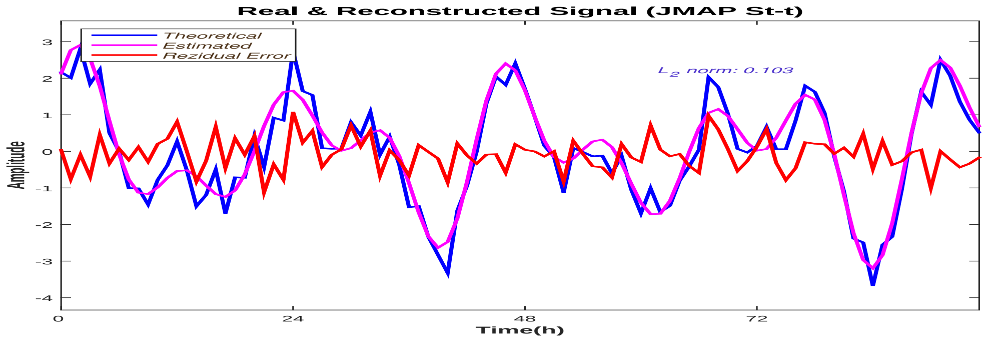

(c) Real and Estimated Signal

Figure 2: JMAP IGSM Estimation (10dB)

Figure (3a) presents the variation of L_2 PC vector error reconstruction for 10 different noise realisation, showing high variations. Important variations corresponding to the L_2 error reconstruction for the theoretical signal $\boldsymbol{g}_0$, and signal $\boldsymbol{g}$, are presented in Figure (3b) and Figure (3c).

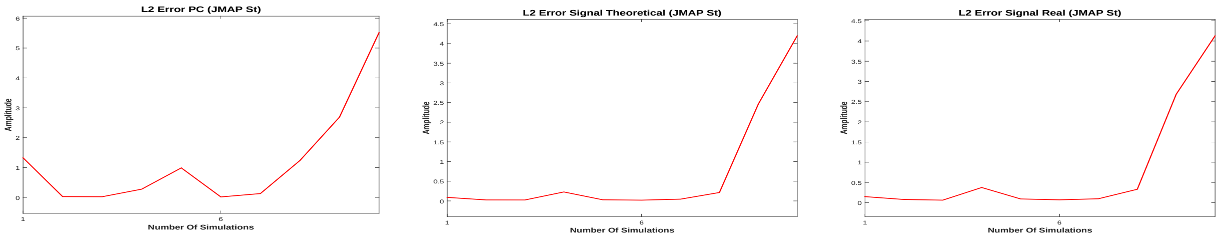

(a) L_2 Error: $\boldsymbol{f}$ vs. $\widehat{\boldsymbol{f}}_{JMAP}$ (b) L_2 Error: $\boldsymbol{g}_0$ vs. $\widehat{\boldsymbol{g}}_{JMAP}$ (c) L_2 Error: $\boldsymbol{g}$ vs. $\widehat{\boldsymbol{g}}_{JMAP}$

Figure 3: JMAP IGSM L_2 error measured for 10 different noise realisations (10dB)

2.3 PM (via VBA, partial separability) IGSM 10dB

For the Posterior Mean estimation via VBA, both the PC estimation and theoretical signal $\boldsymbol{g}_0$ reconstruction are very accurate, Figure (4a) and Figure (4b). For the reconstruction of the theoretical signal $\boldsymbol{g}_0$, the L_2 error norm is $\delta\boldsymbol{g}_0 = \frac{\|\boldsymbol{g}_0 - \widehat{\boldsymbol{g}}_{PM}\|_2^2}{\|\boldsymbol{g}_0\|_2^2} = 0.011$. For the PC vector, the reconstruction error is $\delta\boldsymbol{f} = \frac{\|\boldsymbol{f} - \widehat{\boldsymbol{f}}_{PM}\|_2^2}{\|\boldsymbol{f}\|_2^2} = 0.0144$. The algorithm is converging to a sparse solution where all the non zero clocks are detected. The residual error computed between $\boldsymbol{g}$ and the reconstructed signal is consistent with the error considered in the model, 10dB, Figure (4c).

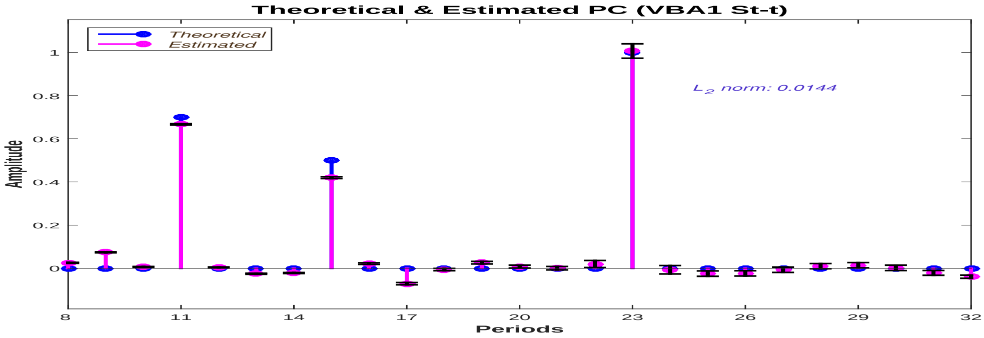

(a) Theoretical and Estimated PC

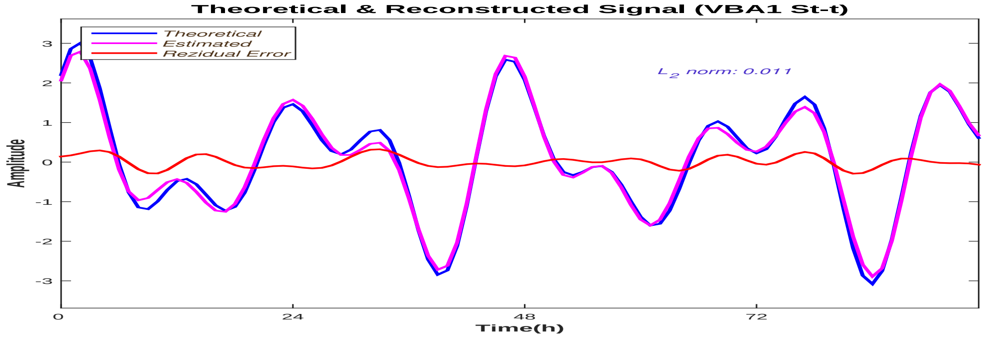

(b) Theoretical and Estimated Signal

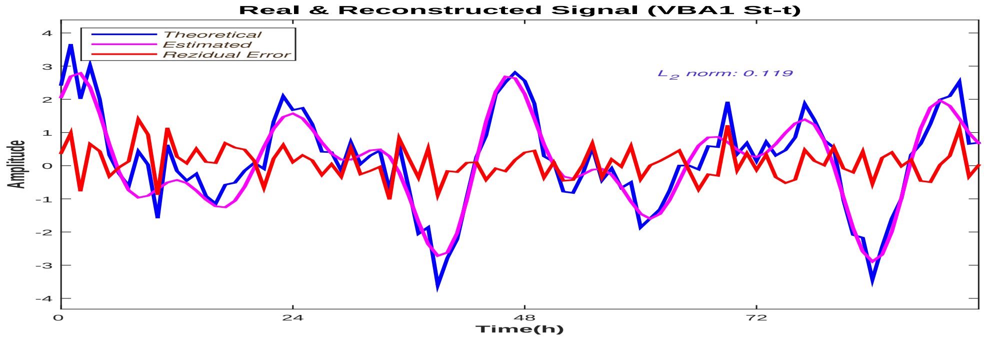

(c) Real and Estimated Signal

Figure 4: PM (via VBA, partial separability) IGSM Estimation (10dB)

The convergency of $\mathbf{\Sigma}$ covariance matrix diagonal, Figure (5a) and the convergency of algorithm's solution $\boldsymbol{f}$, Figure (5b) is showing a very fast convergency. All the estimates are superposed after the first ten iterations.

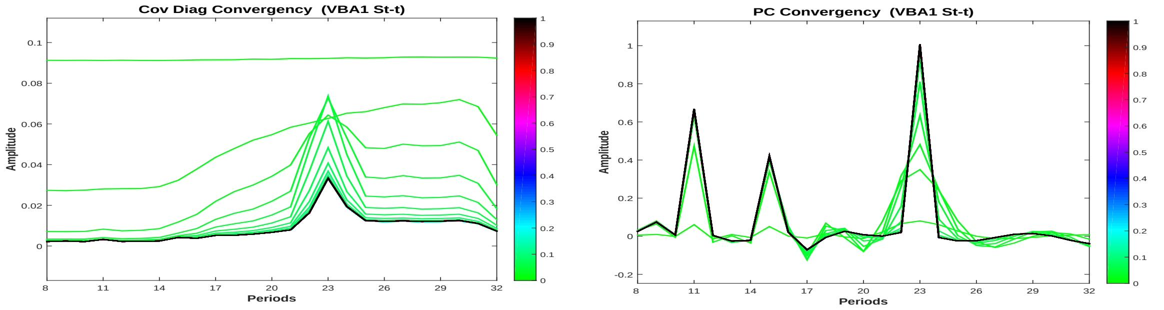

(a) diag($\widehat{\boldsymbol{\Sigma}}$) convergency

(b) $\boldsymbol{f}$ convergency

Figure 5: PM (via VBA, partial separability) IGSM hyperparameters and $\boldsymbol{f}$ convergency

We show that for the PM estimation, the error variation is very small. Figure (6a) presents the variation of L_2 PC vector error reconstruction for 10 different noise realisation. The figure presents a very small variation of L_2 PC vector error reconstruction, between 0.008 and 0.02. Very small variations corresponding to the L_2 error reconstruction for the theoretical signal $\boldsymbol{g}_0$, and signal $\boldsymbol{g}$, are presented in Figure (6b) and Figure (6c).

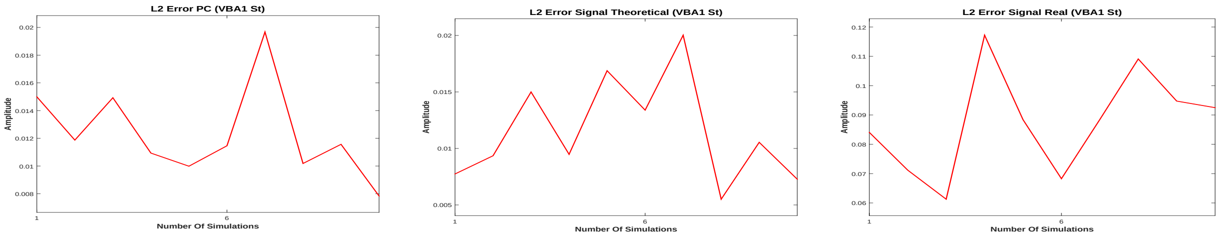

(a) L_2 Error: $\boldsymbol{f}$ vs. $\widehat{\boldsymbol{f}}_{PM}$

(b) L_2 Error: $\boldsymbol{g}_0$ vs. $\widehat{\boldsymbol{g}}_{PM}$

(c) L_2 Error: $\boldsymbol{g}$ vs. $\widehat{\boldsymbol{g}}_{PM}$

Figure 6: PM (via VBA, partial separability) IGSM L_2 error measured for 10 different noise realisations (10dB)

2.4 PM (via VBA, full separability) IGSM 10dB

The estimations for the full separability case are also accurate:

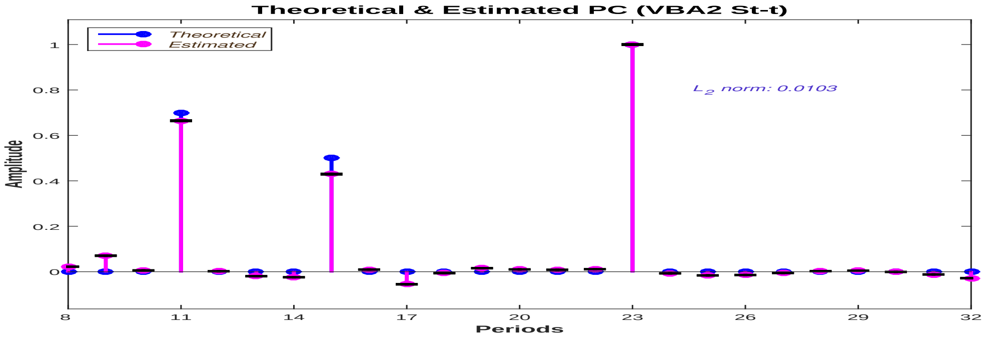

(a) Theoretical and Estimated PC

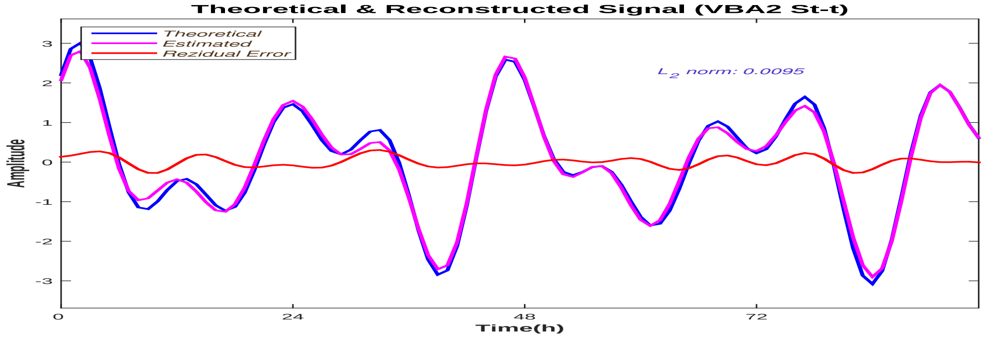

(b) Theoretical and Estimated Signal

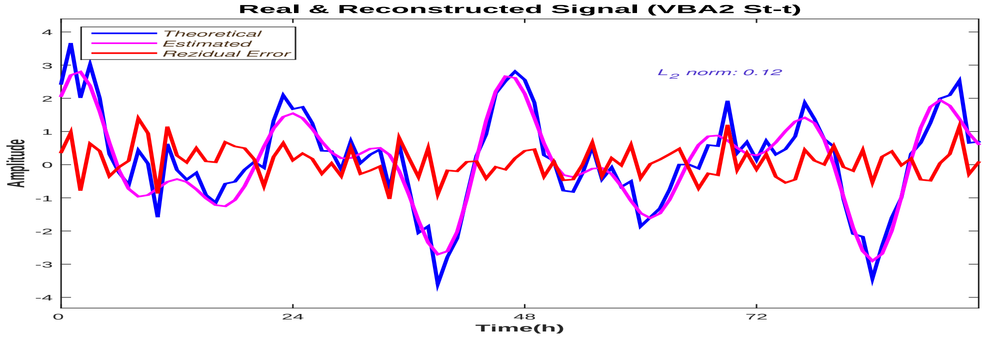

(c) Real and Estimated Signal

Figure 7: PM (via VBA, full separability) IGSM Estimation (10dB)

For the reconstruction of the theoretical signal $\boldsymbol{g}_0$, the L_2 error norm is $\delta \boldsymbol{g}_0 = \frac{\|\boldsymbol{g}_0 - \widehat{\boldsymbol{g}}_{PM}\|_2^2}{\|\boldsymbol{g}_0\|_2^2} = 0.0095$. For the PC vector, the reconstruction error is $\delta \boldsymbol{f} = \frac{\|\boldsymbol{f} - \widehat{\boldsymbol{f}}_{PM}\|_2^2}{\|\boldsymbol{f}\|_2^2} = 0.0103$.

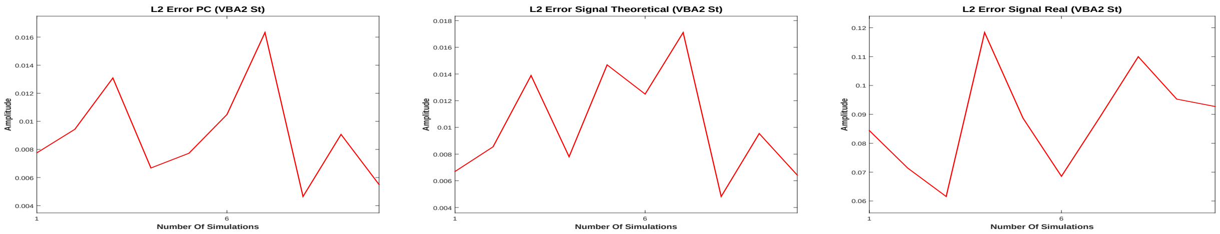

(a) Theoretical and Estimated PC (b) Theoretical and Estimated Signal (c) Real and Estimated Signal

Figure 8: PM (via VBA, full separability) IGSM L_2 error measured for 10 different noise realisations (10dB)

Figure (8a) presents the variation of L_2 PC vector error reconstruction for 10 different noise realisation. The figure presents a very small variation of L_2 PC vector error reconstruction, between 0.0046 and 0.0168. Very small variations corresponding to the L_2 error reconstruction for the theoretical signal $\boldsymbol{g}_0$, and signal $\boldsymbol{g}$, are presented in Figure (8b) and Figure (8c).

2.5 Methods comparison 10dB

The comparison between the estimations corresponding to the the proposed IGSM models is presented in Figure (9c) (JMAP estimator), Figure (9d) (PM via VBA, partial separability estimator) and Figure (9e) (PM via VBA, full separability estimator). The comparison with the Gaussian case (i.e. Gaussian prior), via the two estimators discussed, is presented in Figure (9a) (Gaussian Model, JMAP estimator) and Figure (9b) (PM via VBA estimator). A comparison with the FFT is presented in Figure (9f).

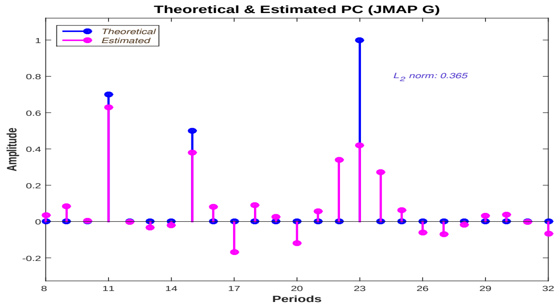

(a) JMAP Gaussian

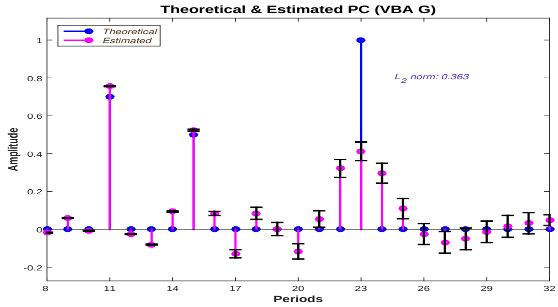

(b) PM (via VBA) Gaussian

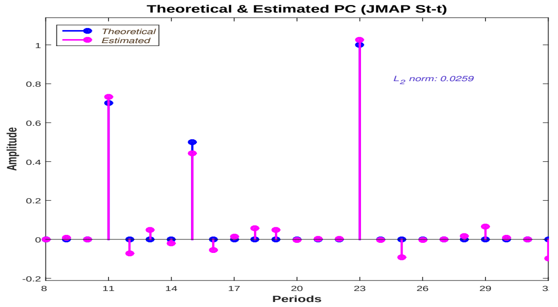

(c) JMAP IGSM

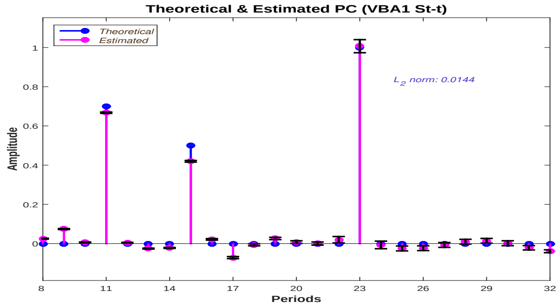

(d) PM (via VBA p. s.) IGSM

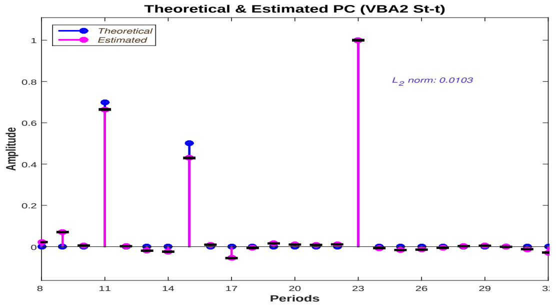

(e) PM (via VBA f. s.) IGSM

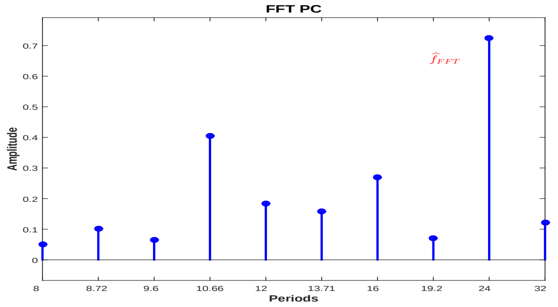

(f) FFT

Figure 9: Methods Comparison (10dB)

The L_2 estimation error for the PC vector is very high for the two Gaussian models. Also, the estimations are not sparse. For the IGSM models, the JMAP estimator is providing a good estimation, but it is unstable. VBA estimator, both partial separable and fully separable provides very accurate stable estimations.

2.6 Error comparison 10dB

The L_2 error measurement corresponding to the PC estimation, theoretical signal estimation and the signal estimation, for 10 different noise realisation is presented in the following figure:

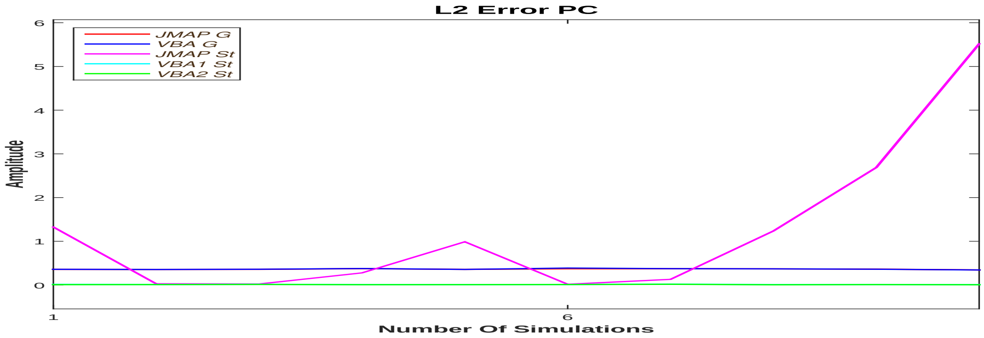

(a) PC error estimation

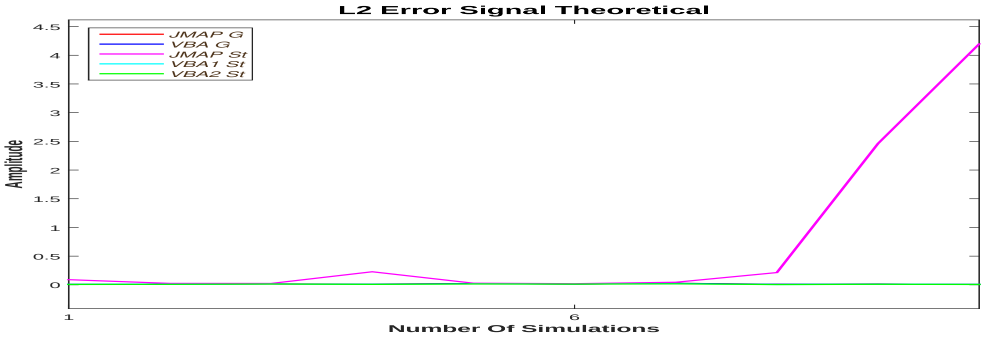

(b) Theoretical signal error estimation

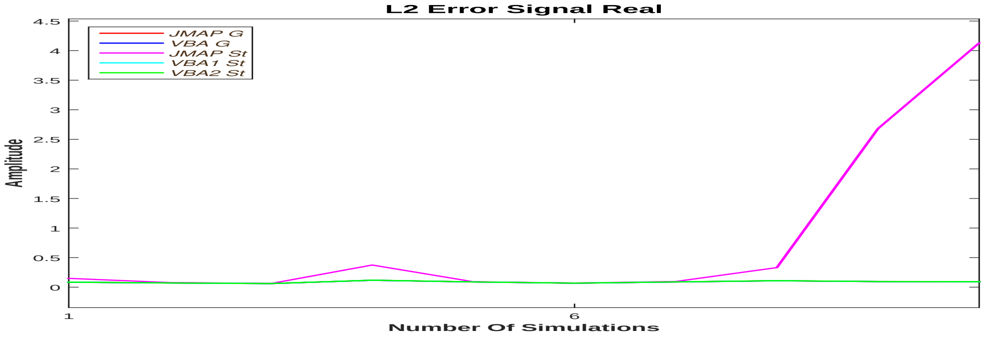

(c) Signal error estimation (10dB)

Figure 10: L_2 Errors estimation (10dB)

The $L2$ error corresponding to the PM via VBA IGSM Model shows the performances of proposed via algorithm compared to the Gaussian Model and the JMAP estimation for IGSM Model.

3 Synthetic data 15dB

In this subsection we present the synthetic data case corresponding to the 15 dB SNR.

3.1 Data 15dB

The periodic component vector $\boldsymbol{f}$, theoretical signal $\boldsymbol{g}_0$ and the signal $\boldsymbol{g}$ are presented in the following figure:

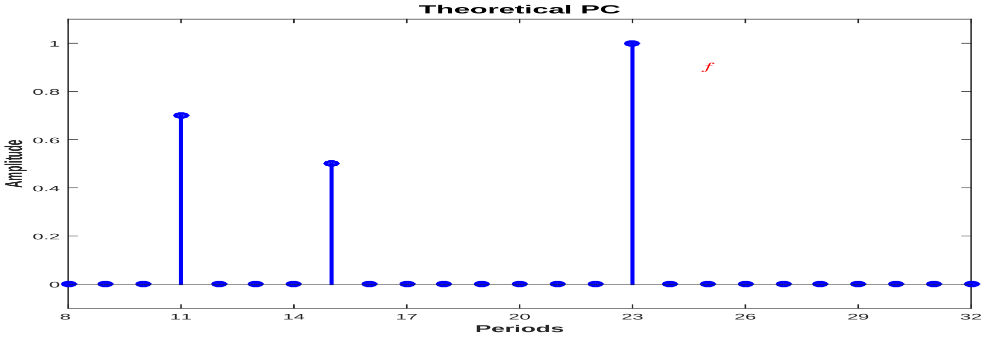

(a) PC vector $\boldsymbol{f}$

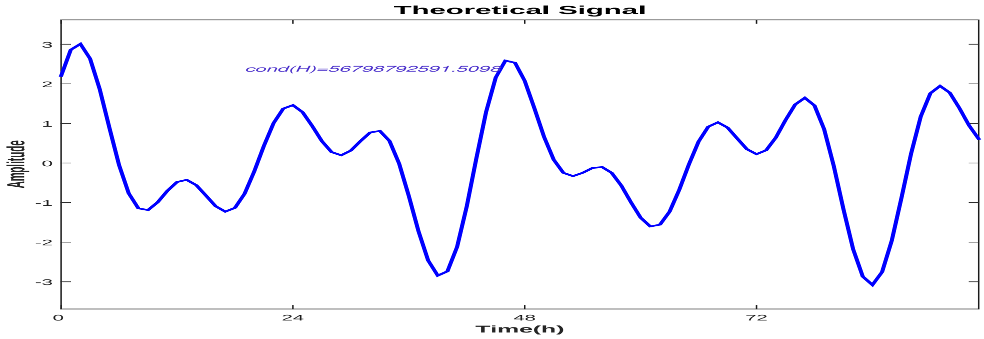

(b) Theoretical Signal $\boldsymbol{g}_0$

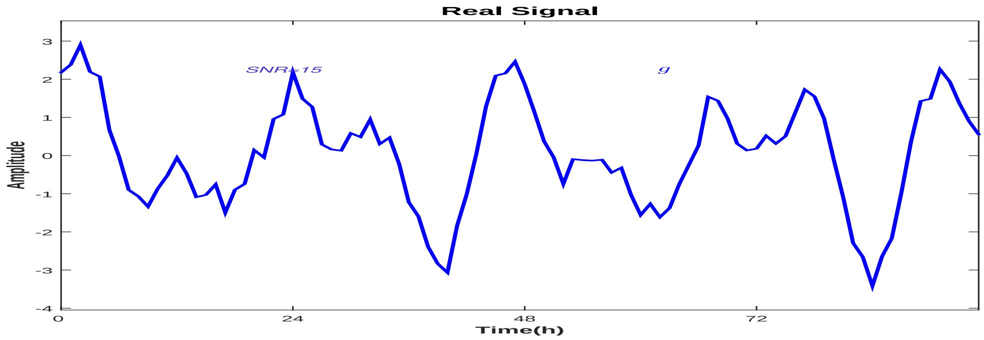

(c) Signal $\boldsymbol{g}$

Figure 11: $\boldsymbol{f}$ PC vector, theoretical signal $\boldsymbol{g}_0$ and input signal $\boldsymbol{g} = \boldsymbol{g}_0 + \boldsymbol{\epsilon}$ of the model (15dB)

Figure (11a) shows the theoretical PC, set for 11h, 15h and 23h. All the other values in the PC vector are zero. Figure (11b) presents the signal corresponding to the linear model considered in Equation (3), neglecting the errors, $\boldsymbol{g}_0 = \boldsymbol{H}\boldsymbol{f}$. All the simulations are done using as the input the noisy signal $\boldsymbol{g}$, (11c) corresponding to the linear model,Equation (3).

3.2 JMAP IGSM 15dB

A comparison between the the synthetic data and the JMAP IGSM estimation is presented in the Figure (12). The theoretical periodic component vector $\boldsymbol{f}$ and the JMAP IGSM estimation $\widehat{\boldsymbol{f}}_{JMAP}$ are compared in Figure (12a). The reconstruction error is $\delta \boldsymbol{f} = \frac{\|\boldsymbol{f}-\widehat{\boldsymbol{f}}_{JMAP}\|_2^2}{\|\boldsymbol{f}\|_2^2} = 0.0069$. The comparison between the estimated $\widehat{\boldsymbol{g}}_{JMAP}$ and the theoretical signal (without noise) $\boldsymbol{g}_0$ is presented in in Figure (12b). The reconstruction error is $\delta \boldsymbol{g}_0 = \frac{\|\boldsymbol{g}_0-\widehat{\boldsymbol{g}}_{JMAP}\|_2^2}{\|\boldsymbol{g}_0\|_2^2} = 0.00692$. The comparison between the estimated $\widehat{\boldsymbol{g}}_{JMAP}$ and the signal $\boldsymbol{g}$ is presented in in Figure (12c).

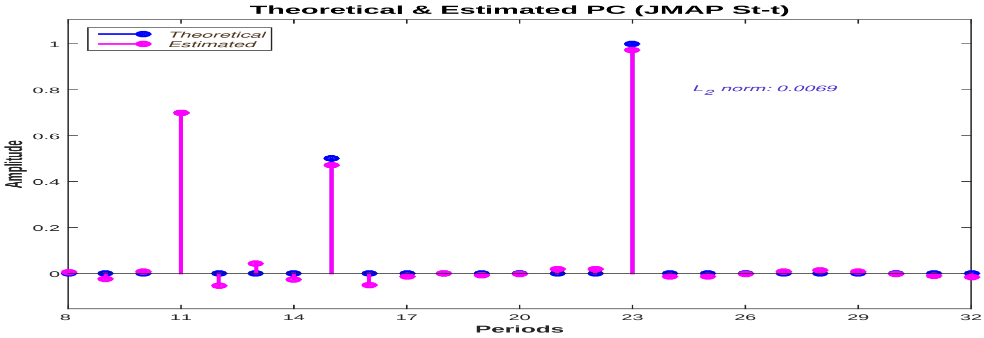

(a) Theoretical and Estimated PC

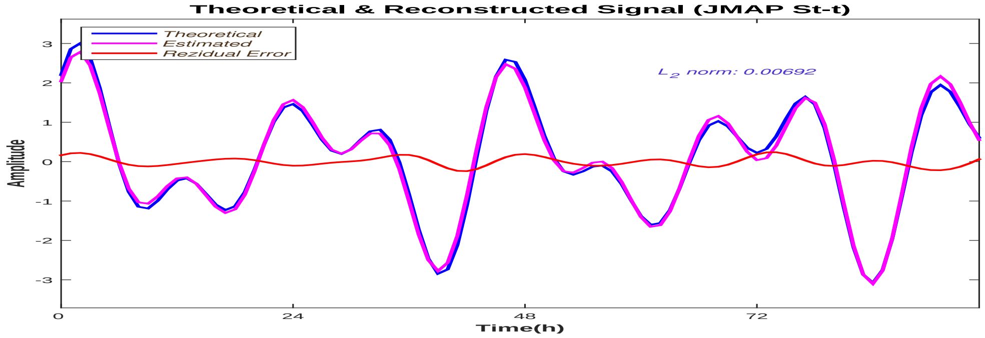

(b) Theoretical and Estimated Signal

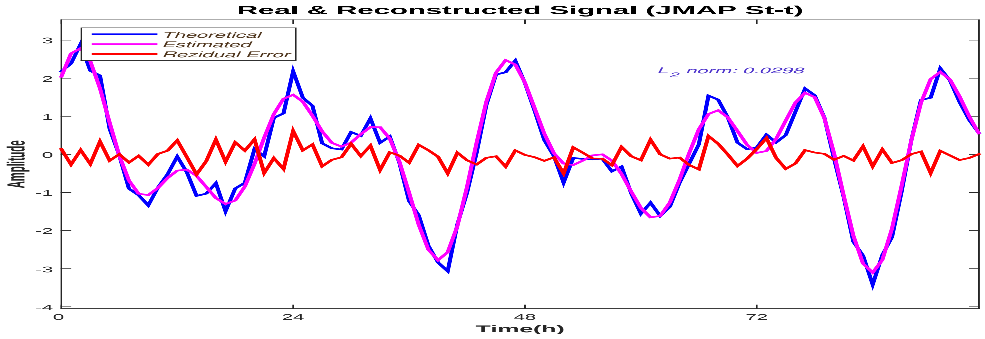

(c) Real and Estimated Signal

Figure 12: JMAP IGSM Estimation (15dB)

Figure (13a) presents the variation of L_2 PC vector error reconstruction for 10 different noise realisation, showing

high variations. Important variations corresponding to the L_2 error reconstruction for the theoretical signal $\boldsymbol{g}_0$, and signal $\boldsymbol{g}$, are presented in Figure (13b) and Figure (13c).

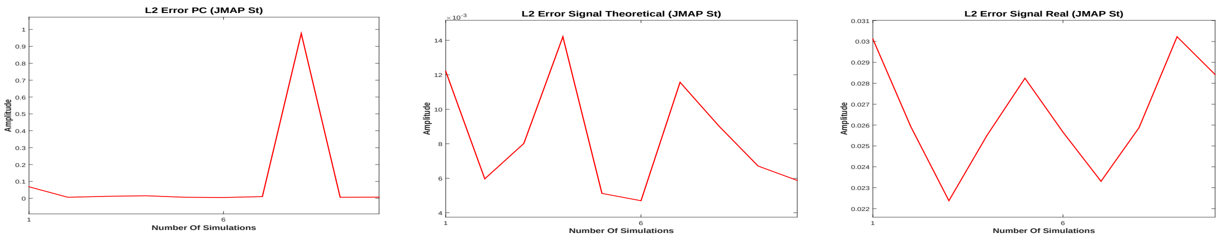

(a) L_2 Error: $\boldsymbol{f}$ vs. $\widehat{\boldsymbol{f}}_{JMAP}$ (b) L_2 Error: $\boldsymbol{g}_0$ vs. $\widehat{\boldsymbol{g}}_{JMAP}$ (c) L_2 Error: $\boldsymbol{g}$ vs. $\widehat{\boldsymbol{g}}_{JMAP}$

Figure 13: JMAP IGSM L_2 error measured for 10 different noise realisations (15dB)

3.3 PM (via VBA, partial separability) IGSM 15dB

For the Posterior Mean estimation via VBA, both the PC estimation and theoretical signal $\boldsymbol{g}_0$ reconstruction are very accurate, Figure (14a) and Figure (14b). For the reconstruction of the theoretical signal $\boldsymbol{g}_0$, the L_2 error norm is $\delta \boldsymbol{g}_0 = \frac{\|\boldsymbol{g}_0 - \widehat{\boldsymbol{g}}_{PM}\|_2^2}{\|\boldsymbol{g}_0\|_2^2} = 0.00513$. For the PC vector, the reconstruction error is $\delta \boldsymbol{f} = \frac{\|\boldsymbol{f} - \widehat{\boldsymbol{f}}_{PM}\|_2^2}{\|\boldsymbol{f}\|_2^2} = 0.00437$. The algorithm is converging to a sparse solution where all the non zero clocks are detected. The residual error computed between $\boldsymbol{g}$ and the reconstructed signal is consistent with the error considered in the model, 15dB, Figure (14c).

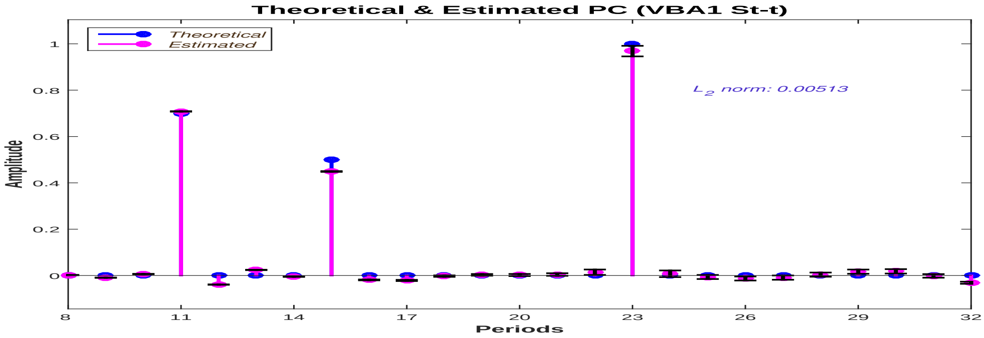

(a) Theoretical and Estimated PC

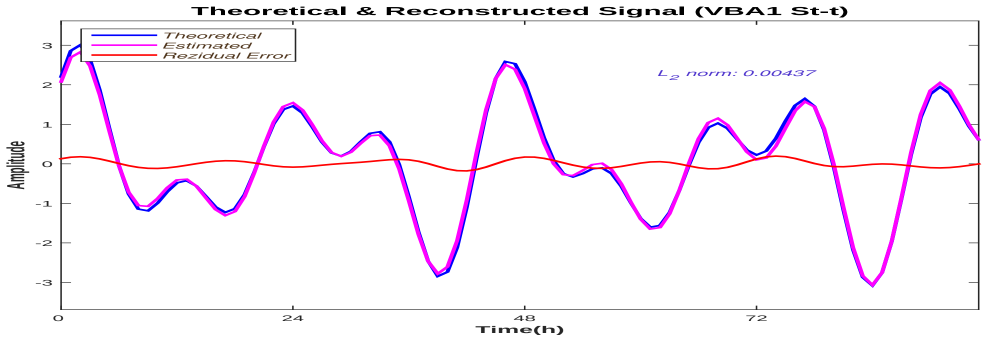

(b) Theoretical and Estimated Signal

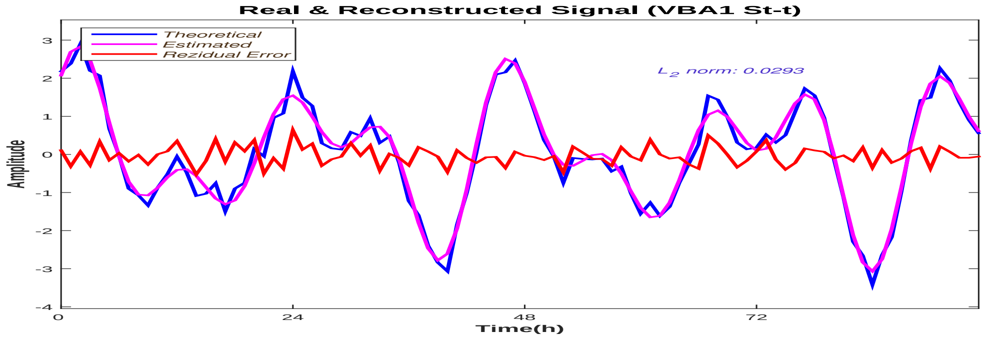

(c) Real and Estimated Signal

Figure 14: PM (via VBA, partial separability) IGSM Estimation (15dB)

The convergency of $\boldsymbol{\Sigma}$ covariance matrix diagonal, Figure (15a) and the convergency of algorithm's solution $\boldsymbol{f}$, Figure (15b) is showing a very fast convergency. All the estimates are superposed after the first ten iterations.

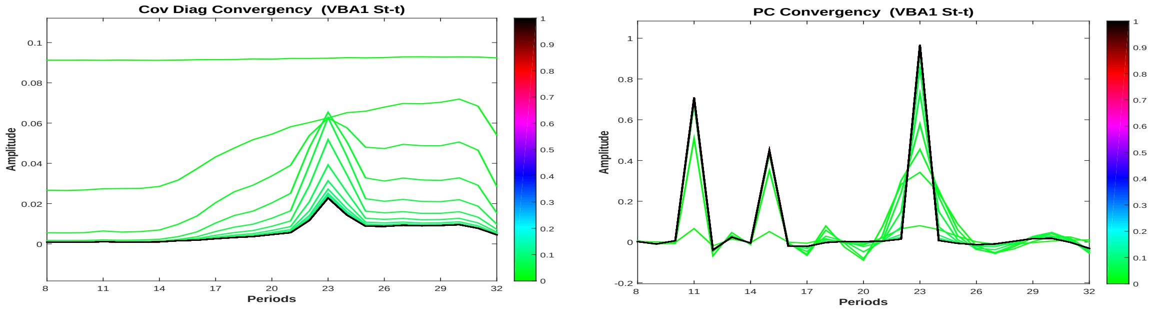

(a) $\text{diag}(\widehat{\boldsymbol{\Sigma}})$ convergency

(b) $\boldsymbol{f}$ convergency

Figure 15: PM (via VBA, partial separability) IGSM hyperparameters and $\boldsymbol{f}$ convergency

We show that for the PM estimation, the error variation is very small. Figure (16a) presents the variation of L_2 PC vector error reconstruction for 10 different noise realisation. The figure presents a very small variation of L_2 PC vector error reconstruction, between 0.003 and 0.01. Very small variations corresponding to the L_2 error reconstruction for the theoretical signal $\boldsymbol{g}_0$, and signal $\boldsymbol{g}$, are presented in Figure (16b) and Figure (16c).

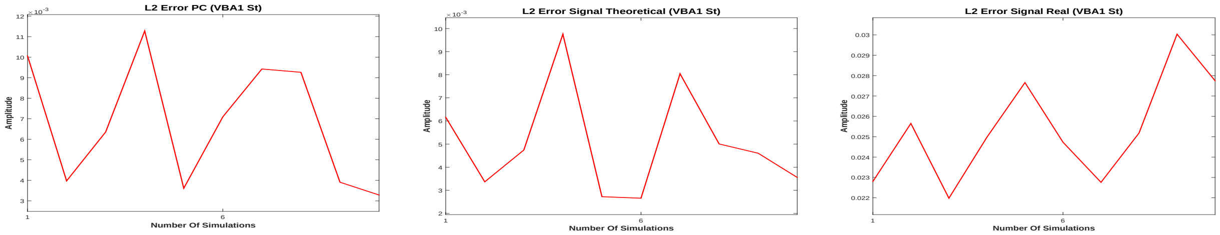

(a) L_2 Error: $\boldsymbol{f}$ vs. $\widehat{\boldsymbol{f}}_{PM}$

(b) L_2 Error: $\boldsymbol{g}_0$ vs. $\widehat{\boldsymbol{g}}_{PM}$

(c) L_2 Error: $\boldsymbol{g}$ vs. $\widehat{\boldsymbol{g}}_{PM}$

Figure 16: PM (via VBA, partial separability) IGSM L_2 error measured for 10 different noise realisations (15dB)

3.4 PM (via VBA, full separability) IGSM 15dB

The estimations for the full separability case are also accurate:

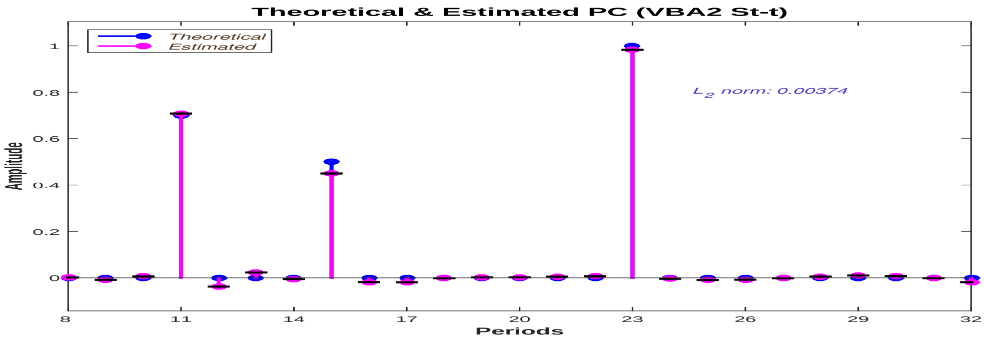

(a) Theoretical and Estimated PC

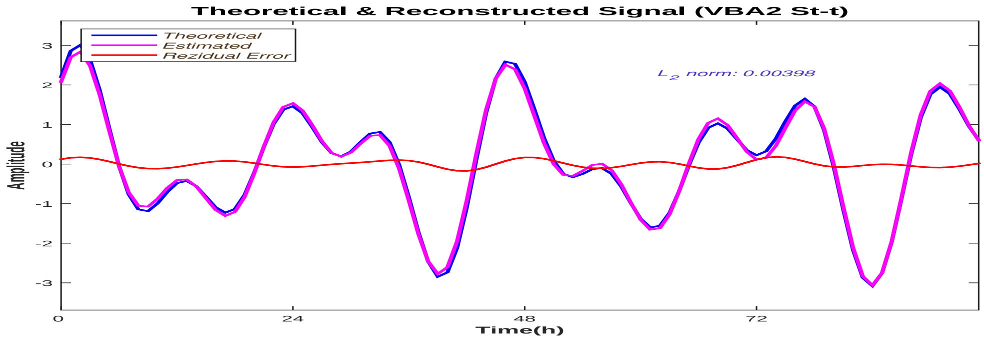

(b) Theoretical and Estimated Signal

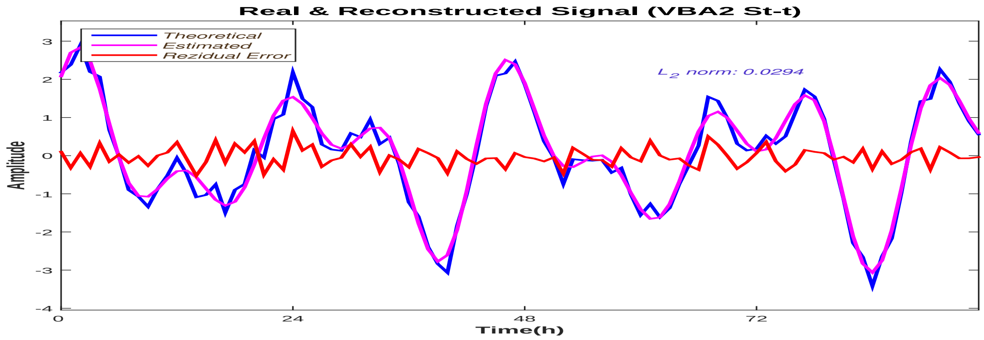

(c) Real and Estimated Signal

Figure 17: PM (via VBA, full separability) IGSM Estimation (15dB)

For the reconstruction of the theoretical signal $\boldsymbol{g}_0$, the L_2 error norm is $\delta \boldsymbol{g}_0 = \frac{\|\boldsymbol{g}_0 - \widehat{\boldsymbol{g}}_{PM}\|_2^2}{\|\boldsymbol{g}_0\|_2^2} = 0.00398$. For the PC vector, the reconstruction error is $\delta \boldsymbol{f} = \frac{\|\boldsymbol{f} - \widehat{\boldsymbol{f}}_{PM}\|_2^2}{\|\boldsymbol{f}\|_2^2} = 0.00374$.

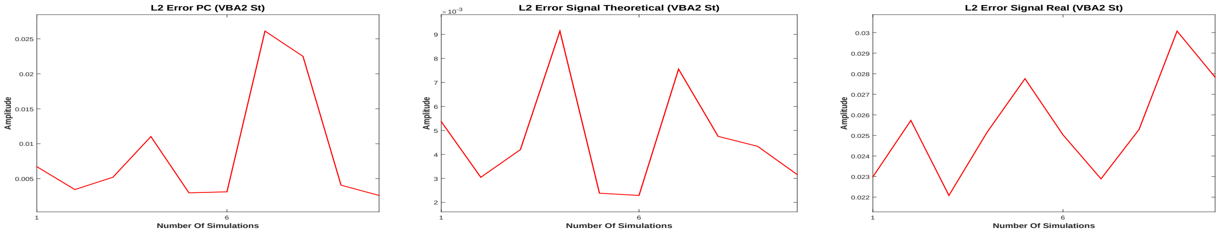

(a) Theoretical and Estimated PC (b) Theoretical and Estimated Signal (c) Real and Estimated Signal

Figure 18: PM (via VBA, full separability) IGSM L_2 error measured for 10 different noise realisations (15dB)

Figure (18a) presents the variation of L_2 PC vector error reconstruction for 10 different noise realisation. The figure presents a very small variation of L_2 PC vector error reconstruction, between 0.0037 and 0.027. Very small variations corresponding to the L_2 error reconstruction for the theoretical signal $\boldsymbol{g}_0$, and signal $\boldsymbol{g}$, are presented in Figure (18b) and Figure (18c).

3.5 Methods comparison 15dB

The comparison between the estimations corresponding to the the proposed IGSM models is presented in Figure (19c) (JMAP estimator), Figure (19d) (PM via VBA, partial separability estimator) and Figure (19e) (PM via VBA, full separability estimator). The comparison with the Gaussian case (i.e. Gaussian prior), via the two estimators discussed, is presented in Figure (19a) (Gaussian Model, JMAP estimator) and Figure (19b) (PM via VBA estimator). A comparison with the FFT is presented in Figure (19f).

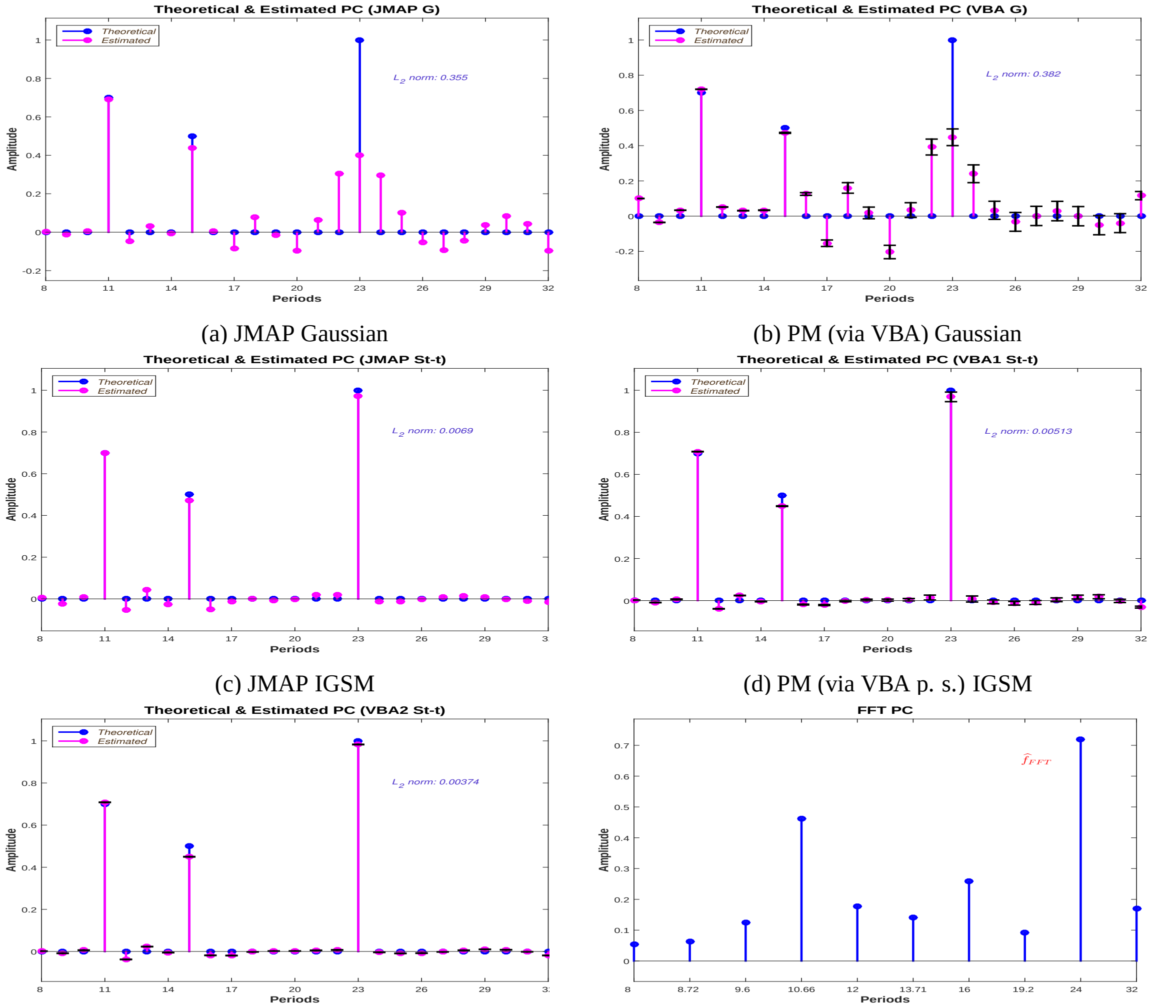

(a) JMAP Gaussian

(b) PM (via VBA) Gaussian

(c) JMAP IGSM

(d) PM (via VBA p. s.) IGSM

(e) PM (via VBA f. s.) IGSM

(f) FFT

Figure 19: Methods Comparison (15dB)

The L_2 estimation error for the PC vector is very high for the two Gaussian models. Also, the estimations are not sparse. For the IGSM models, the JMAP estimator is providing a good estimation, but it is unstable. VBA estimator, both partial separable and fully separable provides very accurate stable estimations.

3.6 Error comparison 15dB

The L_2 error measurement corresponding to the PC estimation, theoretical signal estimation and the signal estimation, for 10 different noise realisation is presented in the following figure:

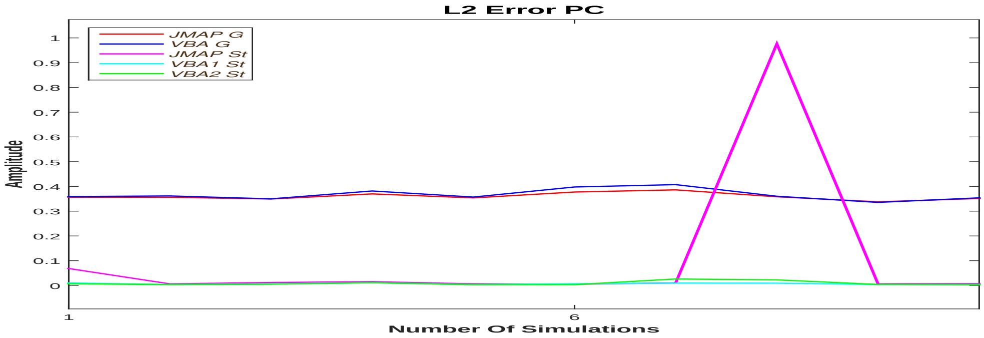

(a) PC error estimation

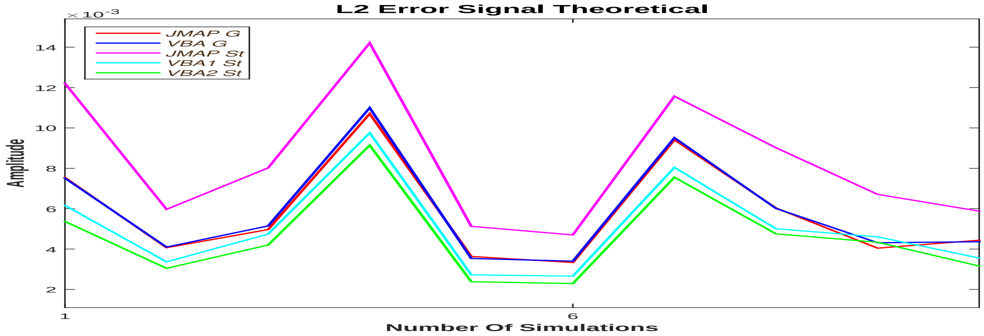

(b) Theoretical signal error estimation

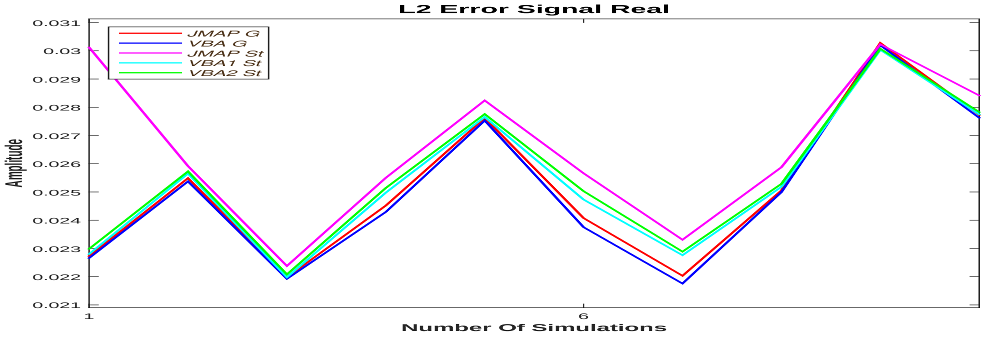

(c) Signal error estimation (15dB)

Figure 20: L_2 Errors estimation (15dB)

The $L2$ error corresponding to the PM via VBA IGSM Model shows the performances of proposed via algorithm compared to the Gaussian Model and the JMAP estimation for IGSM Model.

References
